# BglBrick vectors and datasheets: A synthetic biology platform for gene expression

**DOI:** 10.1186/1754-1611-5-12

**Published:** 2011-09-20

**Authors:** Taek Soon Lee, Rachel A Krupa, Fuzhong Zhang, Meghdad Hajimorad, William J Holtz, Nilu Prasad, Sung Kuk Lee, Jay D Keasling

**Affiliations:** 1 Joint BioEnergy Institute, 5885 Hollis St., Emeryville, CA 94608, USA; 2 Physical Biosciences Division, Lawrence Berkeley National Laboratory, Berkeley, CA 94720, USA; 3 Department of Chemical & Biomolecular Engineering, University of California, Berkeley, CA 94720, USA; 4 Department of Bioengineering, University of California, Berkeley, CA 94720, USA; 5 Department of Electrical Engineering, University of California, Berkeley, CA 94720, USA; 6 Synthetic Biology Engineering Research Center, University of California, Berkeley, CA, 94720, USA; 7 Current Address: Schools of Nano-Bioscience & Chemical Engineering, Ulsan National Institute of Science and Technology, Ulsan, Korea

## Abstract

**Background:**

As engineered biological systems become more complex, it is increasingly common to express multiple operons from different plasmids and inducible expression systems within a single host cell. Optimizing such systems often requires screening combinations of origins of replication, expression systems, and antibiotic markers. This procedure is hampered by a lack of quantitative data on how these components behave when more than one origin of replication or expression system are used simultaneously. Additionally, this process can be time consuming as it often requires the creation of new vectors or cloning into existing but disparate vectors.

**Results:**

Here, we report the development and characterization of a library of expression vectors compatible with the BglBrick standard (BBF RFC 21). We have designed and constructed 96 BglBrick-compatible plasmids with a combination of replication origins, antibiotic resistance genes, and inducible promoters. These plasmids were characterized over a range of inducer concentrations, in the presence of non-cognate inducer molecules, and with several growth media, and their characteristics were documented in a standard format datasheet. A three plasmid system was used to investigate the impact of multiple origins of replication on plasmid copy number.

**Conclusions:**

The standardized collection of vectors presented here allows the user to rapidly construct and test the expression of genes with various combinations of promoter strength, inducible expression system, copy number, and antibiotic resistance. The quantitative datasheets created for these vectors will increase the predictability of gene expression, especially when multiple plasmids and inducers are utilized.

## Background

Metabolic engineering, the redirection of metabolic pathways using genetic manipulation, plays an important role in a wide range of biological research including drug production, bioremediation, and biofuel production [1-5]. Metabolic pathways that lead to important drugs or chemicals are often multi-step processes involving many enzymes. In addition, controlling and coordinating the activity of each enzyme to achieve the optimal production of the target product is extremely complicated [6-9]. To construct an entire metabolic pathway in a heterologous host, the genes encoding the pathway enzymes often have to be constructed on multiple plasmids. Furthermore, the expression of each enzyme needs to be tuned to balance it with that of the other enzymes in the pathway and to reduce the metabolic burden on the host cell [6,9-11]. Recently, several advanced cloning methods using homologous recombination, such as Sequence and Ligation-Independent Cloning (SLIC), Gibson DNA assembly, and 'DNA assembler', have been reported and applied to construct large plasmids or chromosomes that encode metabolic pathways [12-14]. However, these methods require non-standardized homologous complementary sequences for each gene part and are limited in terms of automation and the number of DNA fragments to be assembled to build a combinatorial library for pathway optimization.

Synthetic biology is an emerging field with large potential in engineering biological systems and has been a powerful tool for metabolic engineering [2,15-17]. Synthetic biology focuses on the design and construction of biological parts that can be understood, designed, and tuned to meet specific performance criteria. These parts are then assembled into larger integrated systems to solve specific problems [15,18].

**Figure 1:**
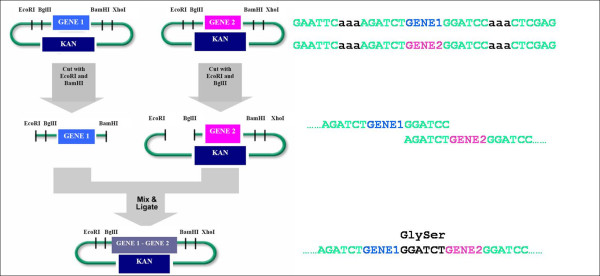
**Schematic diagram of the BglBrick part assembly**. Four unique restriction sites (*EcoRI, BglII, BamHI*, and *XhoI*) are used for the BglBrick standard assembly. KAN is the kanamycin resistance marker.

The standardization of biological parts and their assembly is one of the core ideas behind synthetic biology. To achieve this with parts (e.g., ribosome binding sites, promoters, DNA binding proteins, etc.) researchers at MIT established the BioBricks™ standard (so called BBF RFC 10) [19]. BglBrick standard (so called BBF RFC 21) is a recently proposed standard that uses 4 unique restriction enzyme sites (*EcoRI, BglII*, and *BamHI, XhoI*) different from BioBricks™ standard [20] (Figure 1). The standardized assembly approach such as BioBricks or BglBricks does not require PCR amplification step and consequently, the post-assembly sequence verification step is optional. It is especially useful when constructing metabolic pathways that are encoded by many genes and need to be assembled in various combinations to search for improved phenotypes.

The construction of vectors using BioBricks™ standard biological parts has been reported recently [21]. These vectors were constructed by various combinations of BioBricks™ compatible parts and BioBrick™ base vector (BBa_I51020) using BioBricks™ gene assembly protocol. In metabolic engineering research, several sets of such vectors--with different combinations of replication origin, promoter, and antibiotic resistance markers--would be a very useful tool to test and optimize the production of specific target molecules. In this report, we describe a large set of vectors that are compatible with BglBrick parts. These vectors contain commonly used replication origins, inducible gene expression systems, and antibiotic resistance markers. After constructing these expression vectors, we tested and quantified their ability to express fluorescent proteins that were spliced into them. The resulting information is presented in datasheets that will allow engineers to design metabolic pathways with greater control.

## Results

### Construction of BglBrick vectors

#### Construction of intermediate BglBrick vectors

**Figure 2:**
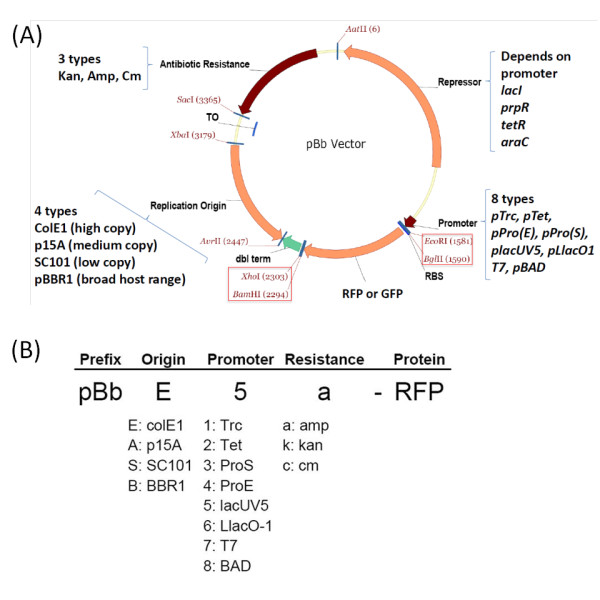
**Plasmid design and nomenclature of BglBrick plasmids (pBb)**. (A) Plasmid design of pBb vectors. The plasmid is composed of three modules: antibiotic resistance gene module, replication origin module, and expression module, which includes the repressor, promoter, gene of interest (*rfp *or *gfp*), and terminator. BglBrick sites are in red boxes. (B) Nomenclature of the pBb vector system. The identity of the vector is described by three letters containing the information of replication origin, promoter, and antibiotic resistance marker as indicated. The prefix pBb is used for BglBrick plasmids and the protein gene name in the plasmid is included at the end of the vector description.

To construct a set of vectors compatible with BglBrick sites, we have chosen four different replication origins belonging to different incompatibility groups and having different copy numbers. The ColE1 replication origin has been used to make a relatively high copy version of the vector, the p15A origin for a medium copy version, the SC101 origin for a low copy version, and the pBBR1, a broad host range origin, for a second medium copy version. (Figure 2) We have introduced a few point mutations into the Rep gene of pBBR1 origin to engineer a copy number about 6-fold higher than the non-mutated origin as previously reported [22].

Since some of the vector components have BglBrick restriction sites (*EcoRI, BglII, BamHI, XhoI*) in their original sequence, we had to mutate them to remove these sites. Site-directed mutagenesis was used to make a single nucleotide substitution to eliminate the restriction sites from these components in the intermediate plasmids, and when the mutation site occurred within an open reading frame, *E. coli *codon usage was taken into consideration.

#### Construction of expression module

The promoter system modules consisted of one of 8 different promoters and corresponding repressors. We have chosen the promoters that have been frequently used for protein production and metabolic pathway engineering. For IPTG-inducible promoters, the Trc and T7 promoters were chosen as strong promoters and P_lacUV5 _as medium strength promoter [23]. Each construct carried *lacI^q ^*to repress expression from these promoters. P_LlacO-1 _was also added to the IPTG-inducible promoter list because of its tight regulation with medium- to high-level expression. Several promoters induced by chemicals other than IPTG were included in the promoter list: tetracycline-regulated gene expression system (P_tet _and *tetR*) [24], the recently developed propionate-regulated gene expression system (P_prpB _and *prpR*) [25,26], and the arabinose-inducible promoter system (P_BAD _and *araC*) [27]. The list of promoters is described in Figure 2(B).

#### Construction of promoter-*rfp*-terminator module and final pBb vector assembly

The modules with promoter system and *rfp*-terminator were constructed by SOE-PCR. Each vector contains a 5'-UTR and *rfp *between the *BglII* and *BamHI* sites. This operon was used to characterize expression from the vectors and can be used to screen against background vector when cloning other operons into these vectors. We have designed a systematic naming rule for these BglBrick vectors that describes types of replication origin, promoter, antibiotic resistance, and the gene included as a BglBrick part. This naming scheme is described in Figure 2(B).

### Data sheet experiments

Performing a quantitative characterization of a biological parts and then summarizing the properties of the parts on datasheets has been previously described [18]. A similar type of datasheet should be useful when the BglBrick vectors are used to produce a single target protein or all of the enzymes in an entire metabolic pathway. A summary of gene expression and cell growth behavior of a specific plasmid in a specific host strain can be a valuable resource for determining which origin-promoter-resistance combination would be most useful for a particular metabolic engineering project.

**Figure 3:**
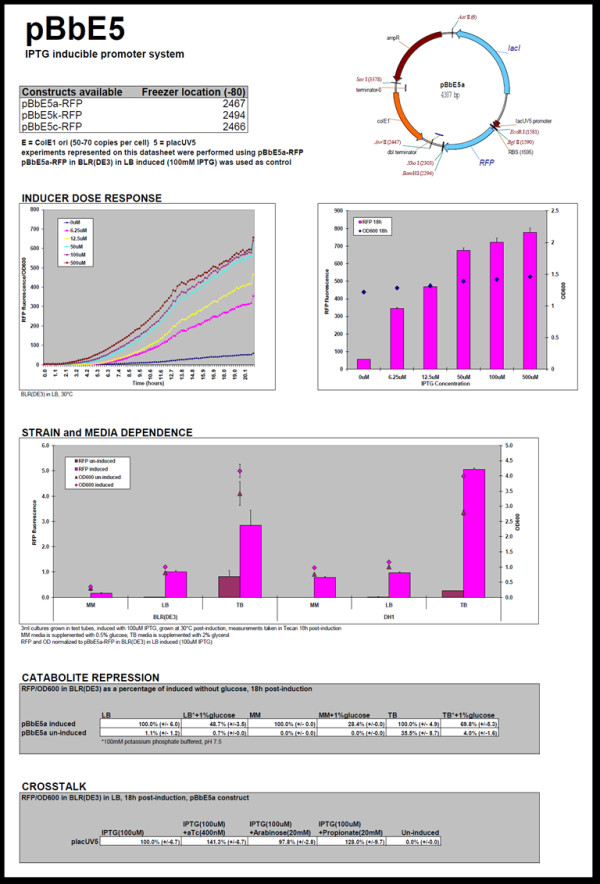
**Datasheet for the pBbE5 vector**. The datasheet includes a general description of BglBrick vector and a summary of its expression properties.

We prepared 32 datasheets for the vectors that have different replication origins and promoters. The PDF files of datasheets are currently available from the JBEI Public Registry (https://public-registry.jbei.org) and also as Additional file 1 (an example of the datasheet is shown in Figure 3). We used only ampicillin-resistant BglBrick vectors for the datasheet experiments based on the assumption that antibiotic resistance does not significantly affect the expression property and copy number of BglBrick vectors [28]. In the datasheet, we included a plasmid map and the experimentally-determined copy number, expression, and growth properties of *E. coli *strains harboring a specific BglBrick plasmid under various conditions (several inducer concentrations, different types of culture medium, high glucose concentration for catabolite repression, and the presence of other inducers that might alter expression from the target promoter)[29].

#### Data sheet experiment: inducer dose response

The level of protein production usually depends on inducer concentration, and this expression profile is important information when using the BglBrick vectors for metabolic engineering, which frequently requires tight control of the metabolic pathway. RFP expression (or GFP expression for pBbB vectors) at different inducer doses was tested in *E. coli *BLR(DE3) grown in LB medium with ampicillin. pBbE5a-RFP in LB-ampicillin medium induced with 100 μM IPTG was used as a control for all the measurements (pBbE5a-GFP was used as a control for pBbB vectors). The concentration range for each inducer was determined based on simple scanning over a wide range of inducer concentrations with the high copy (ColE1 origin) BglBrick plasmid. For IPTG-inducible systems, the production of fluorescent protein was monitored at up to 500 μM IPTG, and for plasmids with the Tet-inducible promoter, propionate-inducible promoter, and arabinose-inducible promoter, the production of fluorescent protein was monitored at up to 200 nM of aTc, 100 mM of propionate, and 20 mM of arabinose, respectively. A plot containing average and specific fluorescence (fluorescence from RFP or GFP/OD_600_) as a function of time was created for each inducer concentration, and the data were also presented as a bar graph at different inducer concentration at a single time point (18 hrs after induction) (Figure 3). Most BglBrick plasmids showed clear dose-dependent expression (Figure 3 and Additional file 1 for more datasheets).

#### Data sheet experiments: strain and medium dependence

Strain and medium dependence of BglBrick plasmids are important properties to consider when selecting the vectors and the medium for metabolic pathway expression, and they were examined in two frequently used *E. coli *strains (BLR(DE3) and DH1). Three different growth media (minimal medium (M9) and rich media (LB and TB)) were used to test fluorescence protein production from these two strains. Most BglBrick vectors, except those with propionate inducible promoters, showed almost no strain dependence with the strongest expression in TB medium and the weakest expression in minimal medium in general, as expected. Plasmids containing the T7 promoter were not tested in DH1 since DH1 lacks T7 RNA polymerase.

The propionate-inducible promoters showed unusual behavior in different strains and in different media. For example, pBb{A, E, or S}3a-rfp (or pBbB3a-gfp) plasmids in BLR(DE3) showed normal expression behavior in LB medium but almost no expression in TB medium. Recently, it has been found that in addition to the native inducer, propionate, this promoter system is also regulated by carbon catabolite repression (CCR) mediated by several sugars like glucose, arabinose, mannose, xylose, galactose and glycerol [24]. This CCR mediated regulation could be the reason for the lack of expression from these plasmids in BLR(DE3) in TB medium as the medium contains 0.2% glycerol. Interestingly, when the same vectors were tested in DH1, fluorescent protein was produced both in LB and TB media. However, expression was very leaky in these media; protein was produced regardless of the concentration of propionate. These results indicate that the propionate inducible expression systems are host-dependent.

#### Data sheet experiments: catabolite repression and inducer crosstalk

Carbon catabolite repression (CCR) in *E. coli *is a regulatory mechanism to ensure sequential utilization of carbohydrates [30]. In metabolic engineering, glucose is frequently supplemented at high concentration in the medium as the primary or only carbon source, and it is important to know whether the transcriptional machinery of the pathway works normally in the presence of high concentrations of glucose. The effect of glucose for each BglBrick plasmid (in BLR(DE3)) was tested in the three different media containing 1% glucose. As control experiments, the media without additional glucose were also used for the expression of fluorescent protein with or without inducer. All the vectors with a version of the lac promoter were repressed by the addition of 1% glucose. Leaky expression from these plasmids in TB medium decreased dramatically in presence of additional glucose. Vectors with the tetracyclin-inducible promoter were less susceptible to catabolite repression, showing only about 20% less protein production when expressed in LB with 1% glucose compared to the production in LB without additional glucose. Vectors with the arabinose-inducible promoter were also repressed by glucose, and more repression was observed in LB with 1% glucose than in the other media tested. Vectors with propionate-inducible promoters (promoter numbers 3 and 4) were very strongly repressed by the presence of 1% glucose in both LB and TB media. But interestingly, they showed less repression in minimal medium when 1% glucose was added.

To optimize or balance the expression of proteins in a metabolic pathway, the pathway genes are frequently placed under control of different promoters, each of which may use a unique inducer to regulate transcription. Unfortunately, there can be substantial crosstalk among some inducible systems that makes independent regulation difficult [29]. The potential crosstalk between various inducible promoters in BglBrick plasmids was tested at the inducer concentrations that achieved the highest protein expression in LB medium with BLR(DE3). Some IPTG-inducible systems (P_trc _and P_lacUV5_) did not show any crosstalk in the presence of 20 mM arabinose, but P_LlacO1 _and P_T7 _showed about 15-20% decrease in fluorescent protein production when 20 mM arabinose was added to the medium. Also, P_trc _and P_T7 _did not show any crosstalk toward aTc and propionate, but P_lacUV5 _and P_LlacO1 _showed about 30-40% increase in protein production in the presence of 400 nM aTc or 20 mM propionate. P_tet _performance decreased about 10-15% when 20 mM arabinose or 20 mM propionate was added, but was not affected by the presence of 100 μM IPTG. The propionate-inducible expression system did not function well in the presence of 20 mM arabinose: expression from P_pro _in the presence of arabinose was 20-30% of that in the absence of arabinose. BglBrick plasmids with P_BAD _showed the most independent behavior in the presence of other inducers.

The mechanism by which arabinose represses the non-P_BAD _promoters is not clear, but one potential explanation could be the decreased level of cAMP-CRP in the presence of arabinose. As endogenous arabinose promoters are turned on by the addition of arabinose, the cAMP-CRP concentration may decrease since the cAMP-CRP also binds to the *araB *promoter and the cell does not have enough cAMP-CRP to bind to the lac promoter or propionate-inducible promoter. This still cannot explain the repression of P_tet _by high level of arabinose, and more studies on the crosstalk of these promoters and inducers needs to be conducted.

#### Data sheet experiment: Plasmid copy number determination

**Figure 4:**
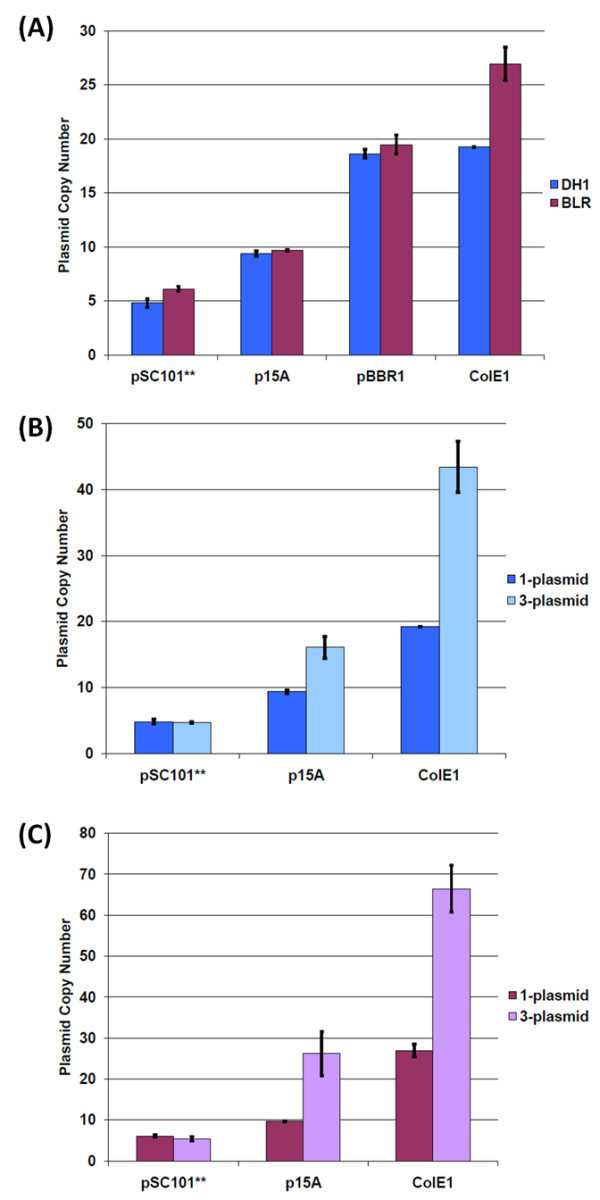
**BglBrick plasmid copy numbers in DH1 and BLR(DE3)**. The blue bars are for DH1 strains, and the purple bars are for BLR(DE3) strains. The dark colored bars are for the single-plasmid strain, and the light colored bars are for the three-plasmid strain containing pBbA8a-CFP, pBbE5c-YFP and pBbS2k-RFP. Plasmids with the pBBR1 origin were not tested in the three-plasmid strain. (A) Plasmid copy number for single plasmid strain (B) DH1 plasmid copy number comparison for single and three plasmid strain (C) BLR(DE3) plasmid copy number comparison for single and three plasmid strain.

Real-time quantitative PCR was used to determine the copy numbers of plasmids [22,31-35] with the available origins of replication in our system. Copy numbers were determined for plasmids transformed into both *E. coli *BLR and DH1. The single copy *nptII *gene harbored on the plasmid was used as the target to measure plasmid copy number, with the multi-copy 16S rDNA gene harbored on host chromosome [36] having been used for normalization purposes [22,34,35]. Here, plasmid copy is defined as the number of copies of plasmid present per chromosomal equivalent in *E. coli *[37], and absolute plasmid copy numbers were obtained by using BLR and DH1 transgenic strains containing a single *nptII *(integrated into the *intA *site on the chromosome of DH1 and into the *tyrR *site on the chromosome of BLR) as the reference sample. The plasmid copy numbers obtained for strains with a single plasmid of a particular replication origin are comparable to those found in the literature (Figure 4(A)) [38,39]. While the pSC101** origin is a derivative of pMPP6 [40], we have found comparable copy numbers for plasmids with wild-type pSC101, pMPP6, or pSC101** origins of replication (data not shown). It is also worth noting that similar copy numbers were obtained for a given plasmid transformed into either *E. coli *BLR or DH1 (Figure 4(A)), suggesting that these two strains do not differentially impact the regulation of plasmid copy number for the replication origins employed here.

From the results of strain and media dependence, catabolite repression, and inducer crosstalk experiments, the propionate inducible vectors may not be a good choice to be used in metabolic engineering for chemical or biofuel production, both of which frequently require high levels of carbon source and multiple plasmids under different expression machinery to optimize production. The propionate system has been reported to be useful for toxic protein production [26], but additional studies on its expression and regulation would be informative for its use in more complex systems.

### Application of BglBrick plasmids

#### Expression of different fluorescent proteins from multiple plasmids in the same cell

The next step was to test the application of multiple BglBrick plasmids for orthogonal protein expression, and we chose BlgBrick plasmids with P_BAD_, P_lacUV5 _and P_tet _promoters for these studies. To examine the expression control using these vectors, we tested the orthogonal protein production of the BglBrick vector system using three different fluorescent proteins. Each *E. coli *BLR(DE3) cell carried three BglBrick vectors, each with a unique replication origin, antibiotic resistance, and promoter: pBbA8a-CFP, pBbE5c-YFP and pBbS2k-RFP. These vectors contained *cfp, yfp*, and *rfp *under the control of P_BAD_, P_lacUV5_, and P_tet_, respectively. Fluorescence excitation and emission wavelengths were carefully chosen so that there would be little overlap of fluorescence excitation and emission spectra between CFP, YFP, and RFP fluorescence detection.

**Figure 5:**
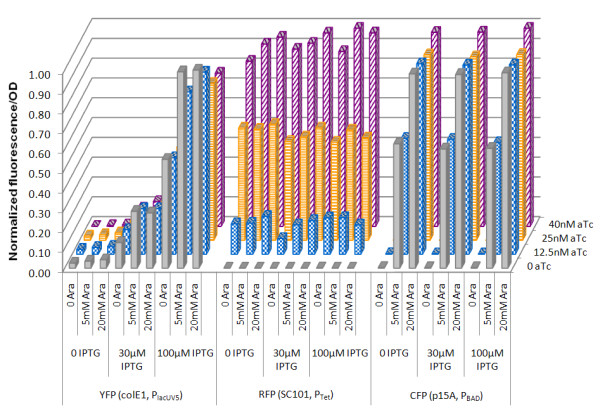
**Expression of three different proteins from a single strain at various inducer concentrations**. pBbA8a-*cfp*, pBbE5c-*yfp *and pBbS2k-*rfp *were transformed in *E. coli *BLR(DE3) and the fluorescent proteins were expressed under various inducer concentration combinations.

When CFP fluorescence was measured, a clear dependence on arabinose concentration from 0 (low) to 5 mM (medium) to 20 mM (high) was observed (Figure 5). At the same arabinose concentration, variation in IPTG and aTc concentration had no effect on CFP fluorescence, indicating that P_BAD _in the BglBrick vector system was only inducible by arabinose, but not by IPTG and aTc, at least at the tested concentrations. Similar results were obtained for RFP fluorescence. At four tested aTc concentrations, *rfp *exhibited low (0 aTc), medium-low (12.5 nM aTc), medium-high (25 nM aTc), and high (40 nM aTc) expression. Both IPTG and arabinose had no significant effect on RFP fluorescence, indicating that P_tet _is only responsive to aTc.

For P_lacUV5_, YFP fluorescence increased with IPTG concentration, confirming that P_lacUV5 _is responsive to IPTG. However, we also observed that increasing the arabinose concentration slightly increased the YFP fluorescence. This was not caused by the CFP's contribution to the YFP fluorescence signal, because otherwise stronger arabinose dependence would be expected in the absence of IPTG since low YFP expression would make the effect most apparent. On the other hand, increasing aTc concentration slightly decreased YFP fluorescence. This is also not due to the RFP's contribution to the YFP fluorescence, because the opposite effect - increasing in YFP fluorescence as aTc increases - would be expected. Previously, crosstalk between the IPTG-inducible P_lac _and the arabinose-inducible P_BAD _was observed but the molecular mechanism remains unclear [29]. One possible reason for this crosstalk may be the non-specific binding of AraC or TetR to P_lacUV5_. Regardless of the cause, the most apparent dependence of YFP fluorescence is on IPTG concentration. Overall, we demonstrated that three promoters from the BglBrick vectors can be orthogonally induced.

#### Copy number of plasmid in multiple plasmid strain

In order to assess the impact of metabolic burden on plasmid copy number, we determined the copy number of individual plasmids in a cell harboring all three plasmids. Here, *E. coli *BLR and DH1 were co-transformed with pSC101**-, p15A-, and ColE1-containing plasmids. Each plasmid harbored a different selection marker (resistance to ampicillin, chloramphenicol, or kanamycin), and cultures were grown in media supplemented with all three antibiotics. Using real-time quantitative PCR, absolute copy number was determined for the plasmid harboring the kanamycin selection marker *nptII*. The results obtained (Figure 4(B), (C)) indicate that plasmids containing the p15A and ColE1 origins had a higher copy number in cells harboring all three plasmids than in cells harboring a single plasmid. For pSC101**, on the other hand, the copy number was unchanged from that observed under single plasmid conditions. Our results are consistent with those found in literature [28,41] and may be explained by differences in the replication control mechanism of the origins. As a broad generalization, plasmid replication control is either relaxed or stringent, with plasmid replication being uncoupled from host chromosomal DNA synthesis in the former case [42]. ColE1 and related plasmids (which includes p15A) replicate under relaxed control while pSC101 is stringent [43]. Consistent with our results (Figure 4), variation in pSC101 copy number is thus not to be expected under stress conditions because plasmid replication is tightly coupled to the bacterial cell cycle.

## Conclusion and Discussion

Synthetic biology provides a powerful tool that can be applied to a variety of goals: engineering metabolic pathways, overproducing a specific protein, examining fundamental biology. In this report, we describe expression vectors that would be useful to researchers using BglBrick standard to express single genes or entire metabolic pathways. We assembled a library of expression vectors to be compatible with a recently-developed BglBrick standard, and as a result, any DNA sequences with BglBrick standard format can be cloned into these vectors. In addition, we designed the vectors to allow for precise control of the expression of multiple genes, whether that be to construct a metabolic pathway or for any other multi-gene expression project. The datasheets for these BglBrick vectors provide information about their expression properties under various conditions (e.g., medium, strain, and with different inducers). These datasheets will serve as an initial point of reference when designing and optimizing complex gene expression systems.

In the report, we demonstrated the compatible and controllable aspects of the vectors with fluorescent proteins as a model system only. In addition, we have used these vectors to construct complex metabolic pathways, such as the isoprenoid biosynthetic pathway [44], and to express a library of membrane transporter proteins [45]. These works involved the transformation of host cells with either multiple plasmids or single plasmid containing different genes expressed at various levels.

All the BglBrick vectors and their datasheets have been deposited in Joint BioEnergy Institute Public Registry (https://public-registry.jbei.org) and are available for searching and reviewing the sequences and annotations. We also made these vector strains and datasheets available through institutional strain distributor and a non-profit strain distributing organization, such as Addgene (http://www.addgene.org) with an appropriate material transferring process.

To expand the use of these vectors for further application, various additional biological parts can be designed with the BglBrick format. For example, new parts composed of a combination of various terminators and promoters have been designed to make multiple operon variants of BglBrick plasmids (data not shown). These parts can be appended or prepended either to original BglBrick vectors or to BglBrick plasmids already containing pathway genes to allow co-expression of different pathways or genes from the same plasmid. In addition, variants of the BglBrick plasmids described here that do not include any specific promoter-repressor components have been prepared (Additional file 2). These variants would expand the use of BglBrick vectors for application with various natural or synthetic promoters.

Finally, the concept of standardized biological parts allows automated assembly of recombinant DNA and will likely prove to be a powerful tool in engineering biological systems. Recently, the automated DNA assembly using BglBrick standard and 2 antibiotic (2ab) assembly strategy has been reported [46]. In this report, an automated assembly protocol was introduced for high throughput parallel assembly of BglBrick part DNAs. The BglBrick vectors we report here are compatible to this automation strategy and can also be used for assembling various combinations of pathway genes for the screening to optimize any target metabolic pathway.

## Methods

### Strains and plasmids

*E. coli *DH10B was used for cloning. *E. coli *BLR (DE3) and DH1 were used for expression studies with BglBrick vectors. Plasmids and BglBrick parts used in this study are listed in Table 1. Media were supplemented with 100 μg/mL ampicillin, 35 μg/mL chloramphenicol, or 50 μg/mL kanamycin to select for plasmid maintenance. All strains were grown at 30°C unless described otherwise.

**Table 1:** Summary of plasmids and parts used for the construction of BglBrick vector

Plasmid/Part Name	Component function	Reference
pZA31-luc	Cm	[44]
	T0 of phage lambda	
	p15A origin	
pZE12-luc	Amp	[44]
	ColE1 origin	
pZE21-MCS1	Kan	[44]
	PLlacO-1	
pZS*24-MCS1	pSC101*	[44]
pZB	Tet repressor	[29]
	Tet promoter	
pET-29b(+)	T7 promoter	Novagen
	RBS	
	lacI	
pTrc99A	Ptrc	Pharmacia
	lacIq	
pBAD33	PBAD	[32]
pPro24	pPro(E)	[30]
pPro29b	pPro(S)	[31]
BBa_E1010	rfp	[26]
BBa_B0015	double terminator	[27]
pAM45	PlacUV5	[28]
pMBIS	pBBR1 origin	[6]
pBMOE1	gfp without *BamHI *site	J. Dietrich, unpublished

### Construction of BglBrick vector parts

The template plasmids or parts for the BglBrick vectors constructed here are listed in Table 1 and the primers for PCR amplification are listed in Table 2. Each gene component has been either PCR amplified from a template using Phusion™ High-Fidelity DNA polymerase (New England BioLabs, F-530) or digested from template plasmids and incorporated into the BglBrick vector plasmid by standard restriction digestion/ligation method.

**Table 2:** Primers for BglBrick vector construction

Name	Primers for promoter system	Description
pSC101QC F1	5'- gaatttacagatacccagatcAcccgggaaaagg-3'	to remove *BglII *site on pSC101
pSC101QC R1	5'- ccttttcccgggTgatctgggtatctgtaaattc-3'	to remove *BglII *site on pSC101
pBBR1 F1	5'- gatcaCCTAGGctacagccgatagtctggaacagcgc -3'	for pBBR1 origin with *AvrII*
pBBR1 mut R1	5'- ccggcaccgtgtTggcctacgtggtc -3'	to increase copy number of pBBR1
pBBR1 mut F1	5'- gaccacgtaggccAacacggtgccgg -3'	to increase copy number of pBBR1
pBBR1 R2	5'- agatcaACTAGTgcctccggcctgcggcctgcgcgcttcg -3'	for pBBR1 origin with *SpeI*
CmQC F1	5'-ctttcattgccatacgAaattccggatgagcattc-3'	to remove *EcoRI *site on Cm^R^
CmQC R1	5'-gaatgctcatccggaattTcgtatggcaatgaaag-3'	to remove EcoRI site on Cm^R^
KanQC F1	5'- cctgtctcttgatcagatcAtgatcccctgc-3'	to remove *BglII *site on Km^R^
KanQC R1	5'- gcaggggatcaTgatctgatcaagagacagg-3'	to remove *BglII *site on Km^R^
RFP F1	5'- aaaAGATCTtttaagaaggagatatacatATGgcgagtagcgaagacgttatc-3'	for rfp with *BglII*
RFP R1	5'- CTCGAGtttGGATCCttaagcaccggtggagtgacg-3'	for rfp with *BamHI *and *XhoI*
Term F1	5'- gtgcttaaGGATCCaaaCTCGAGtaaggatctccaggcatcaaataaaacg-3'	for double terminator with *BamHI *and *XhoI*
Term R1	5'- gatcaCCTAGGtataaacgcagaaaggcccacccgaagg -3'	for double terminator with *AvrII*
pTrc F1	5'- agatcaGACGTCgacaccatcgaatggtgcaaaacc-3'	for P_trc _with *AatII*
placUV5 R1	5'- tatctccttcttaaaAGATCTtttGAATTCtgaaattgttatccgctcacaattc-3'	for P_trc_, P_lacUV5_, and P_T7 _with *EcoRI *and *BglII*
pTet F1	5'- agatcaGACGTCttaagacccactttcacatttaagttg-3'	for P_tet _with *AatII*
pTet R1	5'- tatctccttcttaaaAGATCTtttGAATTCttttctctatcactgatagggagtgg-3'	for P_tet _with *EcoRI *and *BglII*
pProS F1	5'- agatcaGACGTCttaattacccgactggtctttggcac -3'	for *Salmonela *based P_prpB _with *AatII*
pProS R2	5'- gggatatcagcctggaattTgatcatctggcgacc -3'	to remove *EcoRI *site
pProS F2	5'- ggtcgccagatgatcAaattccaggctgatatccc -3'	to remove *EcoRI *site
pProS R1	5'- tatctccttcttaaaAGATCTtttGAATTCcatgttagtaaattgttattcaag -3'	for *Salmonela *based P_prpB _with *EcoRI *and *BglII*
pProE F1	5'- agatcaGACGTCtcagcttttcagccgccgccagaac -3'	for *E. coli *based P_prpB _with *AatII*
pProE R2	5'- gtttcgcgatatcagcctTgagtttgatcacctgg -3'	to remove *XhoI *site
pProE F2	5'- ccaggtgatcaaactcAaggctgatatcgcgaaac -3'	to remove *XhoI *site
pProE R1	5'- tatctccttcttaaaAGATCTtttGAATTCttgttatcaacttgttatttgcgttg -3'	for *E. coli *based P_prpB _with *EcoRI *and *BglII*
lacUV5 F1	5'- agatcaGACGTCggtgcctaatgagtgagctaacttacattaattgc-3'	for P_lacUV5 _with *AatII*
PLlacO-1 F1	5'- agatcaGACGTCggtgcctaatgagtgagctaacttacattaattg-3'	for lacI with *AatII*
PLlacO-1 R2	5'- aatgtcaattgttatccgctcacaattctcgatcctctacgccggacg-3'	for lacI
PLlacO-1 F2	5'- cgtccggcgtagaggatcgagaattgtgagcggataacaattgacatt-3'	for P_LlacO-1_
PLlacO-1 R1	5'- tatctccttcttaaaAGATCTtttGAATTCggtcagtgcgtcctgctgatgtg-3'	for P_LlacO-1 _with *EcoRI *and *BglII*
pT7 F1	5'- agatcaGACGTCctcactgcccgctttccagtc-3'	for P_T7 _with *AatII*
pBAD F1	5'- agatcaGACGTCttatgacaacttgacggctacatcattcac-3'	for P_BAD _with *AatII*
pBAD R2	5'- gataaaaagcgtcaggtagAatccgctaatcttatgg-3'	to remove *BamHI *site
pBAD F2	5'-ccataagattagcggatTctacctgacgctttttatc-3'	to remove *BamHI *site
pBAD R1	5'-tatctccttcttaaaAGATCTtttGAATTCccaaaaaaacgggtatggagaaacag-3'	for P_BAD _with *EcoRI *and *BglII*

### Replication origins

The p15A origin was obtained from plasmid pZA31-luc, the ColE1 origin from plasmid pZE12-luc, and the pSC101* origin from plasmid pZS*24-MCS1 [39]. A *BglII *site in the pSC101* origin was eliminated by site-directed mutagenesis. The oligonucleotides used to remove the *BglII *site in the pSC101* origin were pSC101QC F1 and pSC101QC R1 creating pSC101**. Each origin of replication and terminator sequence module was cloned in using the *AvrII *and *SacI *sites. Plasmid pMBIS was used as template for the pBBR1 origin. The BBR1 region was amplified in two parts, and primers were designed to make a C to T point mutation in the overlapping region of the two PCR products to increase the copy number as reported [22]. Forward primer pBBR1 F1 (5'- gatcaCCTAGGctacagccgatagtctggaacagcgc -3') and reverse primer pBBR1 mut R1 (5'- ccggcaccgtgtTggcctacgtggtc -3') were used to generate the first product with a 5'-*AvrII *site, and forward primer pBBR1 mut F1 (5'- gaccacgtaggccAacacggtgccgg -3') and reverse primer pBBR1 R2 (5'- agatcaACTAGTgcctccggcctgcggcctgcgcgcttcg -3') were used to generate the second product with a 3'- *SpeI *site. These two parts were then combined in a splice overlap extension-PCR (SOE-PCR) reaction with primers pBBR1 F1 and pBBR1 R2 to create the product containing the entire pBBR1 origin of replication. The PCR product was digested with *AvrII *and *SpeI *and ligated with existing intermediate vectors to generate three additional intermediate vectors containing pBBR1 and each antibiotic resistance module.

### Antibiotic resistance

All antibiotic resistance segments (*SacI *to *AatII*) were digested from the parent plasmids listed in Table 1. The BglBrick restriction site found in Cm and Km resistance gene components were removed by site-specific mutagenesis. The oligonucleotides used to remove the *EcoRI *site in the Cm resistance gene were the forward CmQC F1 (5'-ctttcattgccatacgAaattccggatgagcattc-3') and reverse CmQC R1 (5'-gaatgctcatccggaattTcgtatggcaatgaaag-3') (point mutation is capitalized). The oligonucleotides used to remove the *BglII *site in the Km resistance gene promoter were KanQC F1 (5'- cctgtctcttgatcagatcAtgatcccctgc-3') and KanQC R1 (5'- gcaggggatcaTgatctgatcaagagacagg-3').

### Rfp (or gfp) and terminator

The rfp-terminator (rfp-term) module was constructed by splice overlap extension-PCR (SOE-PCR [47]. First, SOE-PCR was performed to generate *rfp *with BglBrick restriction sites *EcoRI *and *BglII *and RBS (TTTAAGAAGGAGATATACAT) on the 5'-end, and with BglBrick restriction sites *BamHI *and *XhoI *and a double terminator sequence followed by an *AatII *site on the 3'-end. Two PCRs were performed to amplify *rfp *and the terminator separately, using primers to introduce the restriction sites, RBS, and overlapping sequence for SOE-PCR. Forward primer RFP F1 and reverse primer RFP R1 were used to generate the product containing *EcoRI, BglII*, RBS, and *rfp*. Forward primer Term F1 and reverse primer Term R1 were used to generate the product containing the *BamHI, XhoI*, the double terminator sequence and *AvrII*. The products were then combined and a second PCR was performed with the RFP F1 and Term R1. The resulting SOE-PCR product (*rfp*-term) was in turn used in additional SOE-PCRs to generate complete modules containing the 8 different promoter systems followed by *rfp*-term.

### Promoters and repressors

The primers for each promoter system (containing repressor and promoter) were engineered to include a 5'*AatII *site for later cloning steps and an *rfp *overlapping sequence on the 3' end to facilitate the addition of the *rfp*-terminator module via SOE-PCR. When the promoter system contained any of the 4 BglBrick restriction sites, an additional set of primers to remove the restriction site was prepared for SOE-PCR. Primers for each promoter system are listed in the Table 2.

### Final pBb vector assembly

To construct the promoter system with the *rfp*-terminator module, each of the eight promoter system modules were combined with *rfp*-terminator by SOE-PCR using the F1 primer from each promoter system construction and the reverse primer Term R1. These eight products were then digested with *AatII *and *AvrII *and individually ligated with the *AatII *and *AvrII *digested fragment from the intermediate plasmid containing *amp*^R ^and ColE1. The eleven remaining intermediate plasmids were then digested with *AvrII *and *AatII *to isolate the antibiotic resistance-replication origin (AR-ori) modules. In total, each of the twelve AR-ori modules was ligated with each of the eight *AvrII *and *AatII *digested promoter-rfp-terminator modules to produce 96 unique pBb vectors.

### Data sheet experiments

#### General

Ampicillin-resistant pBb plasmids were transformed into *E. coli *BLR(DE3) electrocompetent cells and/or *E. coli *DH1 electrocompetent cells and plated on LB-agar with 50 μg/ml Carbenicillin (Cb) for overnight incubation at 37°C. A single colony was picked and used to prepare the seed culture in LB broth containing 50 μg/ml Cb. Fresh culture tubes with 3 ml LB broth containing 50 μg/ml Cb were inoculated with 60 μl overnight seed culture and grown at 37°C, 200 rpm until the OD_600 _reached about 0.55. All experiments were replicated in triplicate.

#### Inducer dose response

The outer wells of a 96-well clear-bottom plate with lid (Corning no: 3631) were filled with 200 μl sterile water and the plate was sterilized by using the *optimal crosslink *setting on the UV crosslinker (Spectronics, Corp.). 10 × serial dilutions were made of inducers appropriate for each plasmid being tested and 20 μl was pipetted into each well so that the final volume of 200 μl would give 1x inducer concentration. Each plate included 3 control wells containing pBbE5a-RFP (or GFP) in BLR(DE3) induced with 12.5 μM IPTG. Appropriate volumes of culture and LB/Cb were added to the 96-well plate with lid and grown in a Safire (Tecan) microplate reader at 30°C for 20.5 hours. OD_600 _and RFP fluorescence were measured every 570 seconds using an excitation wavelength of 584 nm and an emission wavelength of 607 nm. For the constructs containing GFP (pBbB plasmids), an excitation wavelength of 400 nm and an emission wavelength of 510 nm were used for fluorescence measurement.

#### Strain and medium dependence

*E. coli *BLR(DE3) and DH1 transformed with pBb plasmid were streaked on LB-agar with 50 μg/ml Cb and grown at 37°C overnight. Seed cultures were prepared in LB broth containing 50 μg/mL Cb inoculated with a single colony and grown at 37°C, 200 rpm overnight. Each experiment with a pBb plasmid-harboring strain was replicated in triplicate, and each set of experiments included 6 control tubes containing pBbE5a-RFP in BLR(DE3) in LB (3 uninduced and 3 induced with 100 μM IPTG). For the M9 minimal medium (MM) experiment, three rounds of adaptation were performed in minimal medium. After adaptation, fresh tubes with 3 mL fresh MM were inoculated with adapted seed culture to OD_600 _approximately 0.15 and grown at 37°C to OD_600 _of approximately 0.5. One set of tubes were induced at different inducer concentrations and all cultures were grown at 30°C, 200 rpm for 66 hours post induction. Samples were taken at 18 h, 42 h and 66 h post induction. 25 μL of culture was taken into a 96-well plate and diluted to 200 μL with fresh medium, and OD_600 _and fluorescence were measured. For LB and TB media experiments, overnight seed cultures were used directly for inoculation without adaptation.

#### Catabolite repression and inducer crosstalk

Seed cultures were prepared as described in strain and medium dependence experiments. Three different media (MM, phosphate buffered LB, and phosphate buffered TB) containing 1% glucose were used for catabolite repression experiments. Inoculated cultures were grown at 37°C to OD_600 _of approximately 0.5, and induced to achieve maximum expression (100 μM IPTG, 20 mM arabinose, 400 nM aTc, or 20 mM propionate). Cultures were grown at 30°C, 200 rpm for 66 hours post induction, and OD_600 _and fluorescence was measured at each sampling. For the inducer crosstalk experiment, LB broth containing 50 μg/ml Cb was inoculated with seed cultures containing *E. coli *BLR(DE3) harboring the ampicillin-resistant pBb. Cultures were induced at OD_600 _of approximately 0.5 with the appropriate inducer, and one of the non-cognate inducers was also added to the individually induced culture during induction. Cultures were grown at 30°C, 200 rpm for 18 hours post-induction, and OD_600 _and fluorescence were measured using the Tecan.

#### Bacterial DNA isolation to quantify plasmid copy number

*E. coli *DH1 and BLR were grown overnight at 30°C, 200 rpm shaking after inoculating 5 mL cultures of LB medium (supplemented with 50 μg/mL kanamycin) with single colonies from freshly streaked plates. After sub-culturing (1:50) into shake flasks containing 50 mL of LB medium (supplemented with 50 μg/mL kanamycin), cells were grown at 30°C, 200 rpm shaking until an OD_600 _of 0.3-0.4 was reached. At this time, 1 mL of cells was spun down and the supernatant subsequently removed. The cell pellets were then frozen. Total DNA was isolated from these pellets for use at a future date. The DNA isolation method reported in previous publications [33,48] was adopted. Bacterial cell pellets were resuspended in 400 μL of 50 mM Tris/50 mM EDTA, pH 8, by vortexing. Cell membranes were permeablized by the addition of 8 μL of 50 mg/mL lysozyme (Sigma) in 10 mM Tris/1 mM EDTA, pH 8, followed by incubation at 37°C for 30 min. To complete cell lysis, 4 μL of 10% SDS and 8 μL of 20 mg/mL Proteinase K solution (Invitrogen) were added to each tube, mixed with a syringe with 21-gauge, 1.5-inch needle, and incubated at 50°C for 30 min. Proteinase K was subsequently heat inactivated at 75°C for 10 min, and RNA was digested with the addition of 2 μL of 100 mg/mL RNase A solution (Qiagen) followed by incubation at 37°C for 30 min. Total DNA extraction then proceeded by adding 425 μL of 25:24:1 phenol:chloroform:isoamyl alcohol, vortexing vigorously for ~1 min, allowing the tubes to sit at room temperature for a few minutes, and then centrifugation for 5 min at 14,000 × g, 4°C. Next, 300 μL of the upper aqueous phase was transferred to a new tube using a wide-opening pipet tip. DNA extraction continued by adding 400 μL of chloroform to each tube, vigorous vortexing for ~1 min, allowing the tubes to sit at room temperature for a few minutes, and centrifugation for 5 min at 14,000 × g, 4°C. Next, 200 μL of the upper aqueous phase was transferred to a new tube using a wide-opening pipet tip. Following chloroform extraction, total DNA was ethanol precipitated overnight, washed with 70% ethanol, and finally resuspended in 40 μL of nuclease-free water. DNA concentration and purity were assayed using a Nanodrop spectrophotometer, and integrity examined on 1% agarose gels.

#### Real-time qPCR quantification of plasmid copy number

Primer sets specific to the neomycin phosphotransferase II (*nptII*) gene (forward: GCGTTGGCTACCCGTGATAT, reverse: AGGAAGCGGTCAGCCCAT) [49] and 16S rDNA gene (forward: CCGGATTGGAGTCTGCAACT, reverse: GTGGCATTCTGATCCACGATTAC) [33] were used for real-time qPCR. These primers amplified a single product of the expected size as confirmed by the melting temperatures of the amplicons. *nptII *resides in single-copy on the plasmids characterized in this study, while 16S rDNA gene resides on multiple copies on the *E. coli *chromosome [36] and was used for normalization [22,33,35]. In order to determine plasmid copy number (i.e. number of plasmids per genomic equivalent), *E. coli *DH1 and BLR transgenic strains with a single *nptII *integration (data not shown) were used for calibration. Total DNA isolated from each strain was first digested overnight using *EcoR*I (New England Biolabs) at 37°C. Real-time qPCR was conducted on a BioRad iCycler with 96-well reaction blocks in the presence of SYBR Green under the following conditions: 1X iQ SYBR Green Supermix (BioRad), 150 nM *nptII *(500 nM 16S) primers in a 25 μL reaction. Real-time qPCR cycling was 95°C for 3 min, followed by 40 cycles of 30 sec at 95°C, 30 sec at 60°C, and 30 sec at 72°C. Threshold cycles (Ct) were determined with iCycler (BioRad) software for all samples. A standard curve was prepared for quantification. For this purpose, a four-fold dilution series of a total of seven dilutions was prepared from a digested total DNA sample, and each dilution was subjected to qPCR analysis in at least duplicate with either the *nptII*- or 16S-specific primers. Obtained Ct values were used by the iCycler software package to plot a standard curve that allowed quantification of *nptII *or 16S in the digested total DNA samples (i.e. unknowns) relative to the DNA sample used to prepare the standard curve.

#### Expression control in the three-plasmid system

BLR (DE3) cells were transformed with three plasmids: pBbA8a-CFP, pBbE5c-YFP and pBbS2k-RFP. A single colony was used to inoculate LB medium and the overnight cultures were grown at 37°C in minimal medium (M9 medium supplied with 75 mM MOPS, 2 mM MgSO_4_, 1 mg/L thiamine, 10 nM FeSO_4_, 0.1 mM CaCl_2 _and micronutrients) supplemented with 2% glucose. Cells were induced at OD ~0.6 with combinations of different amounts of arabinose, IPTG and aTc. In detail, the arabinose concentrations used were 0, 5 mM, and 20 mM; the IPTG concentrations used were 0, 30 μM, and 100 μM; and the aTc concentrations used were 0, 12.5 nM, 25 nM, and 40 nM. After induction, cells were grown at 30°C for 12 hours until cell culture fluorescence was measured. Cell culture fluorescence was recorded on a SpectraMax M2 plate reader (Molecular Devices) using 96-well Costar plates with each well containing 150 μl of cell culture. For CFP, λ_ex _= 433 nm and λ_em _= 474 nm were used; for YFP, λ_ex _= 500 nm and λ_em _= 530 nm were used; and for RFP, λ_ex _= 584 nm and λ_em _= 615 nm were used. Cell density was estimated by measuring the absorbance at 610 nm. Cell culture fluorescence from each well was normalized by its cell density. All the data were average from at least two independent measurements.

## List of abbreviation used

BBF: BioBricks Foundation; RFC: request for comments; RBS: ribosomal binding site; SOE: splice overlap extension; PCR: polymerase chain reaction; RFP: red fluorescent protein; GFP: green fluorescent protein; CFP: cyan fluorescent protein; YFP: yellow fluorescent protein; CCR: carbon catabolite repression; aTc: anhydrotetracycline; IPTG: isopropyl-β-D-thiogalactoside; Amp: ampicillin; Km: kanamycin; Cm: chloramphenicol; Cb: carbenicillin; MM: minimal media; LB: Luria-Bertani; TB: Terrific broth; OD: optical density; qPCR: quantitative PCR

## Declaration of competing interests

The authors declare that they have no competing interests.

## Authors' contributions

The BglBrick vectors were designed by TSL, SKL, and JDK. The datasheet was designed by TSL, RK, and JDK. The vectors were constructed by RK and WJH, and initial datasheet experiment was performed by RK. Three-plasmid experiments were performed by FZ and NP, and the copy number measurement was performed by MH. The manuscript was drafted by TSL, RK, FZ, MH, WJH, and JDK. All authors read and approved the final manuscript.

## Supplementary Material

Additional file 1 **Datasheets for 32 BglBrick vectors**. PDF file of the datasheets for 32 BglBrick vectors. Click here for file

Additional file 2 **Method for the preparation of promoter-less BglBrick vectors**. MS Word file with experimental details for the preparation of 12 promoter-less BglBrick vectors. Click here for file
