# Supplementary material for: BglBrick vectors and datasheets: A synthetic biology platform for gene expression
Source: J Biol Eng. 2011 Sep 20;5:12. doi: 10.1186/1754-1611-5-12 (PMC3189095; doi:10.1186/1754-1611-5-12)
Supplement: Additional file 1 — Datasheets for 32 BglBrick vectors. PDF file of the datasheets for 32 BglBrick vectors. [file 1754-1611-5-12-S1.PDF]

pBbA1

IPTG inducible promoter system

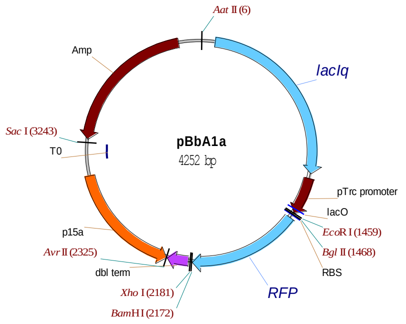

Constructs available	Freezer location (-80)
pBbA1a-RFP	2478
pBbA1k-RFP	2484
pBbA1c-RFP	2491

A = p15A ori (8-10 copies per cell) 1 = pTrc
experiments represented on this datasheet were performed using pBbA1a-RFP
pBbE5a-RFP in BLR(DE3) in LB induced (100mM IPTG) was used as control

INDUCER DOSE RESPONSE

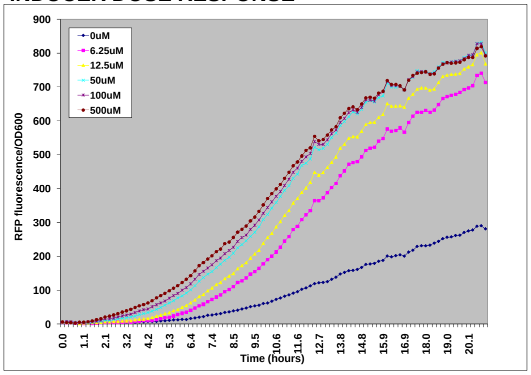

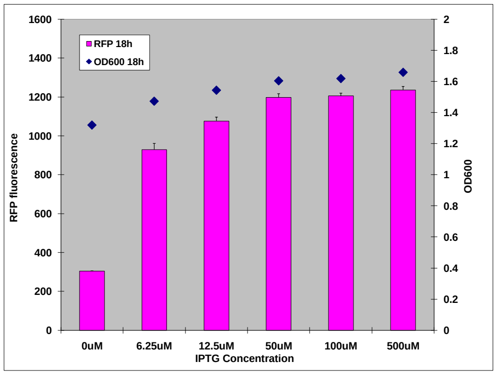

BLR(DE3) in LB, 30°C

STRAIN and MEDIA DEPENDENCE

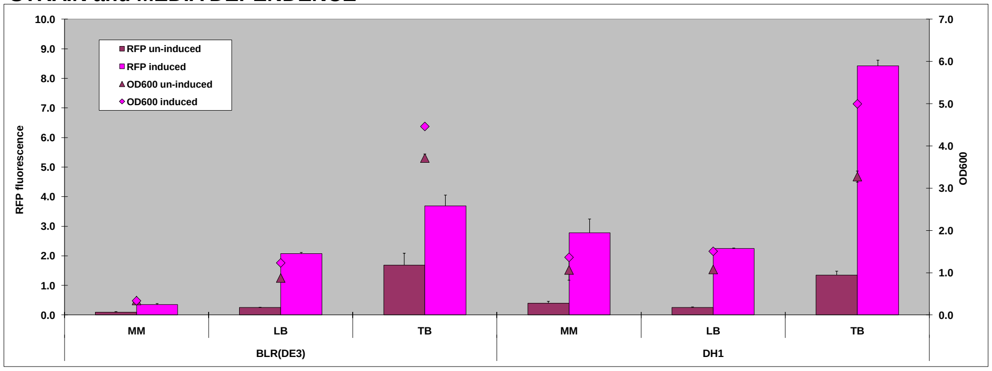

3ml cultures grown in test tubes, induced with 100uM IPTG, grown at 30°C post-induction, measurements taken in Tecan 18h post-induction
MM media is supplemented with 0.5% glucose, TB media is supplemented with 2% glycerol
RFP and OD normalized to pBbE5a-RFP in BLR(DE3) in LB induced (100uM IPTG)

CATABOLITE REPRESSION

RFP/OD600 in BLR(DE3) as a percentage of induced without glucose, 18h post-induction

	LB	LB*+1%glucose	MM	MM+1%glucose	TB	TB*+1%glucose
pBbA1a induced	100.0% (+/-2.3)	67.5% (+/-2.3)	100.0% (+/-24.6)	40.9% (+/-12.1)	100.0% (+/-2.6)	86.2% (+/-6.0)
pBbA1a un-induced	14.3% (+/-0.2)	3.4% (+/-1.0)	24.6% (+/-0.0)	12.1% (+/-0.0)	83.3% (+/-31.3)	14.2% (+/-1.8)

*100mM potassium phosphate buffered, pH 7.5

CROSSTALK

RFP/OD600 in BLR(DE3) in LB, 18h post-induction, pBbE1a construct

	IPTG(100uM)	IPTG(100uM) +aTc(400nM)	IPTG(100uM) +Arabinose(20mM)	IPTG(100uM) +Propionate(20mM)	Un-induced
pTrc	100.0% (+/-2.6)	112.0% (+/-5.5)	103.3% (+/-0.6)	100.3% (+/-3.7)	25.4% (+/-1.5)

pBbB1

IPTG inducible promoter system

Constructs available	Freezer location (-80)
pBbB1a-GFP	2629
pBbB1k-GFP	2637
pBbB1c-GFP	2645

B = BBR1 ori (17-20 copies per cell) 1 = pTrc
experiments represented on this datasheet were performed using pBbB1a-GFP
pBbE5a-GFP in BLR(DE3) in LB induced (100mM IPTG) was used as control

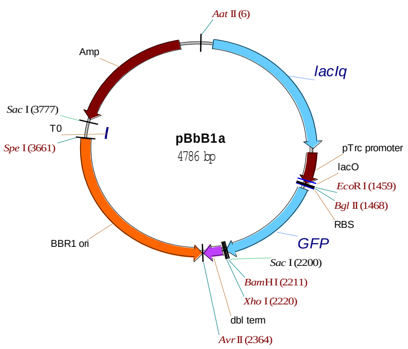

INDUCER DOSE RESPONSE

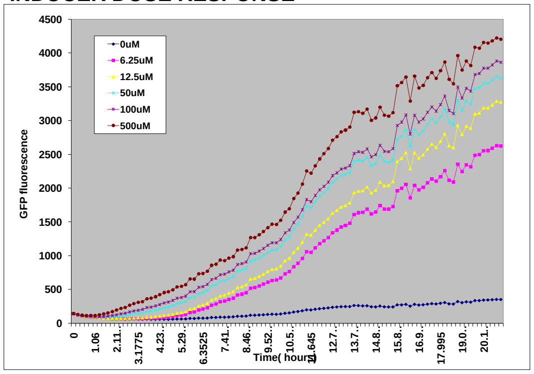

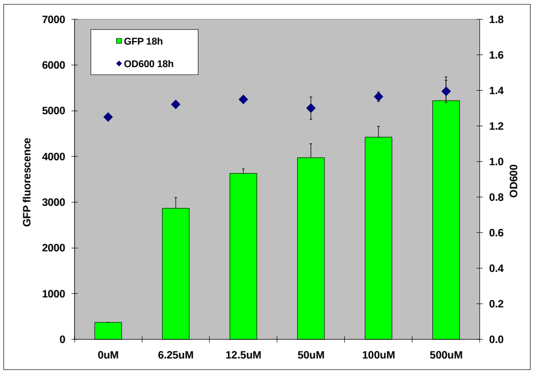

BLR(DE3) in LB, 30°C

STRAIN and MEDIA DEPENDENCE

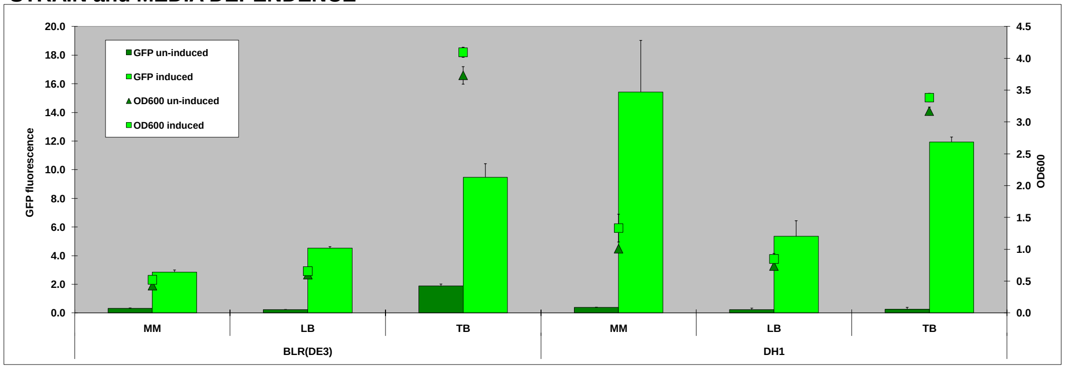

3ml cultures grown in test tubes, induced with 100uM IPTG, grown at 30°C post-induction, measurements taken in Tecan 18h post-induction
MM media is supplemented with 0.5% glucose, TB media is supplemented with 2% glycerol
GFP and OD normalized to pBbE5a-GFP in BLR(DE3) in LB induced (100uM IPTG)

CATABOLITE REPRESSION

RFP/OD600 in BLR(DE3) as a percentage of induced without glucose, 18h post-induction

	LB	LB*+1%glucose	MM	MM+1%glucose	TB	TB*+1%glucose
pBbB1a induced	100.0% (+/-4.1)	74.7% (+/-1.7)	100.0% (+/-9.9)	122.0% (+/-8.2)	100.0% (+/-8.4)	200.9% (+/-9.6)
pBbB1a un-induced	5.5% (+/-0.4)	3.2% (0.2)	13.3% (+/-1.0	13.1% (1.3)	21.7% (+/-1.0	18.2% (3.4)

*100mM potassium phosphate buffered, pH 7.5

CROSSTALK

RFP/OD600 in BLR(DE3) in LB, 18h post-induction, pBbE1a construct

	IPTG(100uM)	IPTG(100uM) +aTc(400nM)	IPTG(100uM) +Arabinose(20mM)	IPTG(100uM) +Propionate(20mM)	Un-induced
pTrc	100.0% (+/-2.6)	112.0% (+/-5.5)	103.3% (+/-0.6)	100.3% (+/-3.7)	25.4% (+/-1.5)

pBbE1

IPTG inducible promoter system

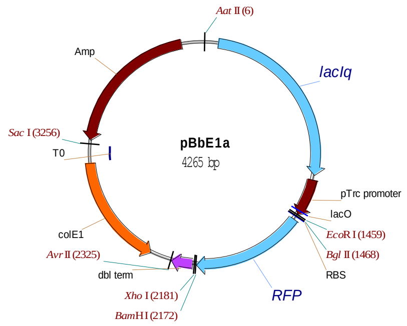

Constructs available	Freezer location (-80)
pBbE1a-RFP	2469
pBbE1k-RFP	2497
pBbE1c-RFP	2502

E = colE1 ori (20-30 copies per cell) 1 = pTrc
experiments represented on this datasheet were performed using pBbE1a-RFP
pBbE5a-RFP in BLR(DE3) in LB induced (100mM IPTG) was used as control

INDUCER DOSE RESPONSE

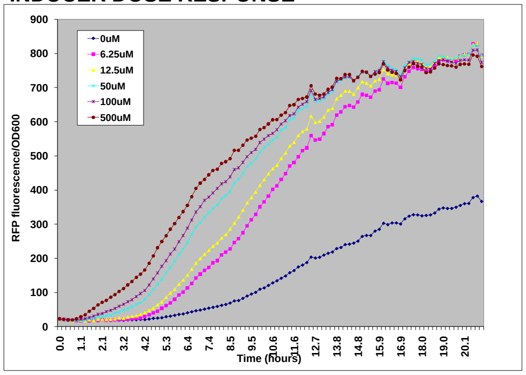

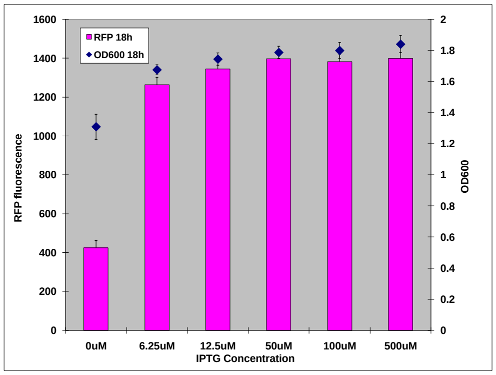

BLR(DE3) in LB, 30°C

STRAIN and MEDIA DEPENDENCE

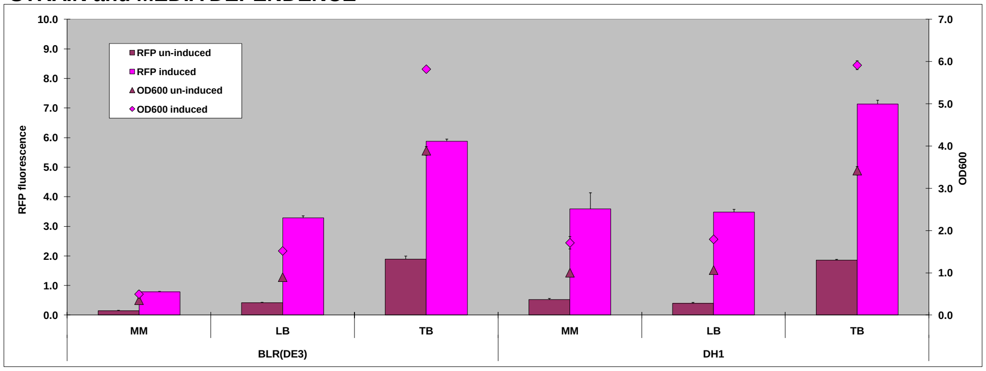

3ml cultures grown in test tubes, induced with 100uM IPTG, grown at 30°C post-induction, measurements taken in Tecan 18h post-induction
MM media is supplemented with 0.5% glucose, TB media is supplemented with 2% glycerol
RFP and OD normalized to pBbE5a-RFP in BLR(DE3) in LB induced (100uM IPTG)

CATABOLITE REPRESSION

RFP/OD600 in BLR(DE3) as a percentage of induced without glucose, 18h post-induction

	LB	LB*+1%glucose	MM	MM+1%glucose	TB	TB*+1%glucose
pBbE1a induced	100.0% (+/-0.8)	82.2% (+/-1.9)	100.0% (+/-25.4)	64.2% (+/-12.1)	100.0% (+/-2.8)	78.5% (+/-1.0)
pBbE1a un-induced	18.1% (+/-1.2)	4.8% (+/-0.2)	25.4% (+/-0.0)	12.1% (+/-0.0)	54.4% (+/-1.6)	11.7% (+/-0.8)

*100mM potassium phosphate buffered, pH 7.5

CROSSTALK

RFP/OD600 in BLR(DE3) in LB, 18h post-induction, pBbE1a construct

	IPTG(100uM)	IPTG(100uM) +aTc(400nM)	IPTG(100uM) +Arabinose(20mM)	IPTG(100uM) +Propionate(20mM)	Un-induced
pTrc	100.0% (+/-2.6)	112.0% (+/-5.5)	103.3% (+/-0.6)	100.3% (+/-3.7)	25.4% (+/-1.5)

pBbS1

IPTG inducible promoter system

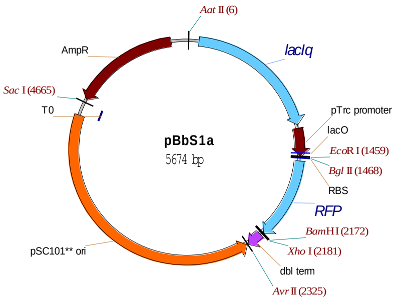

Constructs available	Freezer location (-80)
pBbS1a-RFP	2548
pBbS1k-RFP	2556
pBbS1c-RFP	2564

S = SC101 ori (4-6 copies per cell) 1 = pTrc
experiments represented on this datasheet were performed using pBbS1a-RFP
pBbE5a-RFP in BLR(DE3) in LB induced (100mM IPTG) was used as control

INDUCER DOSE RESPONSE

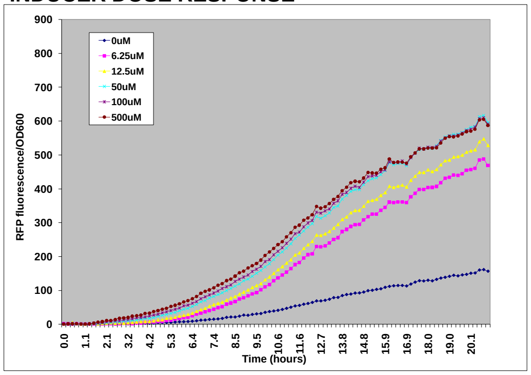

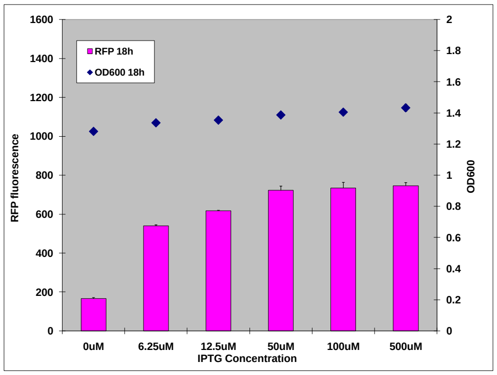

BLR(DE3) in LB, 30°C

STRAIN and MEDIA DEPENDENCE

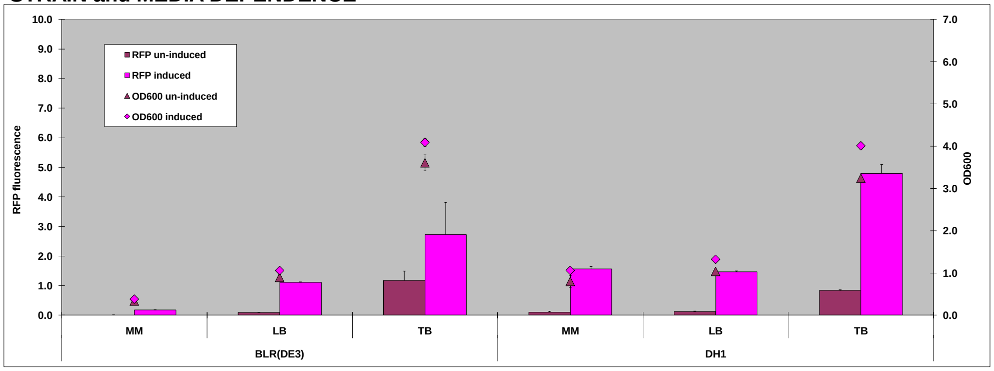

3ml cultures grown in test tubes, induced with 100uM IPTG, grown at 30°C post-induction, measurements taken in Tecan 18h post-induction
MM media is supplemented with 0.5% glucose, TB media is supplemented with 2% glycerol
RFP and OD normalized to pBbE5a-RFP in BLR(DE3) in LB induced (100uM IPTG)

CATABOLITE REPRESSION

RFP/OD600 in BLR(DE3) as a percentage of induced without glucose, 18h post-induction

	LB	LB*+1%glucose	MM	MM+1%glucose	TB	TB*+1%glucose
pBbS1a induced	100.0% (+/-1.9)	59.2% (+/-12.3)	100.0% (+/-3.1)	45.4% (+/-1.0)	100.0% (+/-10.8)	58.3% (+/-7.2)
pBbS1a un-induced	6.2% (0.5)	1.5% (+/-0.4)	3.1% (+/-0.0)	1.0% (+/-0.0)	63.0% (18.5)	11.8% (+/-1.5)

*100mM potassium phosphate buffered, pH 7.5

CROSSTALK

RFP/OD600 in BLR(DE3) in LB, 18h post-induction, pBbE1a construct

	IPTG(100uM)	IPTG(100uM) +aTc(400nM)	IPTG(100uM) +Arabinose(20mM)	IPTG(100uM) +Propionate(20mM)	Un-induced
pTrc	100.0% (+/-2.6)	112.0% (+/-5.5)	103.3% (+/-0.6)	100.3% (+/-3.7)	25.4% (+/-1.5)

pBbA2

Anhydrotetracycline inducible promoter system

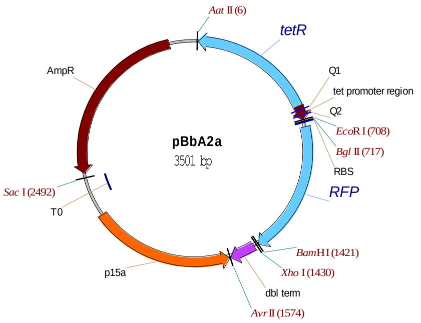

Constructs available	Freezer location (-80)
pBbA2a-RFP	2479
pBbA2k-RFP	2485
pBbA2c-RFP	2492

A = p15A ori (8-10 copies per cell) 2 = pTet
experiments represented on this datasheet were performed using pBbA2a-RFP
pBbE5a-RFP in BLR(DE3) in LB induced (100mM IPTG) was used as control

INDUCER DOSE RESPONSE

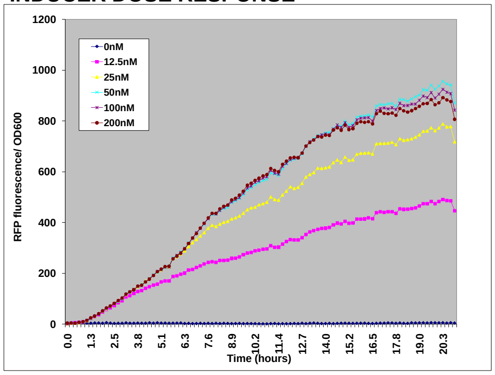

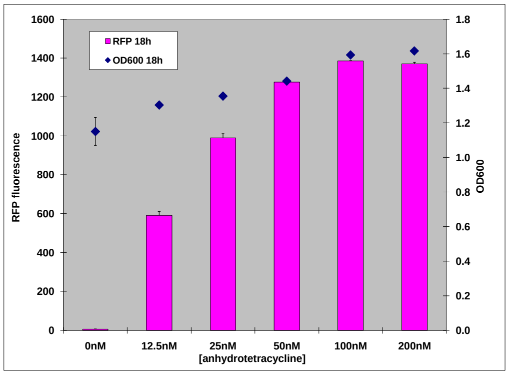

BLR(DE3) in LB, 30°C

STRAIN and MEDIA DEPENDENCE

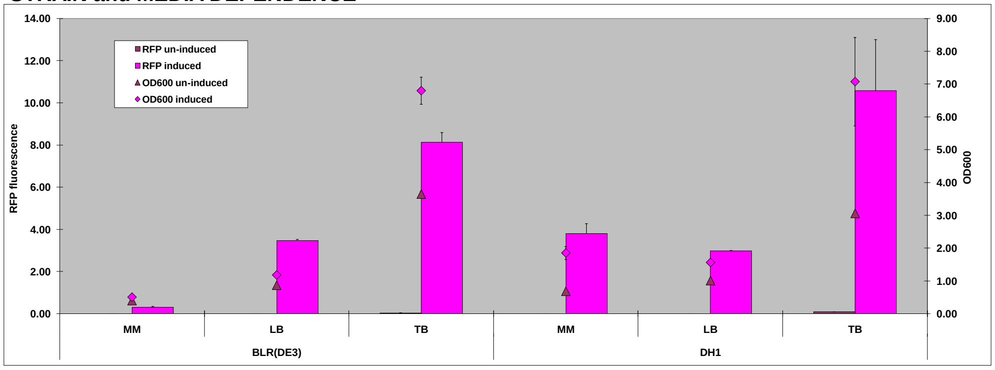

3ml cultures grown in test tubes, induced with 400nM anhydrotetracycline (aTc), grown at 30°C post-induction, measurements taken in Tecan 18h post-induction
MM media is supplemented with 0.5% glucose, TB media is supplemented with 2% glycerol
RFP and OD normalized to pBbE5a-RFP in BLR(DE3) in LB induced (100uM IPTG)

CATABOLITE REPRESSION

RFP/OD600 in BLR(DE3) as a percentage of induced without glucose, 18h post-induction

	LB	LB*+1%glucose	MM	MM+1%glucose	TB	TB*+1%glucose
pBbA2a induced	100.0% (+/-3.0)	85.7% (+/-4.3)	100.0% (+/-22.3)	111.1% (+/-12.8)	100.0% (+/-4.7)	101.5% (+/-2.9)
pBbA2a un-induced	0.0% (+/-0.0)	0.0% (+/-0.0)	0.0% (+/-0.0)	0.0% (+/-0.0)	0.7% (+/-0.2)	0.7% (+/-0.3)

*100mM potassium phosphate buffered, pH 7.5

CROSSTALK

RFP/OD600 in BLR(DE3) in LB, 18h post-induction, pBbE2a construct

	aTc(400nM)	aTc(400nM) +IPTG(100uM)	aTc(400nM) +Arabinose(20mM)	aTc(400nM) +Propionate(20mM)	Un-induced
pTet	100.0% (+/-4.3)	101.0% (+/-1.3)	86.6% (+/-0.8)	91.3% (+/-1.7)	0.0% (+/-0.0)

pBbB2

Anhydrotetracycline inducible promoter system

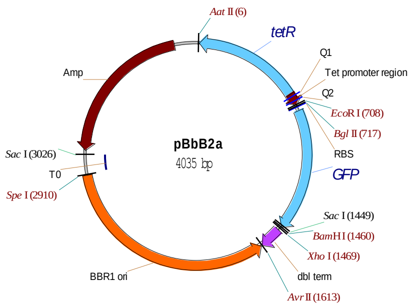

Constructs available	Freezer location (-80)
pBbB2a-GFP	2630
pBbB2k-GFP	2638
pBbB2c-GFP	2646

B = BBR1 ori (17-20 copies per cell) 2 = pTet
experiments represented on this datasheet were performed using pBbB2a-GFP
pBbE5a-GFP in BLR(DE3) in LB induced (100mM IPTG) was used as control

INDUCER DOSE RESPONSE

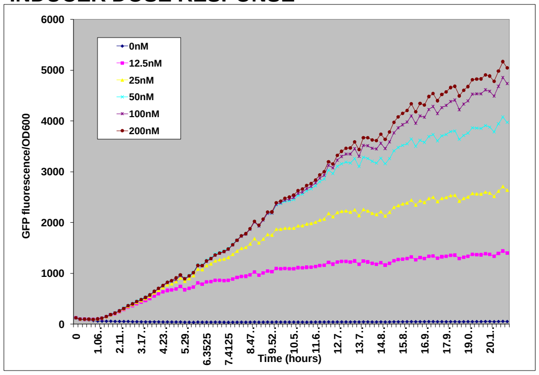

BLR(DE3) in LB, 30°C

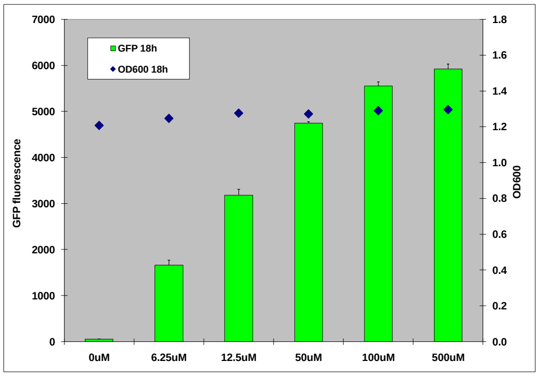

STRAIN and MEDIA DEPENDENCE

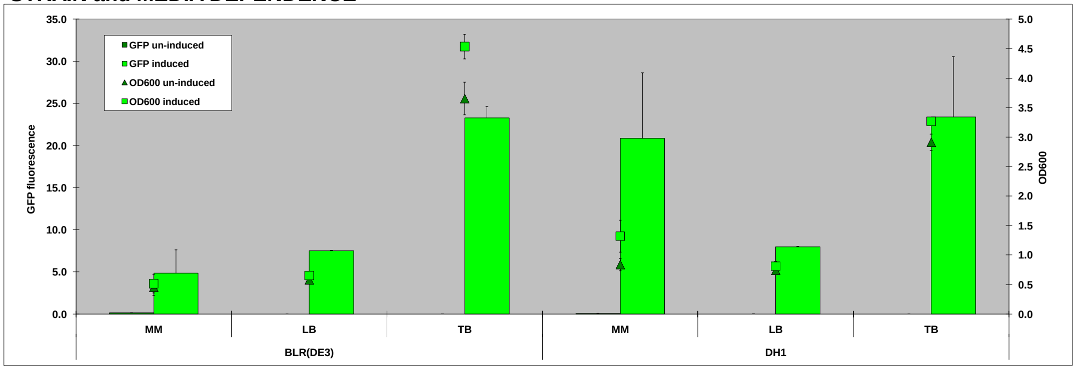

3ml cultures grown in test tubes, induced with 400nM anhydrotetracycline (aTc), grown at 30°C post-induction, measurements taken in Tecan 18h post-induction
MM media is supplemented with 0.5% glucose, TB media is supplemented with 2% glycerol
GFP and OD normalized to pBbE5a-GFP in BLR(DE3) in LB induced (100uM IPTG)

CATABOLITE REPRESSION

RFP/OD600 in BLR(DE3) as a percentage of induced without glucose, 18h post-induction

	LB	LB*+1%glucose	MM	MM+1%glucose	TB	TB*+1%glucose
pBbB2a induced	100.0% (+/-1.6)	134.4% (+/-16.0)	100.0% (+/-24.3)	129.7% (+/-27.3)	100.0% (+/-2.1)	181.0% (+/-20.1)
pBbB2a un-induced	0.0% (+/-0.0)	1.4% (+/-0.0)	3.3% (+/-0.3)	2.4% (+/-0.5)	0.0% (+/-0.0)	0.4% (+/-0.3)

*100mM potassium phosphate buffered, pH 7.5

CROSSTALK

RFP/OD600 in BLR(DE3) in LB, 18h post-induction, pBbE2a construct

	aTc(400nM)	aTc(400nM) +IPTG(100uM)	aTc(400nM) +Arabinose(20mM)	aTc(400nM) +Propionate(20mM)	Un-induced
pTet	100.0% (+/-4.3)	101.0% (+/-1.3)	86.6% (+/-0.8)	91.3% (+/-1.7)	0.0% (+/-0.0)

pBbE2

Anhydrotetracycline inducible promoter system

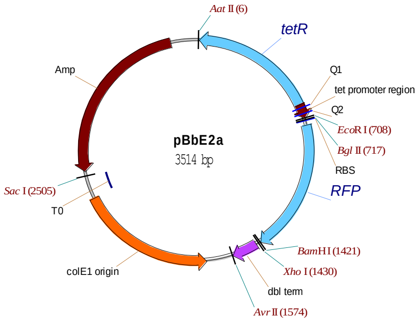

Constructs available	Freezer location (-80)
pBbE2a-RFP	2471
pBbE2k-RFP	2498
pBbE2cRFP	2501

E = colE1 ori (20-30 copies per cell) 2 = pTet
experiments represented on this datasheet were performed using pBbE2a-RFP
pBbE5a-RFP in BLR(DE3) in LB induced (100mM IPTG) was used as control

INDUCER DOSE RESPONSE

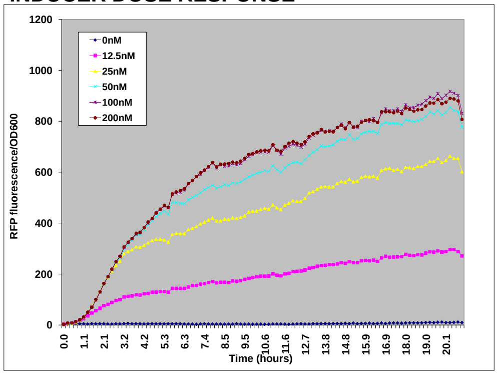

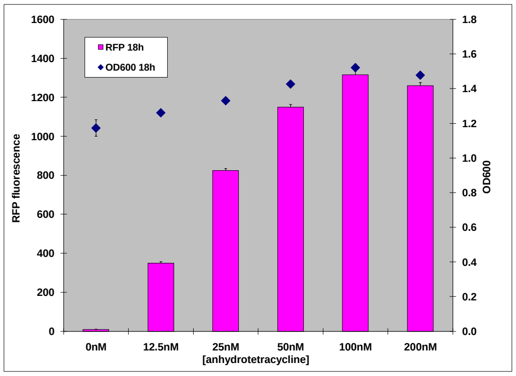

BLR(DE3) in LB, 30°C

STRAIN and MEDIA DEPENDENCE

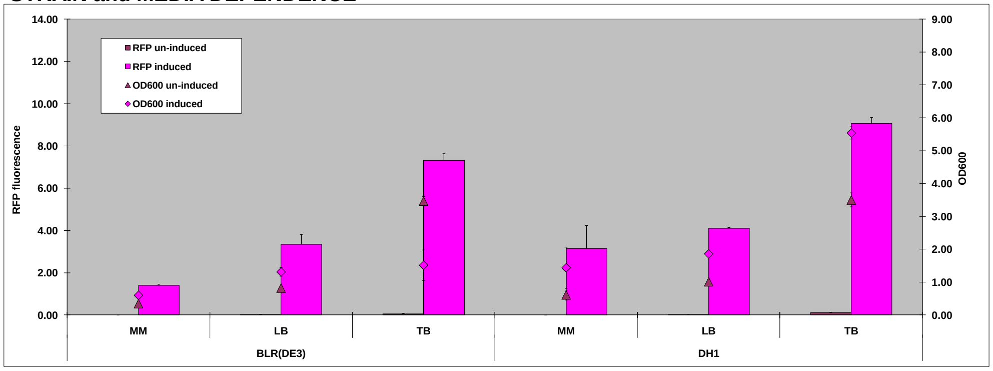

3ml cultures grown in test tubes, induced with 400nM anhydrotetracycline (aTc), grown at 30°C post-induction, measurements taken in Tecan 18h post-induction
MM media is supplemented with 0.5% glucose, TB media is supplemented with 2% glycerol
RFP and OD normalized to pBbE5a-RFP in BLR(DE3) in LB induced (100uM IPTG)

CATABOLITE REPRESSION

RFP/OD600 in BLR(DE3) as a percentage of induced without glucose, 18h post-induction

	LB	LB*+1%glucose	MM	MM+1%glucose	TB	TB*+1%glucose
pBbE2a induced	100.0% (+/- 1.5)	79.5% (+/-2.4)	100.0% (+/- 4.7)	107.0% (+/-14.9)	100.0% (+/- 27.3)	110.7% (+/-5.9)
pBbE2a un-induced	0.0% (+/- 0.0)	0.2% (+/-0.3)	0.0% (+/- 0.0)	0.0% (+/-0.0)	0.3% (+/- 0.2)	0.2% (+/-0.1)

*100mM potassium phosphate buffered, pH 7.5

CROSSTALK

RFP/OD600 in BLR(DE3) in LB, 18h post-induction, pBbE2a construct

	aTc(400nM)	aTc(400nM) +IPTG(100uM)	aTc(400nM) +Arabinose(20mM)	aTc(400nM) +Propionate(20mM)	Un-induced
pTet	100.0% (+/-4.3)	101.0% (+/-1.3)	86.6% (+/-0.8)	91.3% (+/-1.7)	0.0% (+/-0.0)

pBbS2

Anhydrotetracycline inducible promoter system

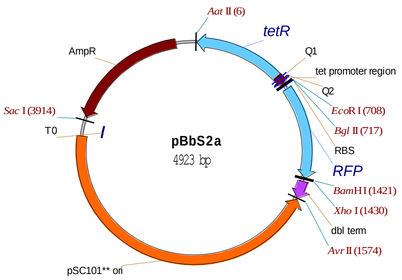

Constructs available	Freezer location (-80)
pBbS2a-RFP	2549
pBbS2k-RFP	2557
pBbS2c-RFP	2565

S = SC101 ori (4-6 copies per cell) 2 = pTet
experiments represented on this datasheet were performed using pBbS2a-RFP
pBbE5a-RFP in BLR(DE3) in LB induced (100mM IPTG) was used as control

INDUCER DOSE RESPONSE

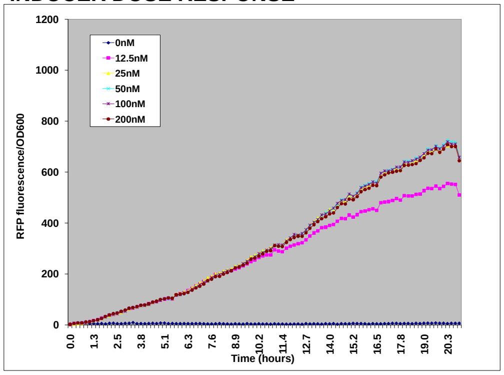

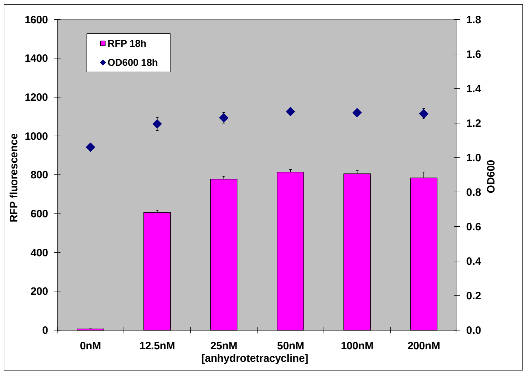

BLR(DE3) in LB, 30°C

STRAIN and MEDIA DEPENDENCE

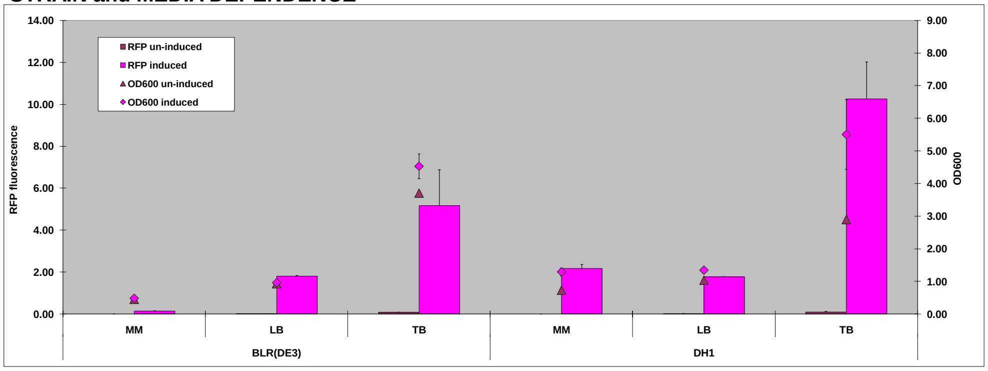

3ml cultures grown in test tubes, induced with 400nM anhydrotetracycline (aTc), grown at 30°C post-induction, measurements taken in Tecan 18h post-induction
MM media is supplemented with 0.5% glucose, TB media is supplemented with 2% glycerol
RFP and OD normalized to pBbE5a-RFP in BLR(DE3) in LB induced (100uM IPTG)

CATABOLITE REPRESSION

RFP/OD600 in BLR(DE3) as a percentage of induced without glucose, 18h post-induction

	LB	LB*+1%glucose	MM	MM+1%glucose	TB	TB*+1%glucose
pBbS2a induced	100. % (+/-1.3)	70.7% (+/-1.7)	100.0% (+/-11.9)	106.3% (+/-25.9)	100.0% (+/-26.8)	83.9% (+/-3.7)
pBbS2a un-induced	0.0% (+/-0.0)	0.5% (0.5)	0.0% (+/-0.0)	0.0% (0.0)	1.9% (+/-0.3)	1.1% (0.6)

*100mM potassium phosphate buffered, pH 7.5

CROSSTALK

RFP/OD600 in BLR(DE3) in LB, 18h post-induction, pBbE2a construct

	aTc(400nM)	aTc(400nM) +IPTG(100uM)	aTc(400nM) +Arabinose(20mM)	aTc(400nM) +Propionate(20mM)	Un-induced
pTet	100.0% (+/-4.3)	101.0% (+/-1.3)	86.6% (+/-0.8)	91.3% (+/-1.7)	0.0% (+/-0.0)

pBbA3

Propionate inducible promoter system

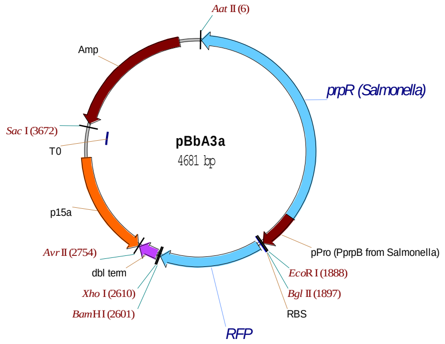

Constructs available	Freezer location (-80)
pBbA3a-RFP	2508
pBbA3k-RFP	2509
pBbA3c-RFP	2510

A = p15A ori (8-10 copies per cell) 3 = pProS
experiments represented on this datasheet were performed using pBbA3a-RFP
pBbE5a-RFP in BLR(DE3) in LB induced (100mM IPTG) was used as control

INDUCER DOSE RESPONSE

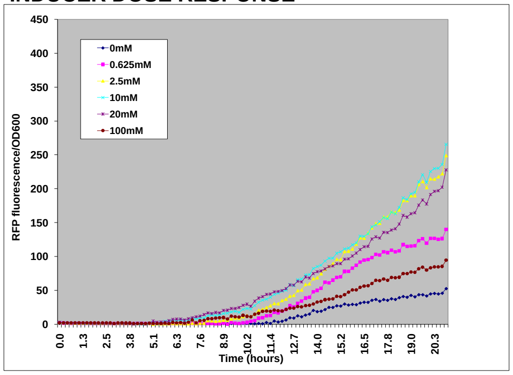

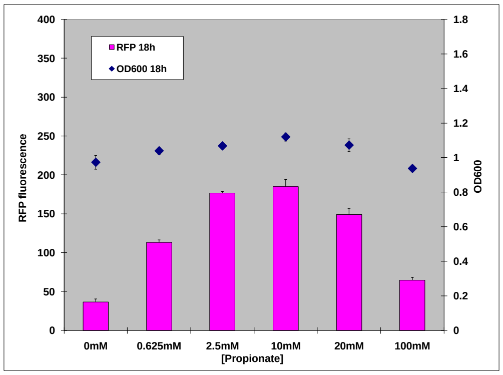

BLR(DE3) in LB, 30°C

STRAIN and MEDIA DEPENDENCE

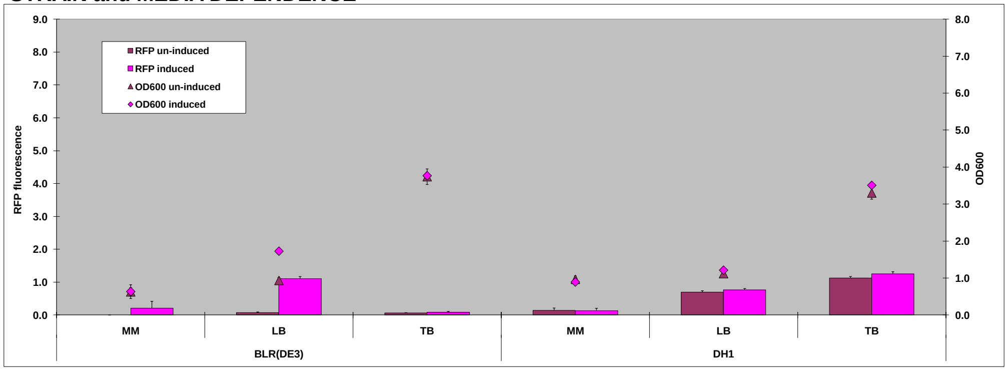

3ml cultures grown in test tubes, induced with 20mM propionate, grown at 30°C post-induction, measurements taken in Tecan 18h post-induction
MM media is supplemented with 0.5% glucose, TB media is supplemented with 2% glycerol
RFP and OD normalized to pBbE5a-RFP in BLR(DE3) in LB induced (100uM IPTG)

CATABOLITE REPRESSION

RFP/OD600 in BLR(DE3) as a percentage of induced without glucose, 18h post-induction

	LB	LB*+1%glucose	MM	MM+1%glucose	TB	TB*+1%glucose
pBbA3a induced	100.0% (+/-17.9)	11.5% (+/-0.8)	100.0% (+/-85.2)	68.5% (75.7)	N/A**	N/A**
pBbA3a un-induced	16.1% (+/-4.0)	0.0% (+/-0.0)	0.0% (+/-0.0)	0.0% (+/-0.0)	N/A**	N/A**

*100mM potassium phosphate buffered, pH 7.5
**no RFP expression detected

CROSSTALK

RFP/OD600 in BLR(DE3) in LB, 18h post-induction, pBbE3a construct

	Propionate(20mM)	Propionate(20mM) +IPTG(100uM)	Propionate(20mM) +aTc(400nM)	Propionate(20mM) +Arabinose(20mM)	Un-induced
pProS	100.0% (+/-3.9)	100.9% (+/-5.1)	126.7% (+/-0.5)	33.8% (+/-3.1)	2.2% (+/-1.9)

pBbB3

Propionate inducible promoter system

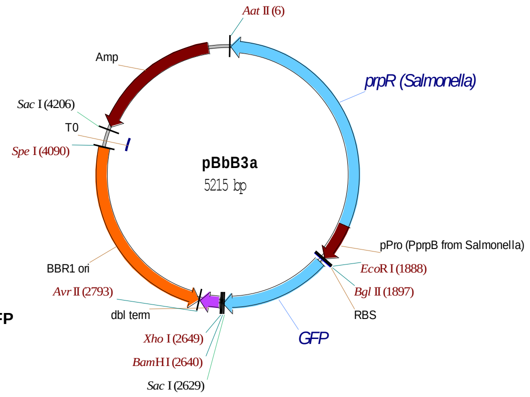

Constructs available	Freezer location (-80)
pBbB3a-GFP	2631
pBbB3k-GFP	2639
pBbB3c-GFP	2647

B = BBR1 ori (17-20 copies per cell) 3 = pProS
experiments represented on this datasheet were performed using pBbB3a-GFP
pBbE5a-GFP in BLR(DE3) in LB induced (100mM IPTG) was used as control

INDUCER DOSE RESPONSE

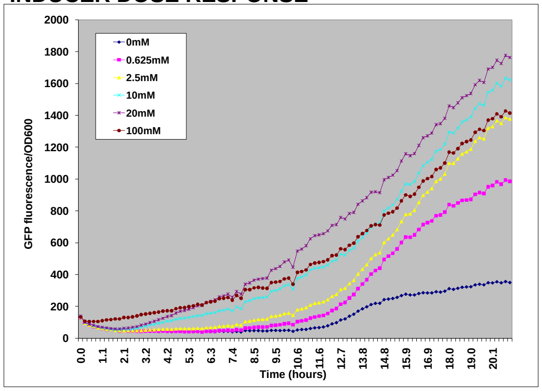

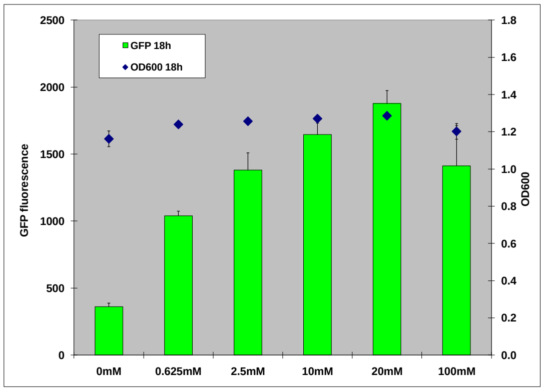

BLR(DE3) in LB, 30°C

STRAIN and MEDIA DEPENDENCE

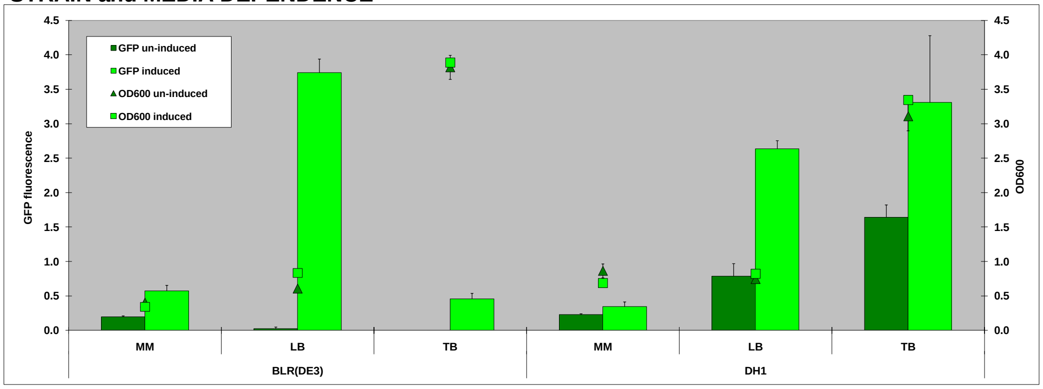

3ml cultures grown in test tubes, induced with 20mM propionate, grown at 30°C post-induction, measurements taken in Tecan 18h post-induction
MM media is supplemented with 0.5% glucose, TB media is supplemented with 2% glycerol
GFP and OD normalized to pBbE5a-GFP in BLR(DE3) in LB induced (100uM IPTG)

CATABOLITE REPRESSION

RFP/OD600 in BLR(DE3) as a percentage of induced without glucose, 18h post-induction

	LB	LB*+1%glucose	MM	MM+1%glucose	TB	TB*+1%glucose
pBbB3a induced	100.0% (+/-3.8)	5.8% (+/-2.6)	100.0% (+/-20.3)	74.6% (+/-18.8)	100.0% (+/-16.7)	18.0% (+/-13.3)
pBbB3a un-induced	0.8% (+/- 0.8)	2.4% (+/-0.5)	28.7% (+/- 3.3)	21.6% (+/-0.8)	0.0% (+/- 0.0)	12.5% (+/-10.8)

*100mM potassium phosphate buffered, pH 7.5

CROSSTALK

RFP/OD600 in BLR(DE3) in LB, 18h post-induction, pBbE3a construct

	Propionate(20mM)	Propionate(20mM) +IPTG(100uM)	Propionate(20mM) +aTc(400nM)	Propionate(20mM) +Arabinose(20mM)	Un-induced
pProS	100.0% (+/-3.9)	100.9% (+/-5.1)	126.7% (+/-0.5)	33.8% (+/-3.1)	2.2% (+/-1.9)

pBbE3

Propionate inducible promoter system

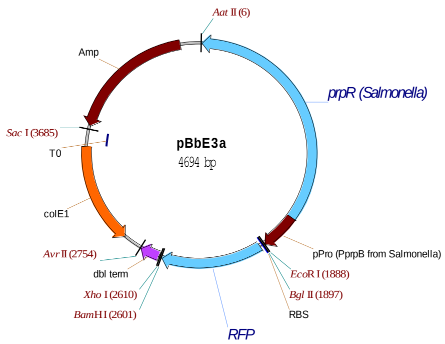

Constructs available	Freezer location (-80)
pBbE3a-RFP	2473
pBbE3k-RFP	2511
pBbE3c-RFP	2512

E = colE1 ori (20-30 copies per cell) 3 = pProS
experiments represented on this datasheet were performed using pBbE3a-RFP
pBbE5a-RFP in BLR(DE3) in LB induced (100mM IPTG) was used as control

INDUCER DOSE RESPONSE

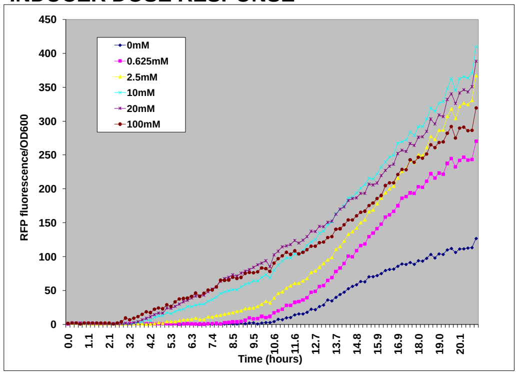

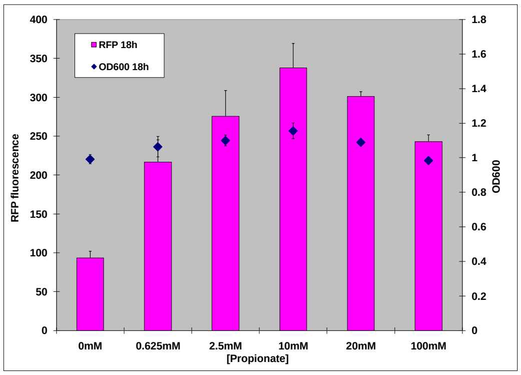

BLR(DE3) in LB, 30°C

STRAIN and MEDIA DEPENDENCE

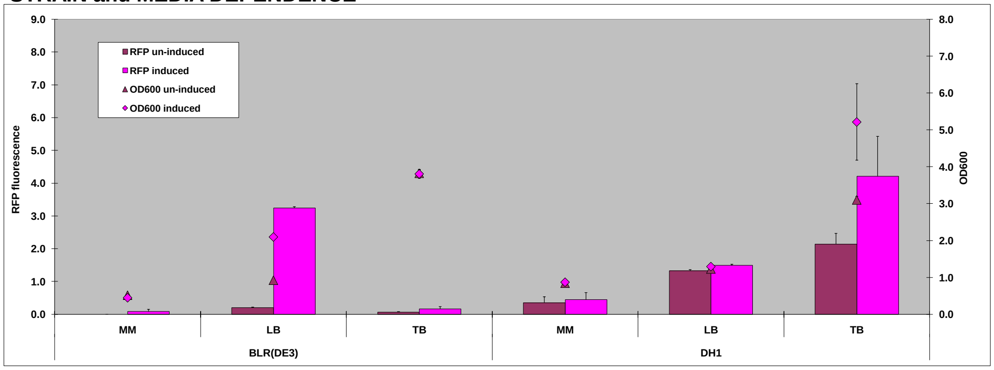

3ml cultures grown in test tubes, induced with 20mM propionate, grown at 30°C post-induction, measurements taken in Tecan 18h post-induction
MM media is supplemented with 0.5% glucose, TB media is supplemented with 2% glycerol
RFP and OD normalized to pBbE5a-RFP in BLR(DE3) in LB induced (100uM IPTG)

CATABOLITE REPRESSION

RFP/OD600 in BLR(DE3) as a percentage of induced without glucose, 18h post-induction

	LB	LB*+1%glucose	MM	MM+1%glucose	TB	TB*+1%glucose
pBbE3a induced	100.0% (5.4)	21.0% (+/-2.2)	100.0% (+/-68.3)	42.5% (54.9)	N/A**	N/A**
pBbE3a un-induced	16.3% (+/-0.4)	0.4% (+/-0.6)	0.0% (+/-0.0)	3.4% (+/-5.9)	N/A**	N/A**

*100mM potassium phosphate buffered, pH 7.5
**no RFP expression detected

CROSSTALK

RFP/OD600 in BLR(DE3) in LB, 18h post-induction, pBbE3a construct

	Propionate(20mM)	Propionate(20mM) +IPTG(100uM)	Propionate(20mM) +aTc(400nM)	Propionate(20mM) +Arabinose(20mM)	Un-induced
pProS	100.0% (+/-3.9)	100.9% (+/-5.1)	126.7% (+/-0.5)	33.8% (+/-3.1)	2.2% (+/-1.9)

pBbS3

Propionate inducible promoter system

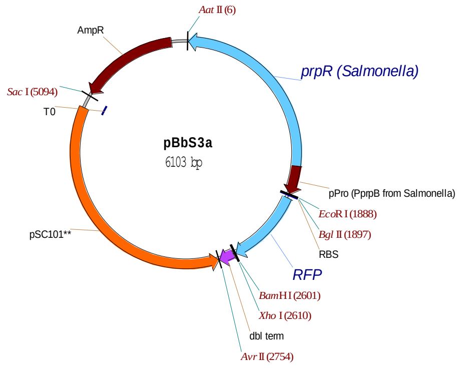

Constructs available	Freezer location (-80)
pBbS3a-RFP	2552
pBbS3k-RFP	2560
pBbS3c-RFP	2568

S = SC101 ori (4-6 copies per cell) 3 = pProS
experiments represented on this datasheet were performed using pBbS3a-RFP
pBbE5a-RFP in BLR(DE3) in LB induced (100mM IPTG) was used as control

INDUCER DOSE RESPONSE

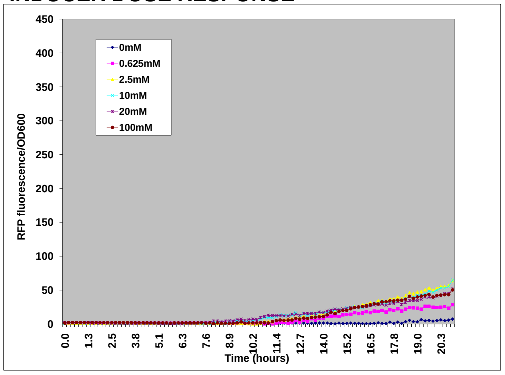

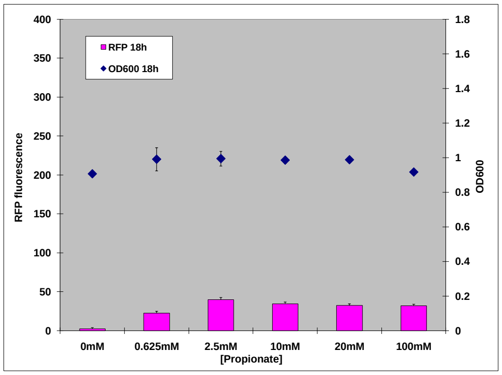

BLR(DE3) in LB, 30°C

STRAIN and MEDIA DEPENDENCE

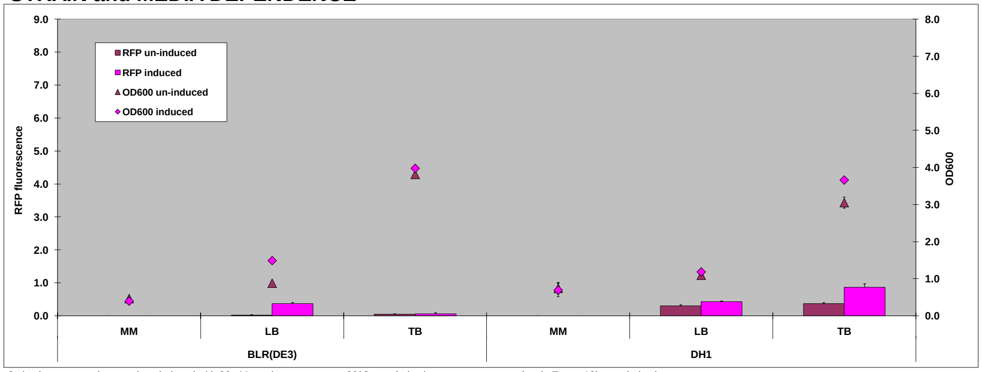

3ml cultures grown in test tubes, induced with 20mM propionate, grown at 30°C post-induction, measurements taken in Tecan 18h post-induction
MM media is supplemented with 0.5% glucose, TB media is supplemented with 2% glycerol
RFP and OD normalized to pBbE5a-RFP in BLR(DE3) in LB induced (100uM IPTG)

CATABOLITE REPRESSION

RFP/OD600 in BLR(DE3) as a percentage of induced without glucose, 18h post-induction

	LB	LB*+1%glucose	MM	MM+1%glucose	TB	TB*+1%glucose
pBbS3a induced	100.0% (+/-4.3)	0.0% (+/-0.0)	N/A**	N/A**	N/A**	N/A**
pBbE3a un-induced	2.2% (+/-3.7)	1.4% (+/-2.4)	N/A**	N/A**	N/A**	N/A**

*100mM potassium phosphate buffered, pH 7.5
**no RFP expression detected

CROSSTALK

RFP/OD600 in BLR(DE3) in LB, 18h post-induction, pBbE3a construct

	Propionate(20mM)	Propionate(20mM) +IPTG(100uM)	Propionate(20mM) +aTc(400nM)	Propionate(20mM) +Arabinose(20mM)	Un-induced
pProS	100.0% (+/-3.9)	100.9% (+/-5.1)	126.7% (+/-0.5)	33.8% (+/-3.1)	2.2% (+/-1.9)

pBbA4

Propionate inducible promoter system

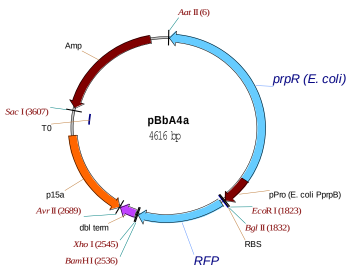

Constructs available	Freezer location (-80)
pBbA4a-RFP	2503
pBbA4k-RFP	2504
pBbA4c-RFP	2505

A = p15A ori (8-10 copies per cell) 4 = pProE
experiments represented on this datasheet were performed using pBbA4a-RFP
pBbE5a-RFP in BLR(DE3) in LB induced (100mM IPTG) was used as control

INDUCER DOSE RESPONSE

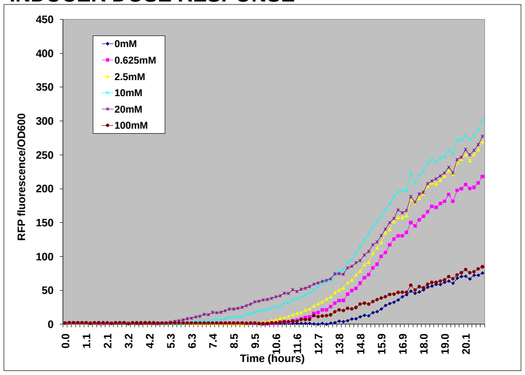

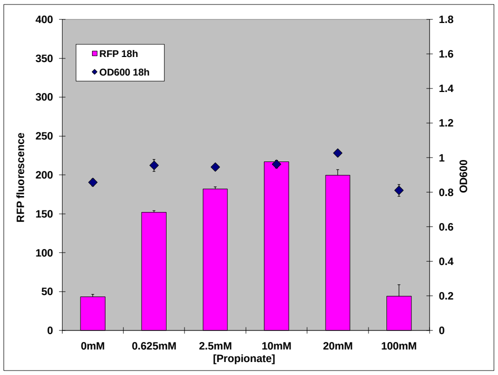

BLR(DE3) in LB, 30°C

STRAIN and MEDIA DEPENDENCE

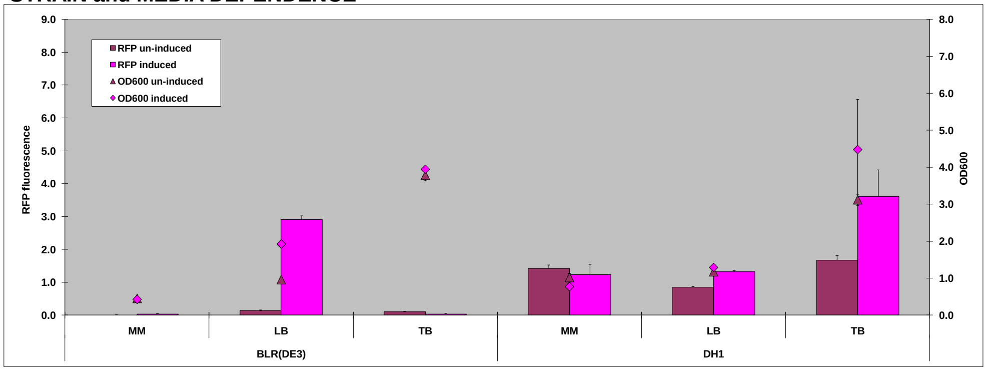

3ml cultures grown in test tubes, induced with 20mM propionate, grown at 30°C post-induction, measurements taken in Tecan 18h post-induction
MM media is supplemented with 0.5% glucose, TB media is supplemented with 2% glycerol
RFP and OD normalized to pBbE5a-RFP in BLR(DE3) in LB induced (100uM IPTG)

CATABOLITE REPRESSION

RFP/OD600 in BLR(DE3) as a percentage of induced without glucose, 18h post-induction

	LB	LB*+1%glucose	MM	MM+1%glucose	TB	TB*+1%glucose
pBbA4a induced	100.0% (+/-10.2)	0.0% (+/-0.0)	100.0% (+/-71.1)	72.9% (+/-67.1)	N/A**	N/A**
pBbA4a un-induced	23.9% (+/-0.9)	0.0% (+/-0.0)	21.0% (+/-18.2)	20.0% (+/-17.4)	N/A**	N/A**

*100mM potassium phosphate buffered, pH 7.5
**no RFP expression detected

CROSSTALK

RFP/OD600 in BLR(DE3) in LB, 18h post-induction, pBbE4a construct

	Propionate(20mM)	Propionate(20mM) +IPTG(100uM)	Propionate(20mM) +aTc(400nM)	Propionate(20mM) +Arabinose(20mM)	Un-induced
pProE	100.0% (+/-1.2)	98.8% (+/-5.1)	139.4% (+/-3.7)	20.9% (+/-0.7)	7.9% (+/-1.4)

pBbB4

Propionate inducible promoter system

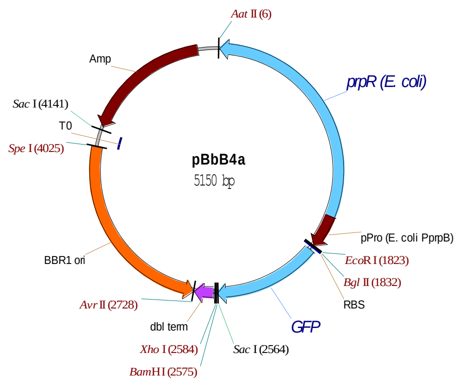

Constructs available	Freezer location (-80)
pBbB4a-GFP	2632
pBbB4k-GFP	2640
pBbB4c-GFP	2648

B = BBR1 ori (17-20 copies per cell) 4 = pProE
experiments represented on this datasheet were performed using pBbB4a-GFP
pBbE5a-GFP in BLR(DE3) in LB induced (100mM IPTG) was used as control

INDUCER DOSE RESPONSE

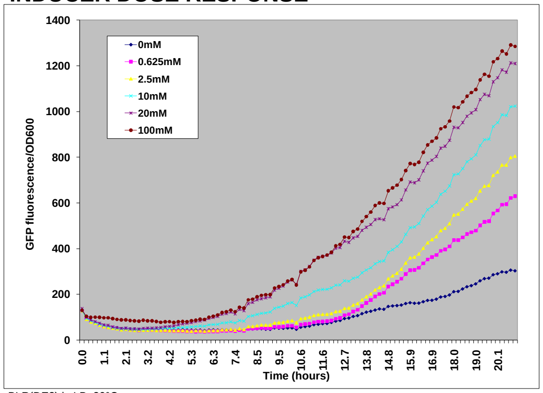

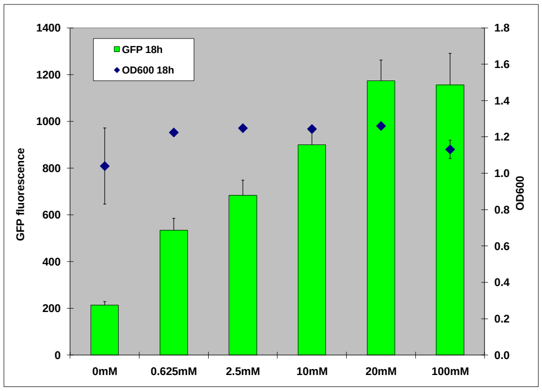

BLR(DE3) in LB, 30°C

STRAIN and MEDIA DEPENDENCE

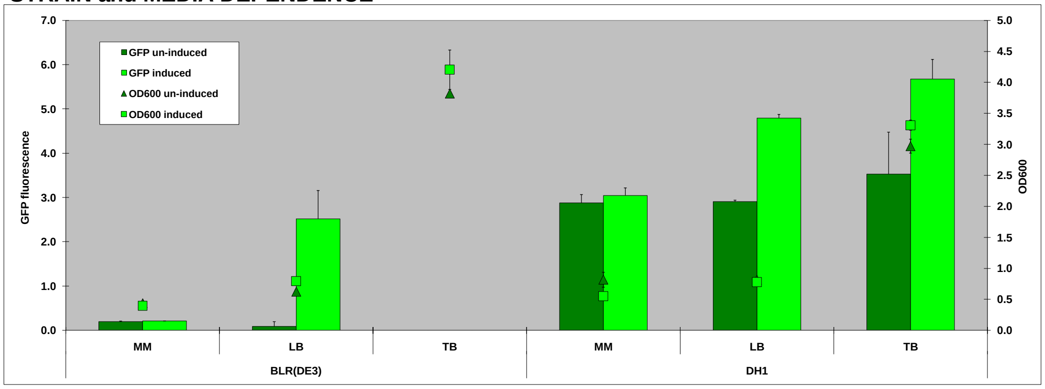

3ml cultures grown in test tubes, induced with 20mM propionate, grown at 30°C post-induction, measurements taken in Tecan 18h post-induction
MM media is supplemented with 0.5% glucose, TB media is supplemented with 2% glycerol
GFP and OD normalized to pBbE5a-GFP in BLR(DE3) in LB induced (100uM IPTG)

CATABOLITE REPRESSION

RFP/OD600 in BLR(DE3) as a percentage of induced without glucose, 18h post-induction

	LB	LB*+1%glucose	MM	MM+1%glucose	TB	TB*+1%glucose
pBbB4a induced	100.0% (+/-26.2)	2.9% (+/-1.1)	100.0% (+/-17.9)	91.3% (+/-5.9)	N/A**	N/A**
pBbB4a un-induced	4.2% (+/-5.2)	3.0% (+/-0.9)	83.4% (+/-7.2)	75.0% (+/-6.7)	N/A**	N/A**

*100mM potassium phosphate buffered, pH 7.5
**no GFP expression detected

CROSSTALK

RFP/OD600 in BLR(DE3) in LB, 18h post-induction, pBbE4a construct

	Propionate(20mM)	Propionate(20mM) +IPTG(100uM)	Propionate(20mM) +aTc(400nM)	Propionate(20mM) +Arabinose(20mM)	Un-induced
pProE	100.0% (+/-1.2)	98.8% (+/-5.1)	139.4% (+/-3.7)	20.9% (+/-0.7)	7.9% (+/-1.4)

pBbE4

Propionate inducible promoter system

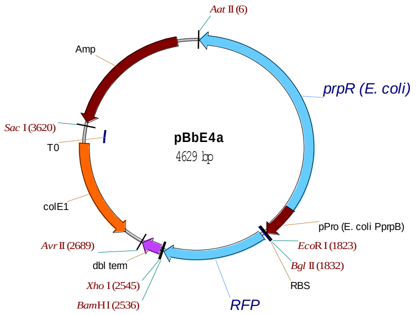

Constructs available	Freezer location (-80)
pBbE4a-RFP	2472
pBbE4k-RFP	2506
pBbE4c-RFP	2507

E = colE1 ori (20-30 copies per cell) 4 = pProE
experiments represented on this datasheet were performed using pBbE4a-RFP
pBbE5a-RFP in BLR(DE3) in LB induced (100mM IPTG) was used as control

INDUCER DOSE RESPONSE

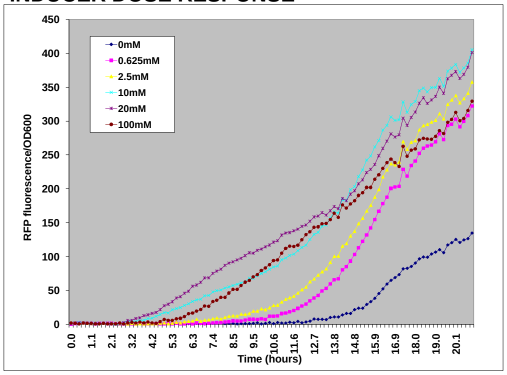

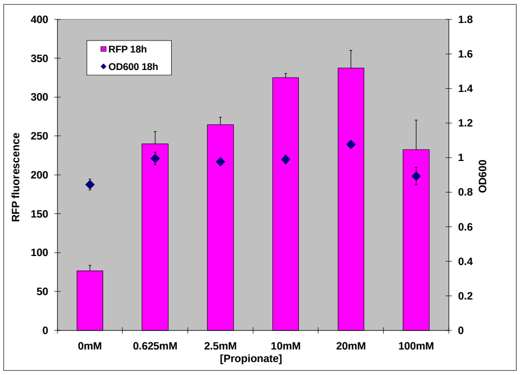

BLR(DE3) in LB, 30°C

STRAIN and MEDIA DEPENDENCE

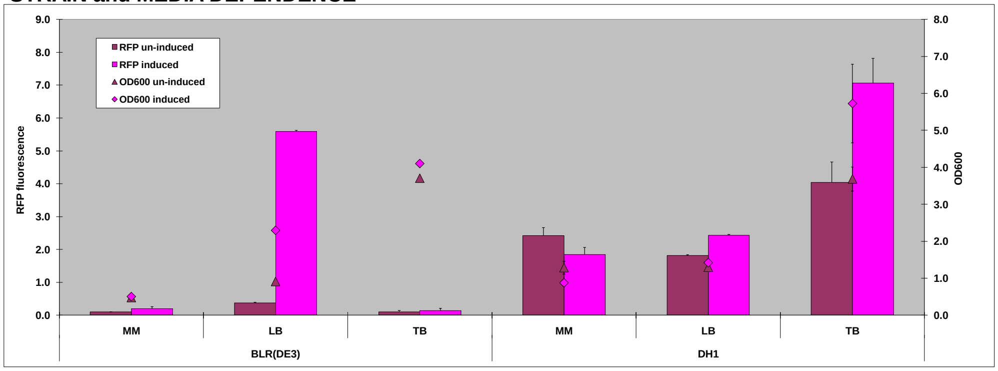

3ml cultures grown in test tubes, induced with 20mM propionate, grown at 30°C post-induction, measurements taken in Tecan 18h post-induction
MM media is supplemented with 0.5% glucose, TB media is supplemented with 2% glycerol
RFP and OD normalized to pBbE5a-RFP in BLR(DE3) in LB induced (100uM IPTG)

CATABOLITE REPRESSION

RFP/OD600 in BLR(DE3) as a percentage of induced without glucose, 18h post-induction

	LB	LB*+1%glucose	MM	MM+1%glucose	TB	TB*+1%glucose
pBbE4a induced	100.0% (+/-11.0)	2.2% (+/-0.4)	100.0% (+/- 32.4)	83.0% (+/-19.5)	N/A**	N/A**
pBbE4a un-induced	25.8% (+/-1.3)	0.0% (+/-0.0)	51.2% (+/- 7.8)	46.6% (+/-7.8)	N/A**	N/A**

*100mM potassium phosphate buffered, pH 7.5
**no RFP expression detected

CROSSTALK

RFP/OD600 in BLR(DE3) in LB, 18h post-induction, pBbE4a construct

	Propionate(20mM)	Propionate(20mM) +IPTG(100uM)	Propionate(20mM) +aTc(400nM)	Propionate(20mM) +Arabinose(20mM)	Un-induced
pProE	100.0% (+/-1.2)	98.8% (+/-5.1)	139.4% (+/-3.7)	20.9% (+/-0.7)	7.9% (+/-1.4)

pBbS4

Propionate inducible promoter system

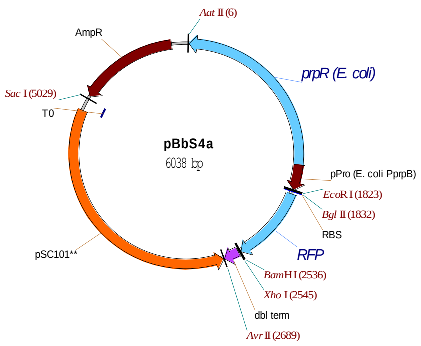

Constructs available	Freezer location (-80)
pBbS4a-RFP	2551
pBbS4k-RFP	2559
pBbS4c-RFP	2567

S = SC101 ori (4-6 copies per cell) 4 = pProE
experiments represented on this datasheet were performed using pBbS4a-RFP
pBbE5a-RFP in BLR(DE3) in LB induced (100mM IPTG) was used as control

INDUCER DOSE RESPONSE

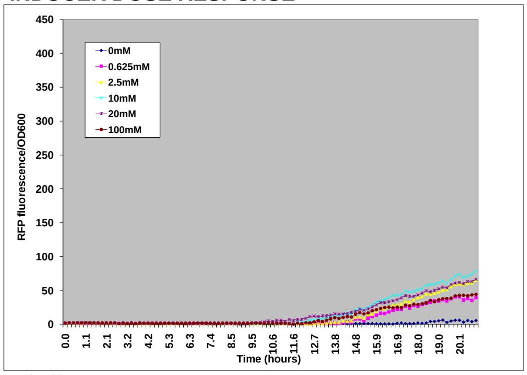

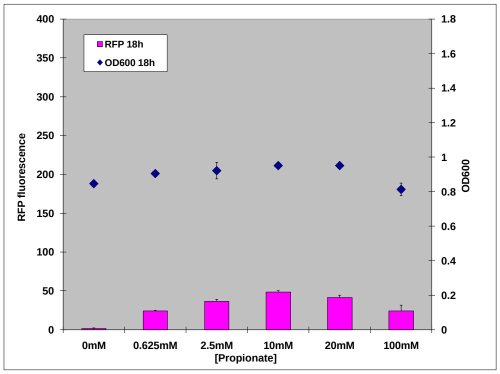

BLR(DE3) in LB, 30°C

STRAIN and MEDIA DEPENDENCE

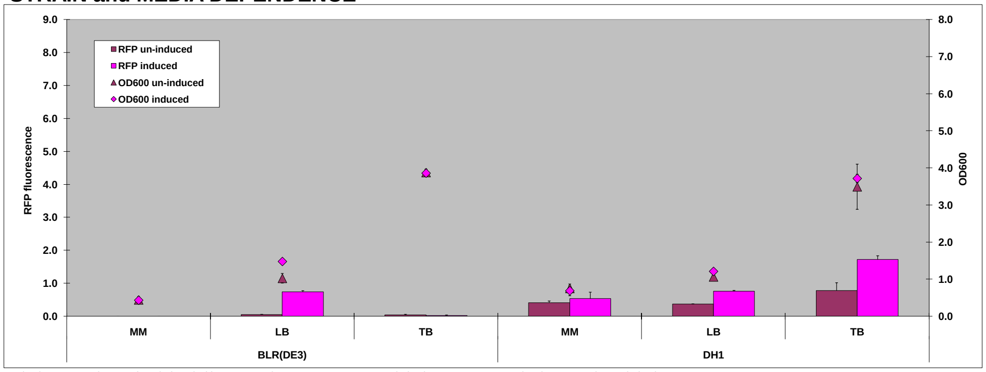

3ml cultures grown in test tubes, induced with 20mM propionate, grown at 30°C post-induction, measurements taken in Tecan 18h post-induction
MM media is supplemented with 0.5% glucose, TB media is supplemented with 2% glycerol
RFP and OD normalized to pBbE5a-RFP in BLR(DE3) in LB induced (100uM IPTG)

CATABOLITE REPRESSION

RFP/OD600 in BLR(DE3) as a percentage of induced without glucose, 18h post-induction

	LB	LB*+1%glucose	MM	MM+1%glucose	TB	TB*+1%glucose
pBbS4a induced	100.0% (+/-9.0)	0.0% (+/-0.0)	N/A**	N/A**	N/A**	N/A**
pBbS4a un-induced	7.0% (6.1)	0.0% (+/-0.0)	N/A**	N/A**	N/A**	N/A**

*100mM potassium phosphate buffered, pH 7.5
**no RFP expression detected

CROSSTALK

RFP/OD600 in BLR(DE3) in LB, 18h post-induction, pBbE4a construct

	Propionate(20mM)	Propionate(20mM) +IPTG(100uM)	Propionate(20mM) +aTc(400nM)	Propionate(20mM) +Arabinose(20mM)	Un-induced
pProE	100.0% (+/-1.2)	98.8% (+/-5.1)	139.4% (+/-3.7)	20.9% (+/-0.7)	7.9% (+/-1.4)

pBbA5

IPTG inducible promoter system

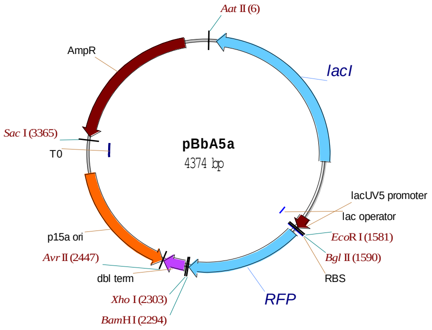

Constructs available	Freezer location (-80)
pBbA5a-RFP	2475
pBbA5k-RFP	2481
pBbA5c-RFP	2488

A = p15A ori (8-10 copies per cell) 5 = placUV5
experiments represented on this datasheet were performed using pBbA5a-RFP
pBbE5a-RFP in BLR(DE3) in LB induced (100mM IPTG) was used as control

INDUCER DOSE RESPONSE

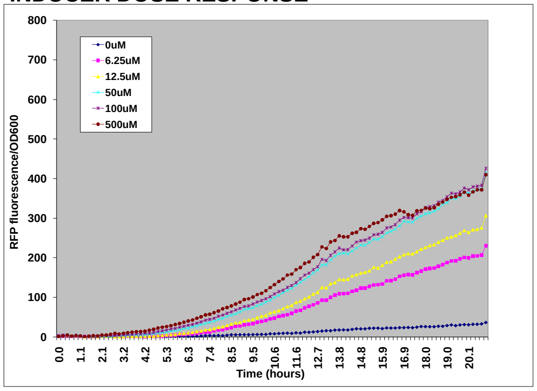

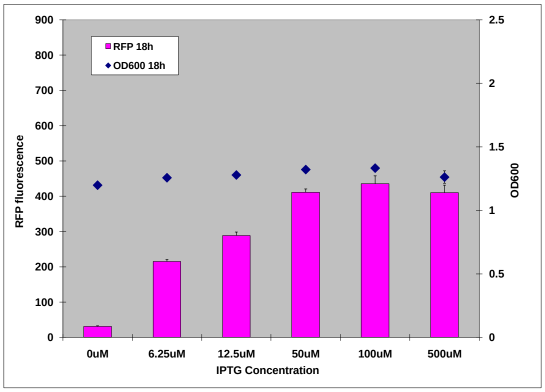

BLR(DE3) in LB, 30°C

STRAIN and MEDIA DEPENDENCE

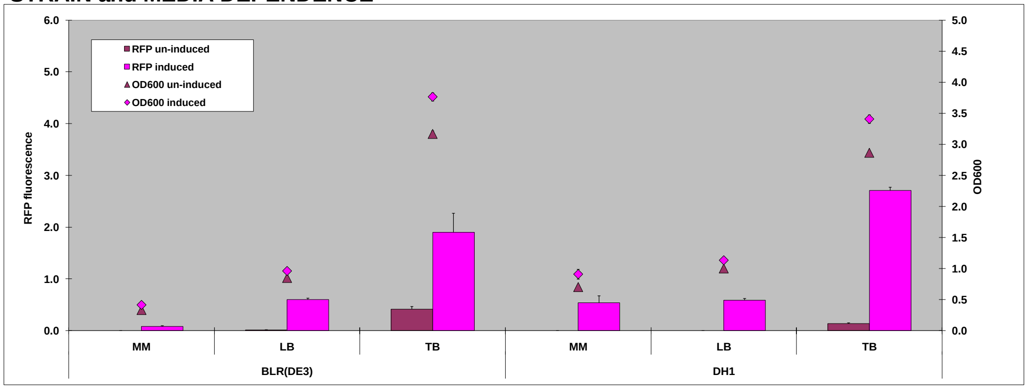

3ml cultures grown in test tubes, induced with 100uM IPTG, grown at 30°C post-induction, measurements taken in Tecan 18h post-induction
MM media is supplemented with 0.5% glucose, TB media is supplemented with 2% glycerol
RFP and OD normalized to pBbE5a-RFP in BLR(DE3) in LB induced (100uM IPTG)

CATABOLITE REPRESSION

RFP/OD600 in BLR(DE3) as a percentage of induced without glucose, 18h post-induction

	LB	LB*+1%glucose	MM	MM+1%glucose	TB	TB*+1%glucose
pBbA5a induced	100.0% (+/-2.6)	37.1% (+/-3.6)	100.0% (+/-0.0)	43.6% (+/-0.0)	100.0% (+/-12.6)	56.3% (+/-6.0)
pBbA5a un-induced	2.4% (+/-1.1)	1.1% (+/-0.0)	0.0% (+/-0.0)	0.0% (+/-0.0)	30.5% (+/-10.8)	2.8% (+/-0.0)

*100mM potassium phosphate buffered, pH 7.5

CROSSTALK

RFP/OD600 in BLR(DE3) in LB, 18h post-induction, pBbE5a construct

	IPTG(100uM)	IPTG(100uM) +aTc(400nM)	IPTG(100uM) +Arabinose(20mM)	IPTG(100uM) +Propionate(20mM)	Un-induced
placUV5	100.0% (+/-6.7)	141.3% (+/-6.7)	97.8% (+/-2.8)	128.0% (+/-9.7)	0.0% (+/-0.0)

pBbB5

IPTG inducible promoter system

Constructs available	Freezer location (-80)
pBbB5a-GFP	2633
pBbB5k-GFP	2641
pBbB5c-GFP	2649

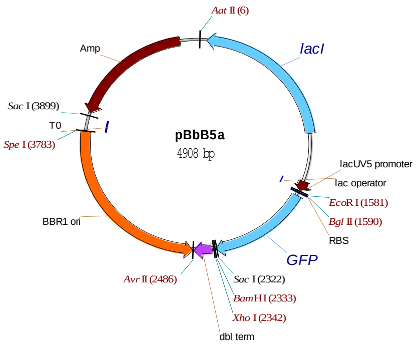

B = BBR1 ori (17-20 copies per cell) 5 = placUV5
experiments represented on this datasheet were performed using pBbB5a-GFP
pBbE5a-GFP in BLR(DE3) in LB induced (100mM IPTG) was used as control

INDUCER DOSE RESPONSE

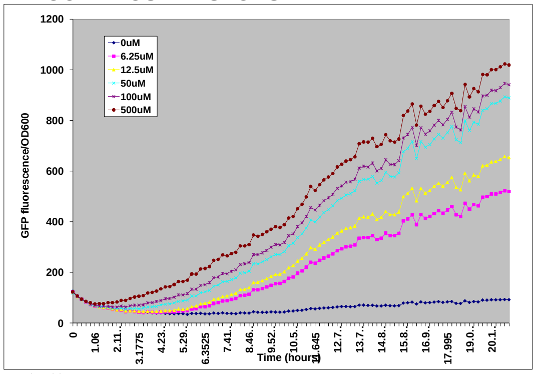

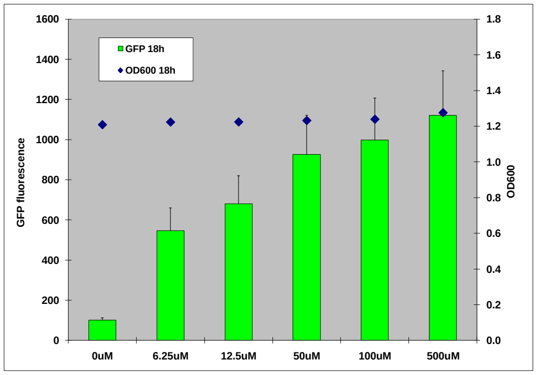

BLR(DE3) in LB, 30°C

STRAIN and MEDIA DEPENDENCE

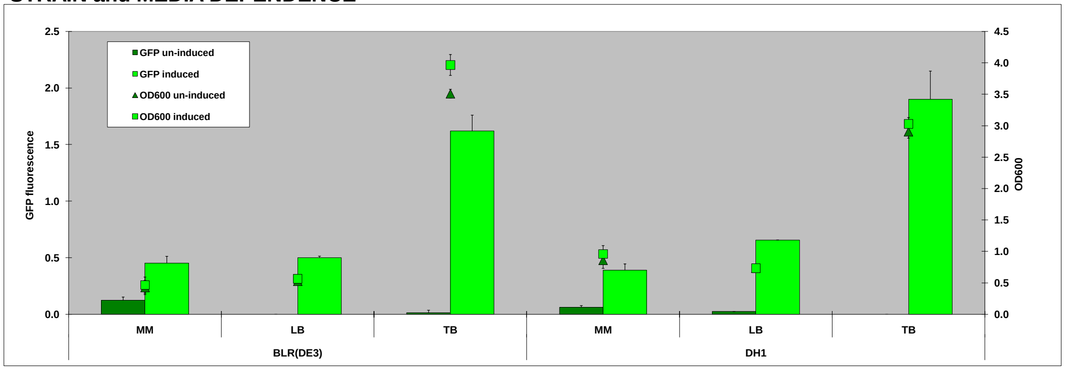

3ml cultures grown in test tubes, induced with 100uM IPTG, grown at 30°C post-induction, measurements taken in Tecan 18h post-induction
MM media is supplemented with 0.5% glucose, TB media is supplemented with 2% glycerol
GFP and OD normalized to pBbE5a-GFP in BLR(DE3) in LB induced (100uM IPTG)

CATABOLITE REPRESSION

RFP/OD600 in BLR(DE3) as a percentage of induced without glucose, 18h post-induction

	LB	LB*+1%glucose	MM	MM+1%glucose	TB	TB*+1%glucose
pBbB5a induced	100.0% (+/- 1.8)	89.5% (+/-10.3)	100.0% (+/- 44.7)	99.5% (+/-47.2)	100.0% (+/- 63.8)	169.4% (+/-5.4)
pBbB5a un-induced	0.0% (+/- 0.0)	8.7% (+/-1.5)	30.0% (+/- 13.6)	23.2% (+/-4.7)	0.4% (+/- 0.6)	0.0% (+/-0.0)

*100mM potassium phosphate buffered, pH 7.5

CROSSTALK

RFP/OD600 in BLR(DE3) in LB, 18h post-induction, pBbE5a construct

	IPTG(100uM)	IPTG(100uM) +aTc(400nM)	IPTG(100uM) +Arabinose(20mM)	IPTG(100uM) +Propionate(20mM)	Un-induced
placUV5	100.0% (+/-6.7)	141.3% (+/-6.7)	97.8% (+/-2.8)	128.0% (+/-9.7)	0.0% (+/-0.0)

pBbE5

IPTG inducible promoter system

Constructs available	Freezer location (-80)
pBbE5a-RFP	2467
pBbE5k-RFP	2494
pBbE5c-RFP	2466

E = ColE1 ori (20-30 copies per cell) 5 = placUV5
experiments represented on this datasheet were performed using pBbE5a-RFP
pBbE5a-RFP in BLR(DE3) in LB induced (100mM IPTG) was used as control

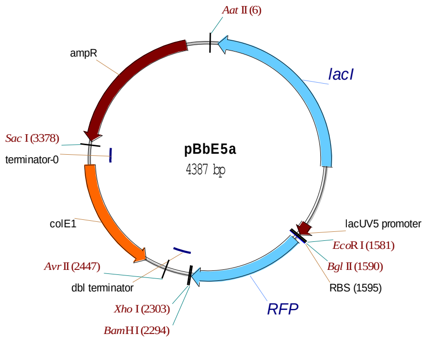

INDUCER DOSE RESPONSE

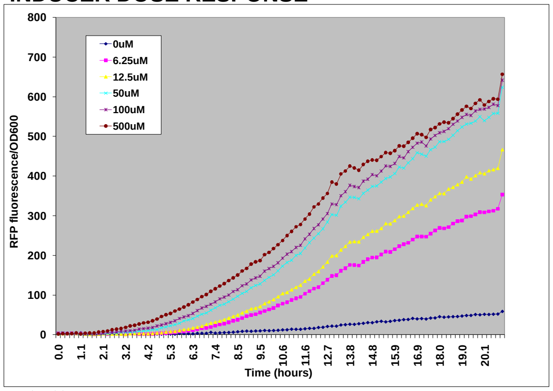

BLR(DE3) in LB, 30°C

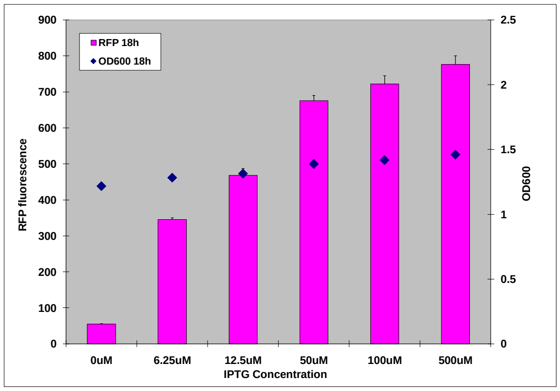

STRAIN and MEDIA DEPENDENCE

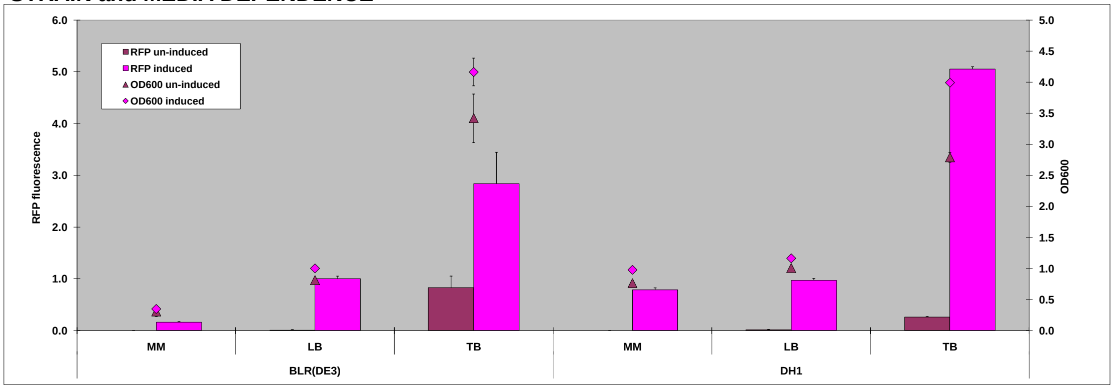

3ml cultures grown in test tubes, induced with 100uM IPTG, grown at 30°C post-induction, measurements taken in Tecan 18h post-induction
MM media is supplemented with 0.5% glucose, TB media is supplemented with 2% glycerol
RFP and OD normalized to pBbE5a-RFP in BLR(DE3) in LB induced (100uM IPTG)

CATABOLITE REPRESSION

RFP/OD600 in BLR(DE3) as a percentage of induced without glucose, 18h post-induction

	LB	LB*+1%glucose	MM	MM+1%glucose	TB	TB*+1%glucose
pBbE5a induced	100.0% (+/- 6.0)	48.7% (+/-3.5)	100.0% (+/- 0.0)	28.4% (+/-0.0)	100.0% (+/- 4.9)	69.8% (+/-5.3)
pBbE5a un-induced	1.1% (+/- 1.2)	0.7% (+/-0.0)	0.0% (+/- 0.0)	0.0% (+/-0.0)	35.5% (+/- 8.7)	4.0% (+/-1.6)

*100mM potassium phosphate buffered, pH 7.5

CROSSTALK

RFP/OD600 in BLR(DE3) in LB, 18h post-induction, pBbE5a construct

	IPTG(100uM)	IPTG(100uM) +aTc(400nM)	IPTG(100uM) +Arabinose(20mM)	IPTG(100uM) +Propionate(20mM)	Un-induced
placUV5	100.0% (+/-6.7)	141.3% (+/-6.7)	97.8% (+/-2.8)	128.0% (+/-9.7)	0.0% (+/-0.0)

pBbS5

IPTG inducible promoter system

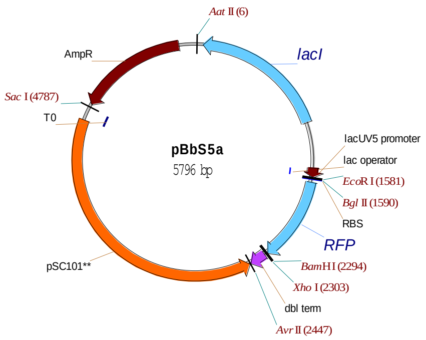

Constructs available	Freezer location (-80)
pBbS5a-RFP	2474
pBbS5k-RFP	2553
pBbS5c-RFP	2561

S = SC101 ori (4-6 copies per cell) 5 = placUV5
experiments represented on this datasheet were performed using pBbS5a-RFP
pBbE5a-RFP in BLR(DE3) in LB induced (100mM IPTG) was used as control

INDUCER DOSE RESPONSE

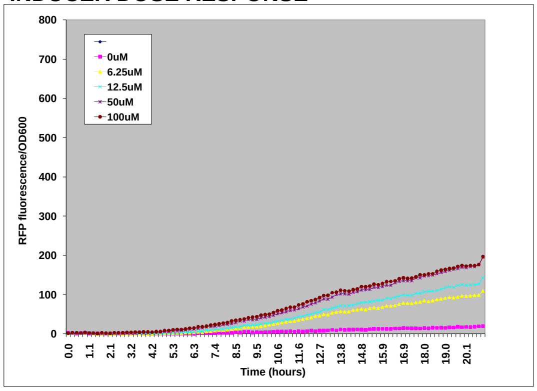

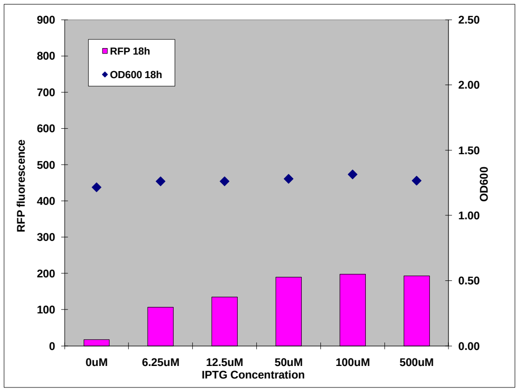

BLR(DE3) in LB, 30°C

STRAIN and MEDIA DEPENDENCE

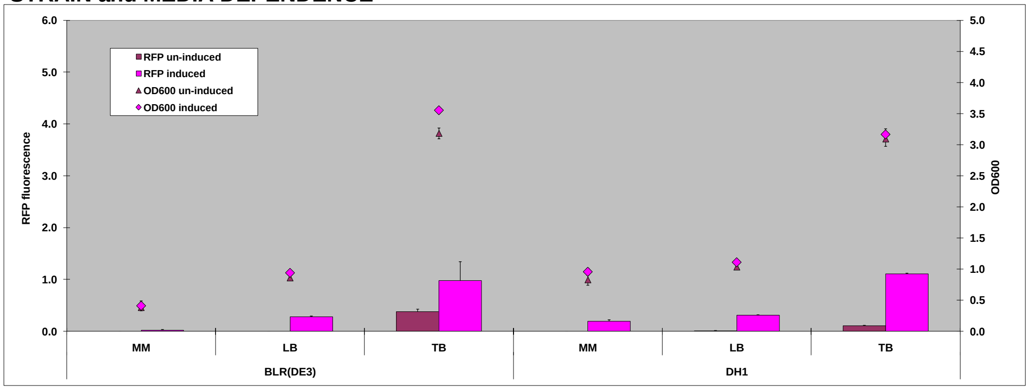

3ml cultures grown in test tubes, induced with 100uM IPTG, grown at 30°C post-induction, measurements taken in Tecan 18h post-induction
MM media is supplemented with 0.5% glucose, TB media is supplemented with 2% glycerol
RFP and OD normalized to pBbE5a-RFP in BLR(DE3) in LB induced (100uM IPTG)

CATABOLITE REPRESSION

RFP/OD600 in BLR(DE3) as a percentage of induced without glucose, 18h post-induction

	LB	LB*+1%glucose	MM	MM+1%glucose	TB	TB*+1%glucose
pBbS5a induced	100. % (+/-8.0)	31.4% (+/-2.7)	N/A**	N/A**	100.0% (+/-6.1)	70.3% (+/-12.4)
pBbS5a un-induced	2.5% (+/-2.2)	2.4% (0.0)	N/A**	N/A**	41.1% (+/-3.5)	4.2% (2.1)

*100mM potassium phosphate buffered, pH 7.5
**no RFP fluorescence detected

CROSSTALK

RFP/OD600 in BLR(DE3) in LB, 18h post-induction, pBbE5a construct

	IPTG(100uM)	IPTG(100uM) +aTc(400nM)	IPTG(100uM) +Arabinose(20mM)	IPTG(100uM) +Propionate(20mM)	Un-induced
placUV5	100.0% (+/-6.7)	141.3% (+/-6.7)	97.8% (+/-2.8)	128.0% (+/-9.7)	0.0% (+/-0.0)

pBbA6

IPTG inducible promoter system

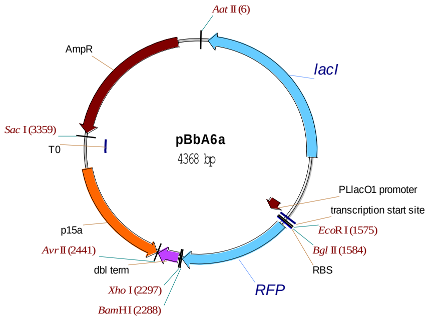

Constructs available	Freezer location (-80)
pBbA6a-RFP	2476
pBbA6k-RFP	2482
pBbA6c-RFP	2489

A = p15A ori (8-10 copies per cell) 6 = pLlacO-1
experiments represented on this datasheet were performed using pBbA6a-RFP
pBbE5a-RFP in BLR(DE3) in LB induced (100mM IPTG) was used as control

INDUCER DOSE RESPONSE

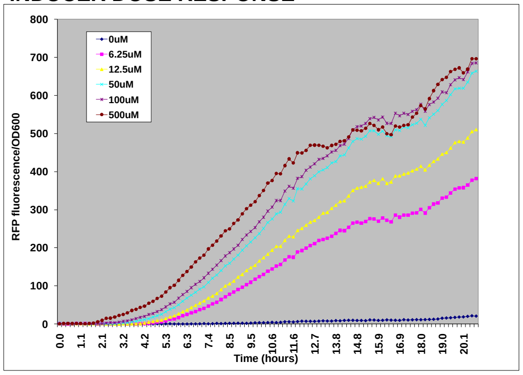

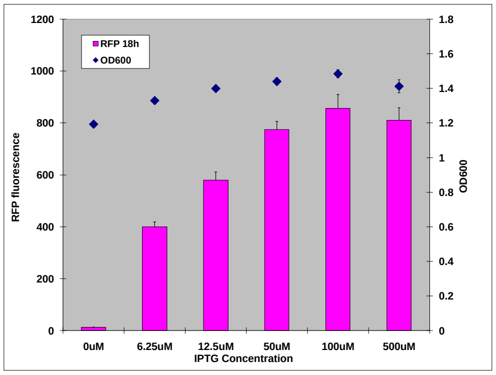

BLR(DE3) in LB, 30°C

STRAIN and MEDIA DEPENDENCE

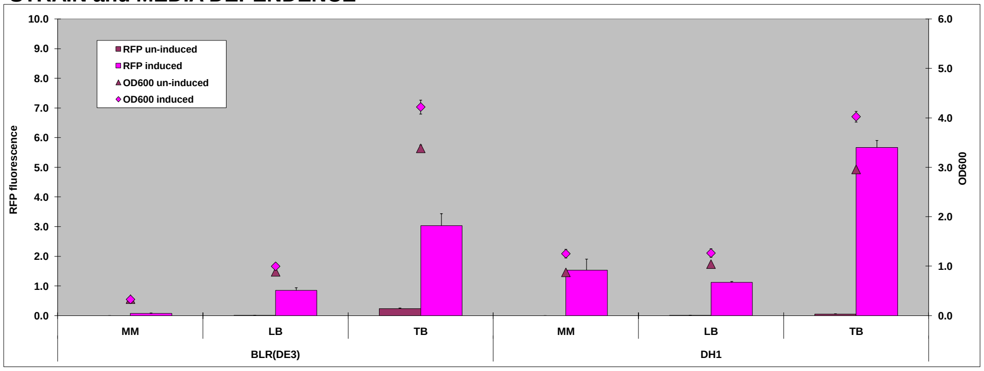

3ml cultures grown in test tubes, induced with 100uM IPTG, grown at 30°C post-induction, measurements taken in Tecan 18h post-induction
MM media is supplemented with 0.5% glucose, TB media is supplemented with 2% glycerol
RFP and OD normalized to pBbE5a-RFP in BLR(DE3) in LB induced (100uM IPTG)

CATABOLITE REPRESSION

RFP/OD600 in BLR(DE3) as a percentage of induced without glucose, 18h post-induction

	LB	LB*+1%glucose	MM	MM+1%glucose	TB	TB*+1%glucose
pBbA6a induced	100.0% (+/-0.4)	25.0% (+/-13.3)	100.0% (+/-0.0)	38.9% (+/-0.0)	100.0% (+/-28.6)	47.8% (+/-7.5)
pBbA6a un-induced	1.4% (+/-0.0)	0.8% (+/-0.0)	0.0% (+/-0.0)	0.0% (+/-0.0)	9.9% (+/-0.7)	1.4% (+/-0.7)

*100mM potassium phosphate buffered, pH 7.5

CROSSTALK

RFP/OD600 in BLR(DE3) in LB, 18h post-induction, pBbE6a construct

	IPTG(100uM)	IPTG(100uM) +aTc(400nM)	IPTG(100uM) +Arabinose(20mM)	IPTG(100uM) +Propionate(20mM)	Un-induced
pLlacO-1	100.0% (+/-3.8)	138.5% (+/-0.9)	84.1% (+/-5.4)	138.7% (+/-5.1)	0.0% (+/-0.0)

pBbB6

IPTG inducible promoter system

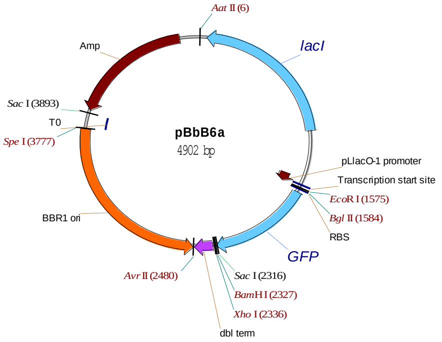

Constructs available	Freezer location (-80)
pBbB6a-GFP	2634
pBbB6k-GFP	2642
pBbB6c-GFP	2650

B = BBR1 ori (17-20 copies per cell) 6 = pLlacO-1
experiments represented on this datasheet were performed using pBbB6a-GFP
pBbE5a-GFP in BLR(DE3) in LB induced (100mM IPTG) was used as control

INDUCER DOSE RESPONSE

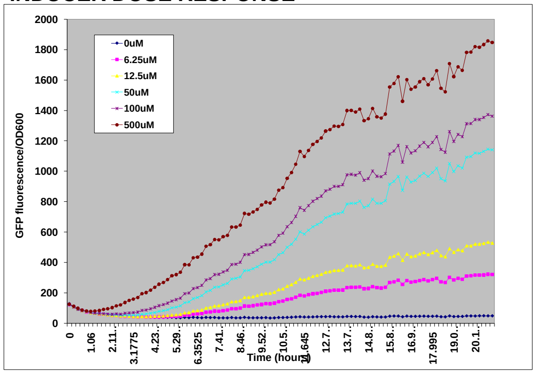

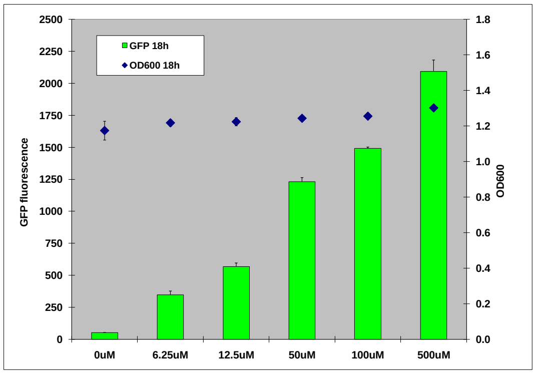

BLR(DE3) in LB, 30°C

STRAIN and MEDIA DEPENDENCE

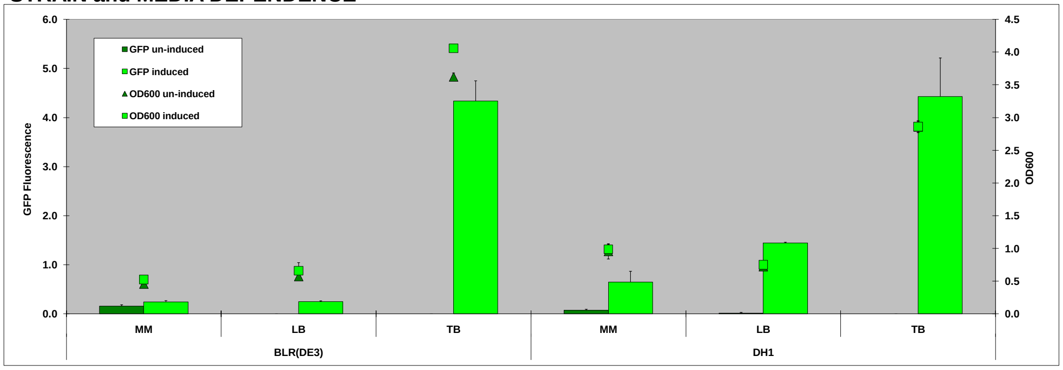

3ml cultures grown in test tubes, induced with 100uM IPTG, grown at 30°C post-induction, measurements taken in Tecan 18h post-induction
MM media is supplemented with 0.5% glucose, TB media is supplemented with 2% glycerol
GFP and OD normalized to pBbE5a-GFP in BLR(DE3) in LB induced (100uM IPTG)

CATABOLITE REPRESSION

RFP/OD600 in BLR(DE3) as a percentage of induced without glucose, 18h post-induction

	LB	LB*+1%glucose	MM	MM+1%glucose	TB	TB*+1%glucose
pBbB6a induced	100.0% (+/-16.4)	68.3% (+/-6.0)	100.0% (+/-9.3)	114.2% (+/-12.0)	100.0% (+/-9.5)	161.2% (+/-4.1)
pBbB6a un-induced	0.0% (+/-0.0)	20.5% (+/-11.3)	75.0% (+/-16.4)	67.2% (+/-15.9)	0.0% (+/-0.0)	0.7% (+/-0.6)

*100mM potassium phosphate buffered, pH 7.5

CROSSTALK

RFP/OD600 in BLR(DE3) in LB, 18h post-induction, pBbE6a construct

	IPTG(100uM)	IPTG(100uM) +aTc(400nM)	IPTG(100uM) +Arabinose(20mM)	IPTG(100uM) +Propionate(20mM)	Un-induced
pLlacO-1	100.0% (+/-3.8)	138.5% (+/-0.9)	84.1% (+/-5.4)	138.7% (+/-5.1)	0.0% (+/-0.0)

pBbE6

IPTG inducible promoter system

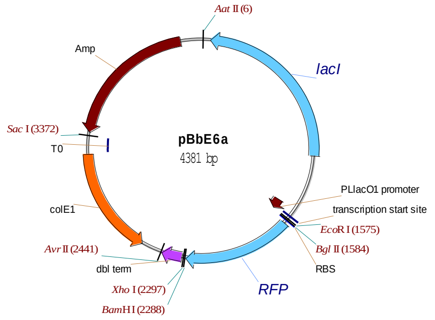

Constructs available	Freezer location (-80)
pBbE6a-RFP	2468
pBbE6k-RFP	2495
pBbE6c-RFP	2465

E = colE1 ori (20-30 copies per cell) 6 = pLlacO-1
experiments represented on this datasheet were performed using pBbE6a-RFP
pBbE5a-RFP in BLR(DE3) in LB induced (100mM IPTG) was used as control

INDUCER DOSE RESPONSE

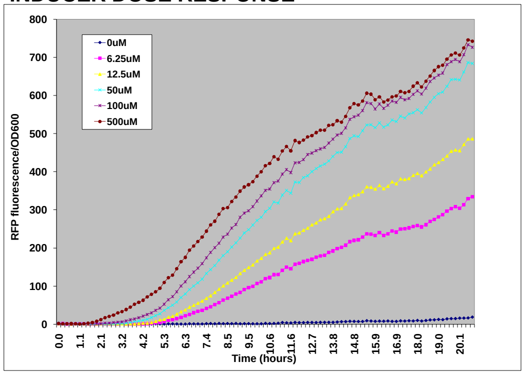

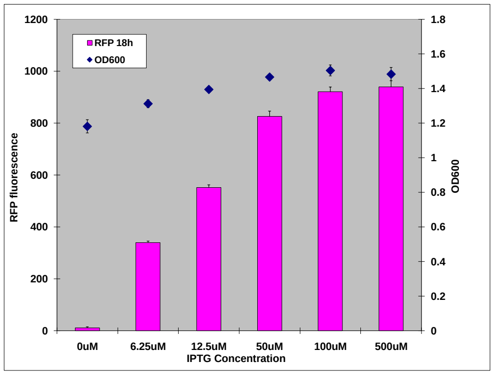

BLR(DE3) in LB, 30°C

STRAIN and MEDIA DEPENDENCE

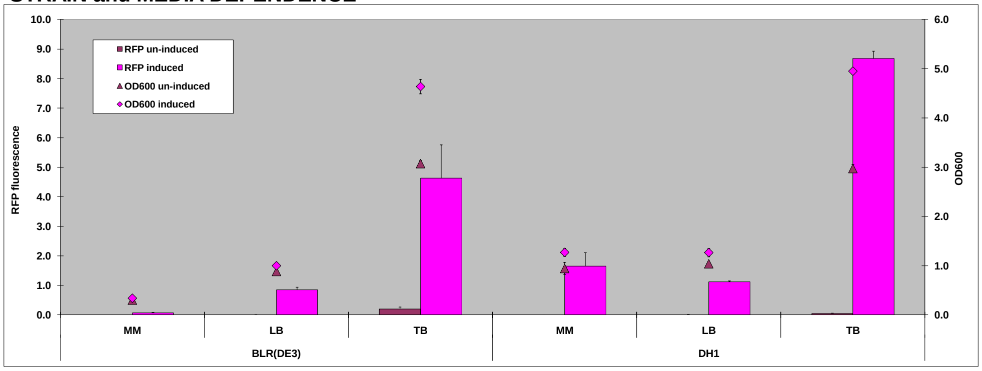

3ml cultures grown in test tubes, induced with 100uM IPTG, grown at 30°C post-induction, measurements taken in Tecan 18h post-induction
MM media is supplemented with 0.5% glucose, TB media is supplemented with 2% glycerol
RFP and OD normalized to pBbE5a-RFP in BLR(DE3) in LB induced (100uM IPTG)

CATABOLITE REPRESSION

RFP/OD600 in BLR(DE3) as a percentage of induced without glucose, 18h post-induction

	LB	LB*+1%glucose	MM	MM+1%glucose	TB	TB*+1%glucose
pBbE6a induced	100.0% (+/-9.2)	19.2% (1.7)	100.0% (+/-0.0)	33.6% (+/-0.0)	100.0% (+/-19.9)	51.7% (11.3)
pBbE6a un-induced	1.5% (+/-0.1)	1.0% (+/-0.1)	0.0% (+/-0.0)	0.0% (+/-0.0)	6.0% (+/-2.0)	0.3% (+/-0.6)

*100mM potassium phosphate buffered, pH 7.5

CROSSTALK

RFP/OD600 in BLR(DE3) in LB, 18h post-induction, pBbE6a construct

	IPTG(100uM)	IPTG(100uM) +aTc(400nM)	IPTG(100uM) +Arabinose(20mM)	IPTG(100uM) +Propionate(20mM)	Un-induced
pLlacO-1	100.0% (+/-3.8)	138.5% (+/-0.9)	84.1% (+/-5.4)	138.7% (+/-5.1)	0.0% (+/-0.0)

pBbS6

IPTG inducible promoter system

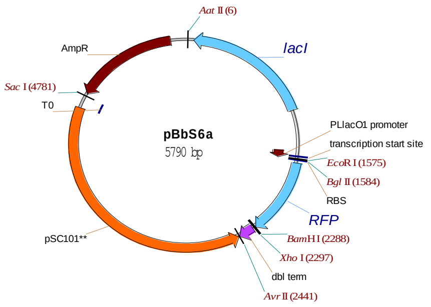

Constructs available	Freezer location (-80)
pBbS6a-RFP	2546
pBbS6k-RFP	2554
pBbS6c-RFP	2562

S = SC101 ori (4-6 copies per cell) 6 = pLlacO-1
experiments represented on this datasheet were performed using pBbS6a-RFP
pBbE5a-RFP in BLR(DE3) in LB induced (100mM IPTG) was used as control

INDUCER DOSE RESPONSE

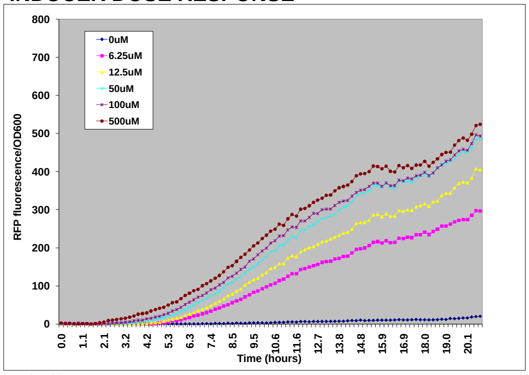

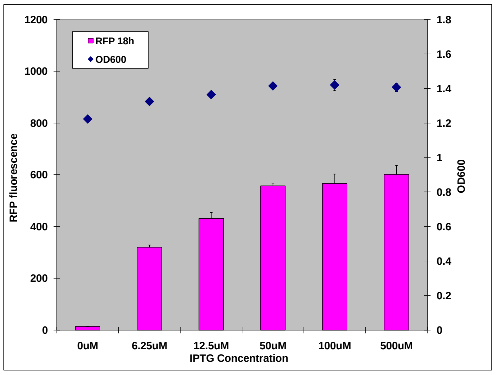

BLR(DE3) in LB, 30°C

STRAIN and MEDIA DEPENDENCE

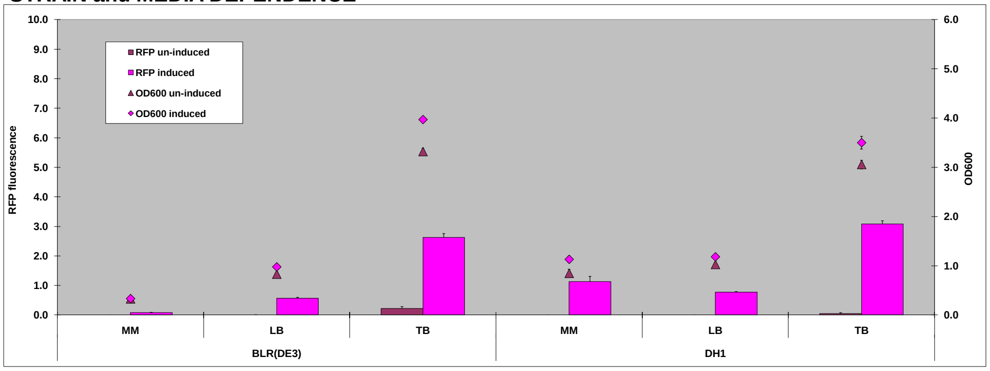

3ml cultures grown in test tubes, induced with 100uM IPTG, grown at 30°C post-induction, measurements taken in Tecan 18h post-induction
MM media is supplemented with 0.5% glucose, TB media is supplemented with 2% glycerol
RFP and OD normalized to pBbE5a-RFP in BLR(DE3) in LB induced (100uM IPTG)

CATABOLITE REPRESSION

RFP/OD600 in BLR(DE3) as a percentage of induced without glucose, 18h post-induction

	LB	LB*+1%glucose	MM	MM+1%glucose	TB	TB*+1%glucose
pBbS6a induced	100.0% (+/-37.3)	23.3% (+/-4.5)	100.0% (+/-0.0)	26.6% (+/-0.0)	100.0% (+/-18.0)	54.1% (+/-7.9)
pBbS6a un-induced	2.2% (+/-0.0)	1.4% (+/-0.0)	0.0% (+/-0.0)	0.0% (+/-0.0)	10.2% (+/-2.5)	1.6% (+/-0.4)

*100mM potassium phosphate buffered, pH 7.5

CROSSTALK

RFP/OD600 in BLR(DE3) in LB, 18h post-induction, pBbE6a construct

	IPTG(100uM)	IPTG(100uM) +aTc(400nM)	IPTG(100uM) +Arabinose(20mM)	IPTG(100uM) +Propionate(20mM)	Un-induced
pLlacO-1	100.0% (+/-3.8)	138.5% (+/-0.9)	84.1% (+/-5.4)	138.7% (+/-5.1)	0.0% (+/-0.0)

pBbA7

IPTG inducible promoter system

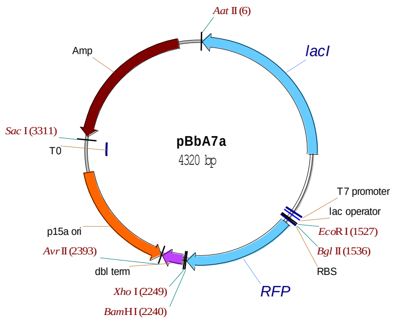

Constructs available	Freezer location (-80)
pBbA7a-RFP	2477
pBbA7k-RFP	2483
pBbA7c-RFP	2490

A = p15A ori (8-10 copies per cell) 7 = pT7
experiments represented on this datasheet were performed using pBbA7a-RFP
pBbE5a-RFP in BLR(DE3) in LB induced (100mM IPTG) was used as control

INDUCER DOSE RESPONSE

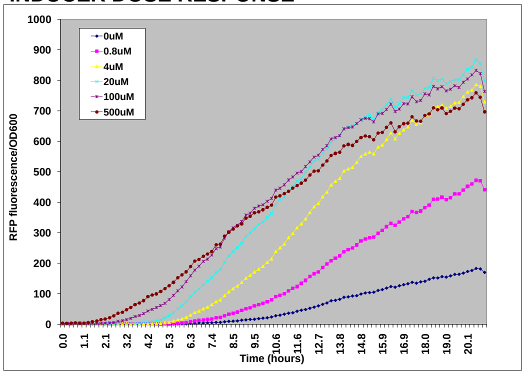

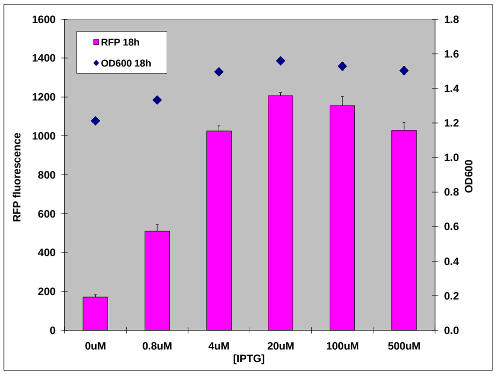

BLR(DE3) in LB, 30°C

STRAIN and MEDIA DEPENDENCE

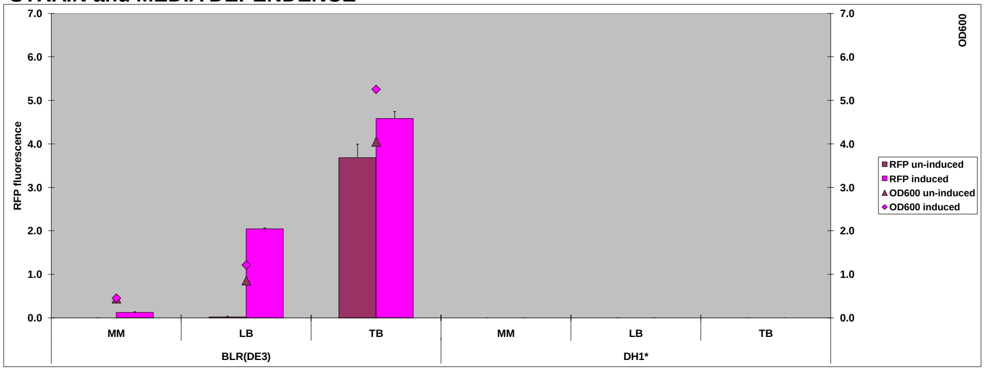

3ml cultures grown in test tubes, induced with 100uM IPTG, grown at 30°C post-induction, measurements taken in Tecan 18h post-induction
MM media is supplemented with 0.5% glucose, TB media is supplemented with 2% glycerol
RFP and OD normalized to pBbE5a-RFP in BLR(DE3) in LB induced (100uM IPTG)
*DH1 does not contain the gene encoding T7 polymerase and therefore RFP cannot be expressed under the T7 promoter in DH1, experiments were not performed

CATABOLITE REPRESSION

RFP/OD600 in BLR(DE3) as a percentage of induced without glucose, 18h post-induction

	LB	LB*+1%glucose	MM	MM+1%glucose	TB	TB*+1%glucose
pBbA7a induced	100.0% (+/-1.3)	56.6% (+/-0.6)	100.0% (+/-0.0)	41.0% (+/-0.0)	100.0% (+/-19.2)	91.6% (+/-6.8)
pBbA7a un-induced	0.2% (+/-0.3)	0.4% (+/-0.0)	0.0% (+/-0.0)	0.0% (+/-0.0)	119.2% (+/-23.0)	2.1% (+/-0.4)

*100mM potassium phosphate buffered, pH 7.5

CROSSTALK

RFP/OD600 in BLR(DE3) in LB, 18h post-induction, pBbE7a construct

	IPTG(100uM)	IPTG(100uM) +aTc(400nM)	IPTG(100uM) +Arabinose(20mM)	IPTG(100uM) +Propionate(20mM)	Un-induced
pT7	100.0% (+/-3.2)	103.8% (+/-4.9)	87.6% (+/-0.3)	101.0% (+/-0.6)	0.4% (+/-0.7)

pBbB7

IPTG inducible promoter system

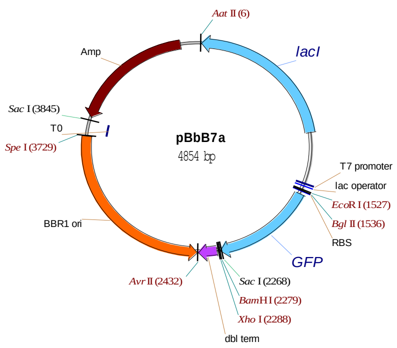

Constructs available	Freezer location (-80)
pBbB7a-GFP	2635
pBbB7k-GFP	2643
pBbB7c-GFP	2651

B = BBR1 ori (17-20 copies per cell) 7 = pT7
experiments represented on this datasheet were performed using pBbB7a-GFP
pBbE5a-GFP in BLR(DE3) in LB induced (100mM IPTG) was used as control

INDUCER DOSE RESPONSE

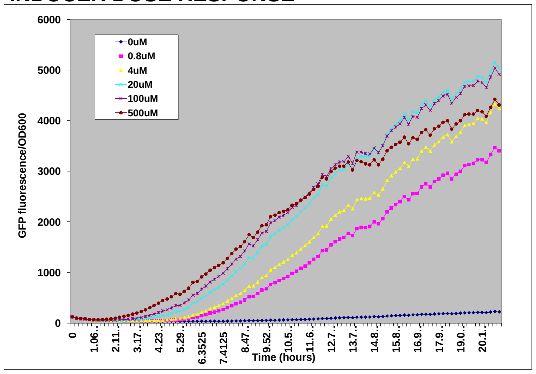

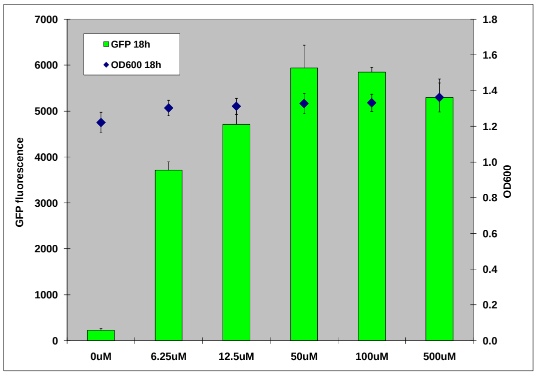

BLR(DE3) in LB, 30°C

STRAIN and MEDIA DEPENDENCE

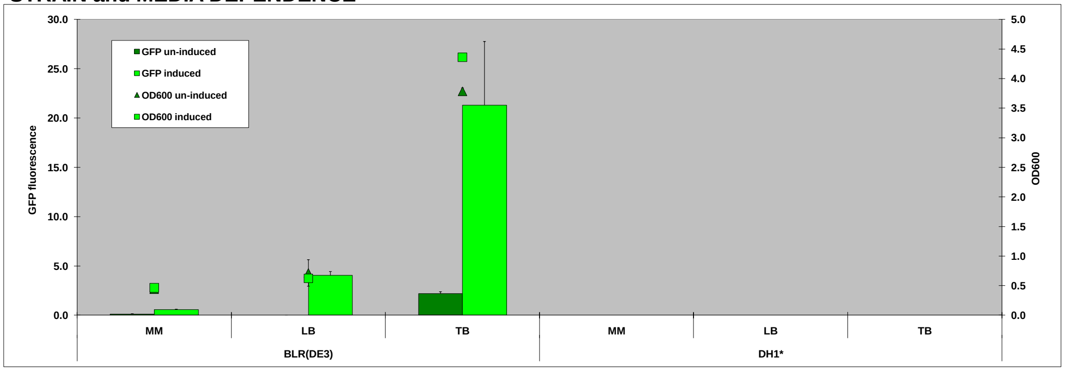

3ml cultures grown in test tubes, induced with 100uM IPTG, grown at 30°C post-induction, measurements taken in Tecan 18h post-induction
MM media is supplemented with 0.5% glucose, TB media is supplemented with 2% glycerol
GFP and OD normalized to pBbE5a-GFP in BLR(DE3) in LB induced (100uM IPTG)
*DH1 does not contain the gene encoding T7 polymerase and therefore RFP cannot be expressed under the T7 promoter in DH1, experiments were not performed

CATABOLITE REPRESSION

RFP/OD600 in BLR(DE3) as a percentage of induced without glucose, 18h post-induction

	LB	LB*+1%glucose	MM	MM+1%glucose	TB	TB*+1%glucose
pBbB7a induced	100.0% (+/-9.0)	54.9% (1.7)	100.0% (+/-17.4)	122.8% (28.4)	100.0% (+/-30.3)	97.4% (10.3)
pBbB7a un-induced	0.0% (+/-0.0)	1.1% (+/-0.5)	23.6% (+/-5.6)	16.3% (+/-6.5)	11.8% (+/-1.2)	0.9% (+/-0.2)

*100mM potassium phosphate buffered, pH 7.5

CROSSTALK

RFP/OD600 in BLR(DE3) in LB, 18h post-induction, pBbE7a construct

	IPTG(100uM)	IPTG(100uM) +aTc(400nM)	IPTG(100uM) +Arabinose(20mM)	IPTG(100uM) +Propionate(20mM)	Un-induced
pT7	100.0% (+/-3.2)	103.8% (+/-4.9)	87.6% (+/-0.3)	101.0% (+/-0.6)	0.4% (+/-0.7)

pBbE7

IPTG inducible promoter system

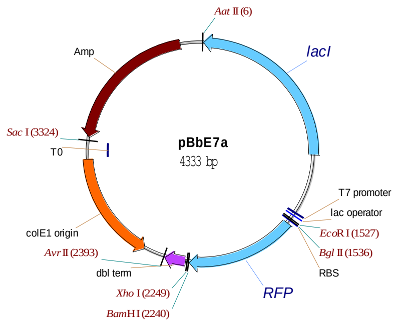

Constructs available	Freezer location (-80)
pBbE7a-RFP	2487
pBbE7k-RFP	2496
pBbE7c-RFP	2464

E = colE1 ori (20-30 copies per cell) 7 = pT7
experiments represented on this datasheet were performed using pBbE7a-RFP
pBbE5a-RFP in BLR(DE3) in LB induced (100mM IPTG) was used as control

INDUCER DOSE RESPONSE

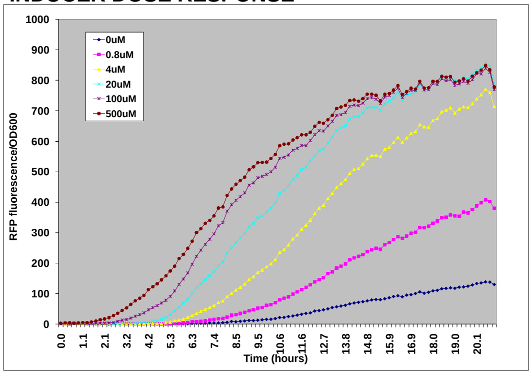

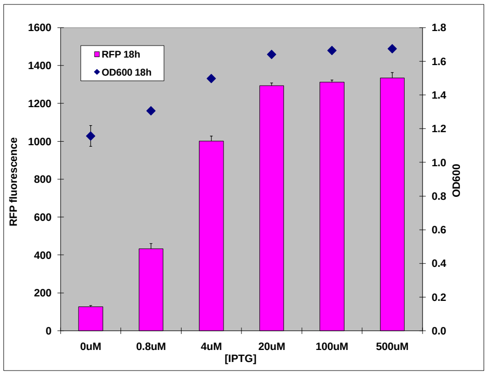

BLR(DE3) in LB, 30°C

STRAIN and MEDIA DEPENDENCE

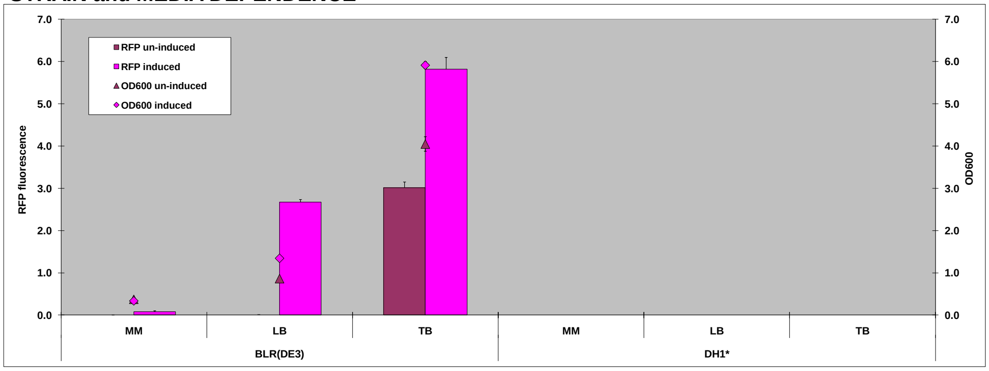

3ml cultures grown in test tubes, induced with 100uM IPTG, grown at 30°C post-induction, measurements taken in Tecan 18h post-induction
MM media is supplemented with 0.5% glucose, TB media is supplemented with 2% glycerol
RFP and OD normalized to pBbE5a-RFP in BLR(DE3) in LB induced (100uM IPTG)
*DH1 does not contain the gene encoding T7 polymerase and therefore RFP cannot be expressed under the T7 promoter in DH1, experiments were not performed

CATABOLITE REPRESSION

RFP/OD600 in BLR(DE3) as a percentage of induced without glucose, 18h post-induction

	LB	LB*+1%glucose	MM	MM+1%glucose	TB	TB*+1%glucose
pBbE7a induced	100.0% (0.8)	53.4% (+/-7.3)	100.0% (+/-0.0)	21.3% (+/-0.0)	100.0% (6.9)	91.2% (+/-7.1)
pBbE7a un-induced	0.4% (+/-0.3)	0.4% (+/-0.0)	0.0% (+/-0.0)	0.0% (+/-0.0)	110.3% (+/-14.0)	2.6% (+/-0.4)

*100mM potassium phosphate buffered, pH 7.5

CROSSTALK

RFP/OD600 in BLR(DE3) in LB, 18h post-induction, pBbE7a construct

	IPTG(100uM)	IPTG(100uM) +aTc(400nM)	IPTG(100uM) +Arabinose(20mM)	IPTG(100uM) +Propionate(20mM)	Un-induced
pT7	100.0% (+/-3.2)	103.8% (+/-4.9)	87.6% (+/-0.3)	101.0% (+/-0.6)	0.4% (+/-0.7)

pBbS7

IPTG inducible promoter system

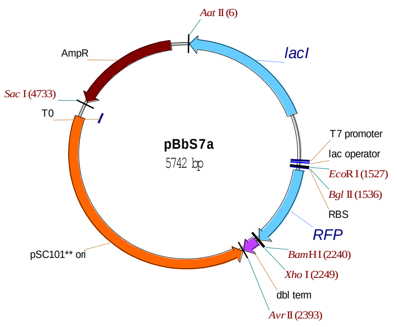

Constructs available	Freezer location (-80)
pBbS7a-RFP	2547
pBbS7k-RFP	2555
pBbS7c-RFP	2563

S = SC101 ori (4-6 copies per cell) 7 = pT7
experiments represented on this datasheet were performed using pBbS7a-RFP
pBbE5a-RFP in BLR(DE3) in LB induced (100mM IPTG) was used as control

INDUCER DOSE RESPONSE

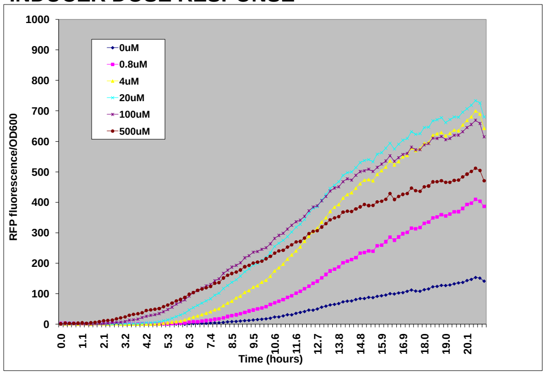

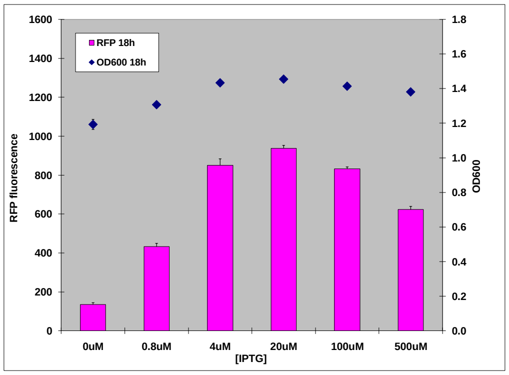

BLR(DE3) in LB, 30°C

STRAIN and MEDIA DEPENDENCE

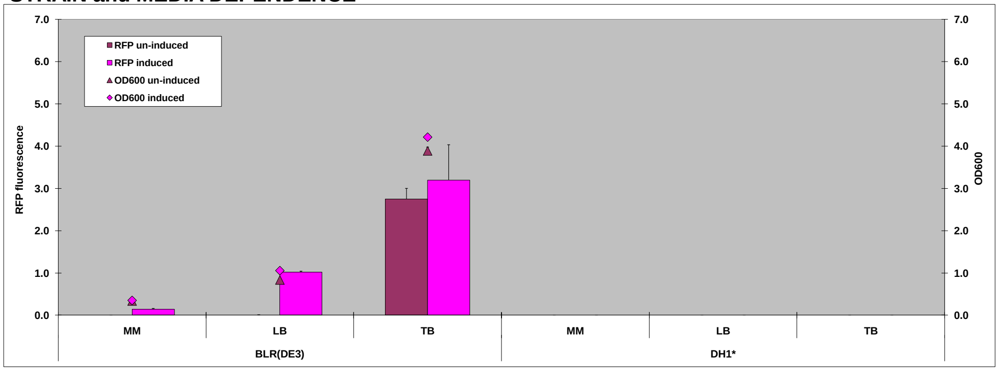

3ml cultures grown in test tubes, induced with 100uM IPTG, grown at 30°C post-induction, measurements taken in Tecan 18h post-induction
MM media is supplemented with 0.5% glucose, TB media is supplemented with 2% glycerol
RFP and OD normalized to pBbE5a-RFP in BLR(DE3) in LB induced (100uM IPTG)
*DH1 does not contain the gene encoding T7 polymerase and therefore RFP cannot be expressed under the T7 promoter in DH1, experiments were not performed

CATABOLITE REPRESSION

RFP/OD600 in BLR(DE3) as a percentage of induced without glucose, 18h post-induction

	LB	LB*+1%glucose	MM	MM+1%glucose	TB	TB*+1%glucose
pBbS7a induced	100.0% (+/-1.8)	64.6% (+/-0.8)	100.0% (+/-0.0)	40.2% (+/-0.0)	100.0% (+/-11.4)	84.7% (+/-10.9)
pBbS7a un-induced	0.3% (+/-0.5)	0.6% (+/-0.0)	0.0% (+/-0.0)	0.0% (+/-0.0)	155.1% (+/-19.8)	3.5% (+/-0.8)

*100mM potassium phosphate buffered, pH 7.5

CROSSTALK

RFP/OD600 in BLR(DE3) in LB, 18h post-induction, pBbE7a construct

	IPTG(100uM)	IPTG(100uM) +aTc(400nM)	IPTG(100uM) +Arabinose(20mM)	IPTG(100uM) +Propionate(20mM)	Un-induced
pT7	100.0% (+/-3.2)	103.8% (+/-4.9)	87.6% (+/-0.3)	101.0% (+/-0.6)	0.4% (+/-0.7)

pBbA8

Arabinose inducible promoter system

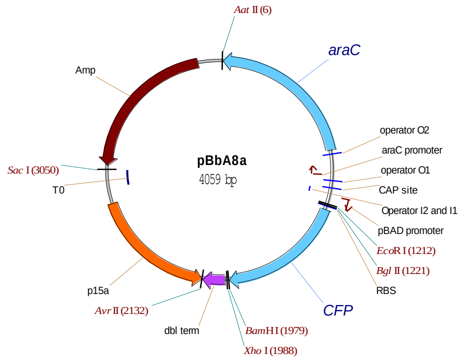

Constructs available	Freezer location (-80)
pBbA8a-RFP	2480
pBbA8k-RFP	2486
pBbA8c-RFP	2493

A = p15A ori (8-10 copies per cell) 8 = pBad
experiments represented on this datasheet were performed using pBbA8a-RFP
pBbE5a-RFP in BLR(DE3) in LB induced (100mM IPTG) was used as control

INDUCER DOSE RESPONSE

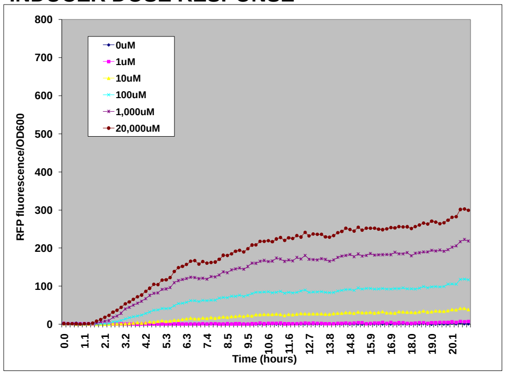

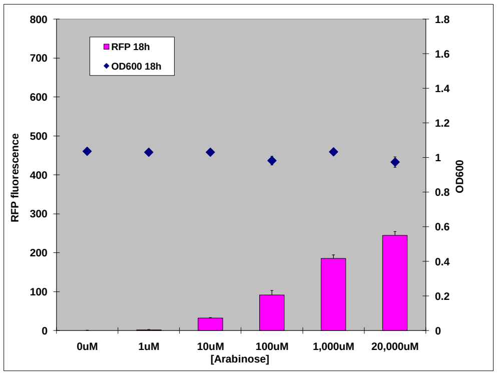

BLR(DE3) in LB, 30°C

STRAIN and MEDIA DEPENDENCE

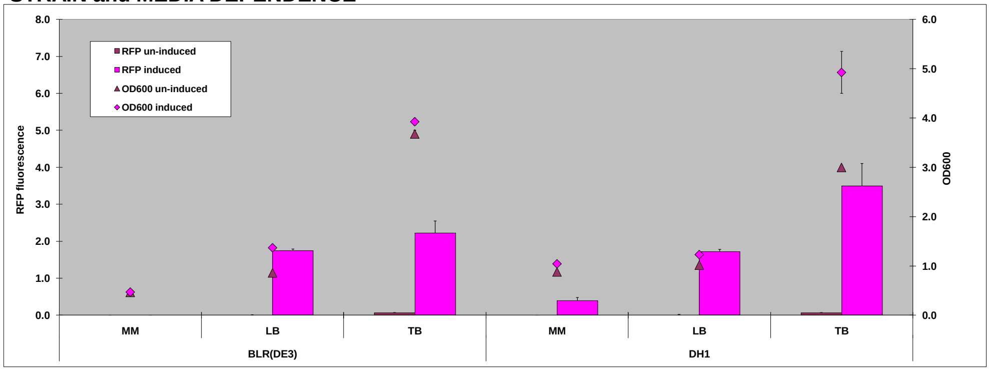

3ml cultures grown in test tubes, induced with 20mM arabinose, grown at 30°C post-induction, measurements taken in Tecan 18h post-induction
MM media is supplemented with 0.5% glucose, TB media is supplemented with 2% glycerol
RFP and OD normalized to pBbE5a-RFP in BLR(DE3) in LB induced (100uM IPTG)

CATABOLITE REPRESSION

RFP/OD600 in BLR(DE3) as a percentage of induced without glucose, 18h post-induction

	LB	LB*+1%glucose	MM	MM+1%glucose	TB	TB*+1%glucose
pBbA8a induced	100.0% (+/-10.0)	58.2% (+/-0.0)	N/A**	N/A**	100.0% (+/-15.2)	90.4% (+/-4.7)
pBbA8a un-induced	0.0% (+/-0.0)	0.0% (+/-0.0)	N/A**	N/A**	3.0% (+/-0.6)	2.3% (+/-0.5)

*100mM potassium phosphate buffered, pH 7.5
**no RFP fluorescence detected

CROSSTALK

RFP/OD600 in BLR(DE3) in LB, 18h post-induction, pBbE8a construct

	Arabinose(20mM)	Arabinose(20mM) +IPTG(100uM)	Arabinose(20mM) +aTc(400nM)	Arabinose(20mM) +Propionate(20mM)	Un-induced
pBad	100.0% (+/-2.5)	102.7% (+/-1.0)	100.1% (+/-1.5)	112.5% (+/-2.1)	0.0% (+/-0.0)

pBbB8

Arabinose inducible promoter system

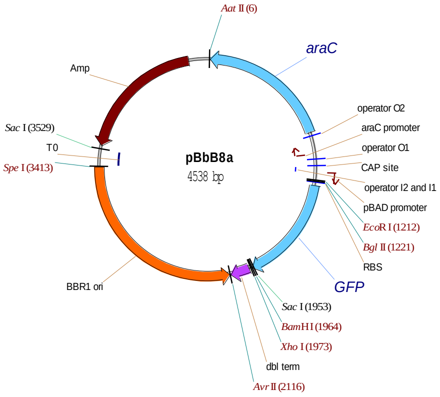

Constructs available	Freezer location (-80)
pBbB8a-GFP	2636
pBbB8k-GFP	2644
pBbB8c-GFP	2652

B = BBR1 ori (17-20 copies per cell) 8 = pBad
experiments represented on this datasheet were performed using pBbB8a-GFP
pBbE5a-GFP in BLR(DE3) in LB induced (100mM IPTG) was used as control

INDUCER DOSE RESPONSE

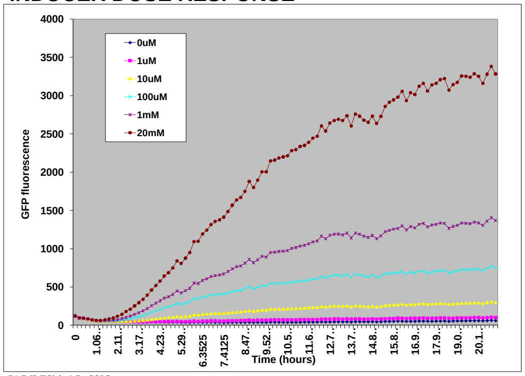

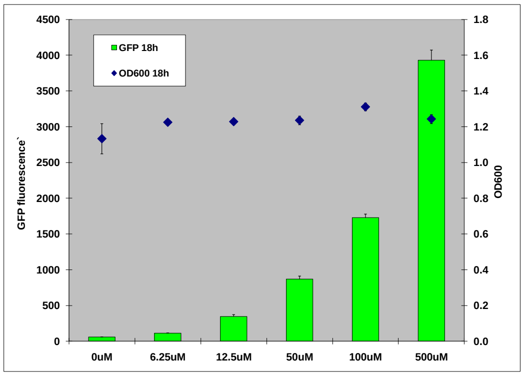

BLR(DE3) in LB, 30°C

STRAIN and MEDIA DEPENDENCE

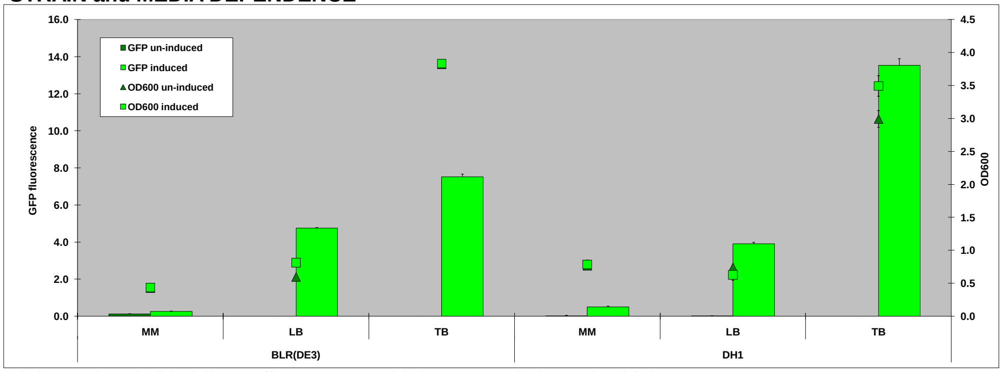

3ml cultures grown in test tubes, induced with 20mM arabinose, grown at 30°C post-induction, measurements taken in Tecan 18h post-induction
MM media is supplemented with 0.5% glucose, TB media is supplemented with 2% glycerol
GFP and OD normalized to pBbE5a-GFP in BLR(DE3) in LB induced (100uM IPTG)

CATABOLITE REPRESSION

RFP/OD600 in BLR(DE3) as a percentage of induced without glucose, 18h post-induction

	LB	LB*+1%glucose	MM	MM+1%glucose	TB	TB*+1%glucose
pBbB8a induced	100.0% (+/-2.7)	88.3% (+/-0.8)	100.0% (+/-18.3)	138.0% (+/-30.9)	100.0% (+/-1.1)	166.0% (+/-22.9)
pBbB8a un-induced	0.0% (+/-0.0)	1.1% (+/-0.0)	49.1% (+/-5.0)	38.0% (+/-3.3)	0.0% (+/-0.0)	1.2% (+/-0.6)

*100mM potassium phosphate buffered, pH 7.5

CROSSTALK

RFP/OD600 in BLR(DE3) in LB, 18h post-induction, pBbE8a construct

	Arabinose(20mM)	Arabinose(20mM) +IPTG(100uM)	Arabinose(20mM) +aTc(400nM)	Arabinose(20mM) +Propionate(20mM)	Un-induced
pBad	100.0% (+/-2.5)	102.7% (+/-1.0)	100.1% (+/-1.5)	112.5% (+/-2.1)	0.0% (+/-0.0)

pBbE8

Arabinose inducible promoter system

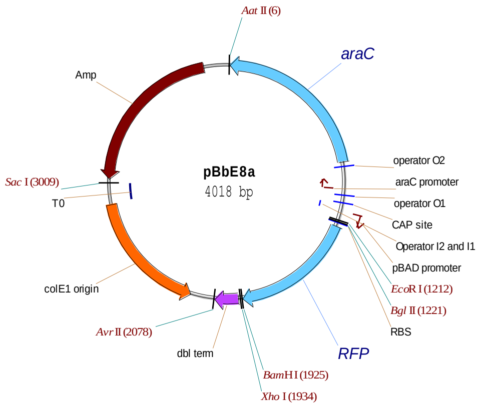

Constructs available	Freezer location (-80)
pBbE8a-RFP	2470
pBbE8k-RFP	2499
pBbE8c-RFP	2500

E= colE1 ori (20-30 copies per cell) 8 = pBad
experiments represented on this datasheet were performed using pBbE8a-RFP
pBbE5a-RFP in BLR(DE3) in LB induced (100mM IPTG) was used as control

INDUCER DOSE RESPONSE

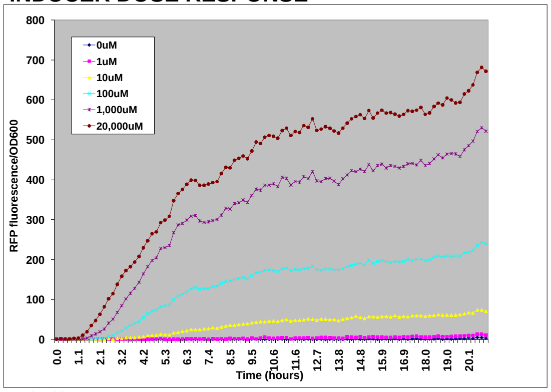

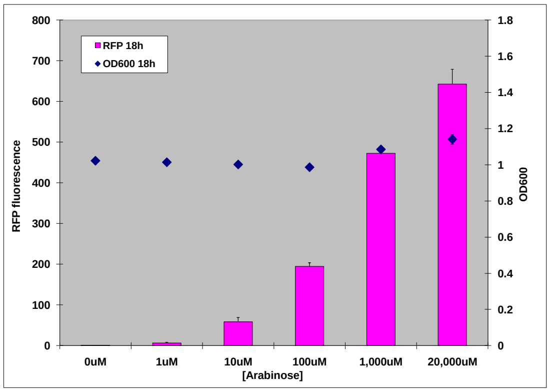

BLR(DE3) in LB, 30°C

STRAIN and MEDIA DEPENDENCE

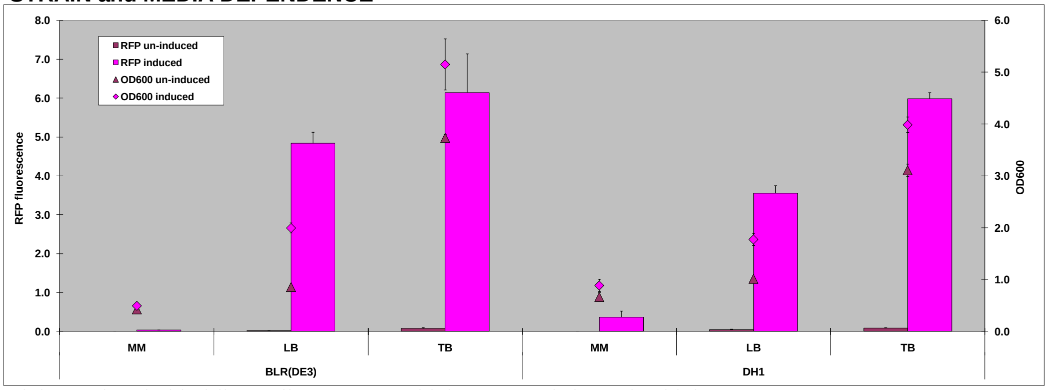

3ml cultures grown in test tubes, induced with 20mM arabinose, grown at 30°C post-induction, measurements taken in Tecan 18h post-induction
MM media is supplemented with 0.5% glucose, TB media is supplemented with 2% glycerol
RFP and OD normalized to pBbE5a-RFP in BLR(DE3) in LB induced (100uM IPTG)

CATABOLITE REPRESSION

RFP/OD600 in BLR(DE3) as a percentage of induced without glucose, 18h post-induction

	LB	LB*+1%glucose	MM	MM+1%glucose	TB	TB*+1%glucose
pBbE8a induced	100.0% (+/-9.1)	64.8% (2.9)	100.0% (+/-6.0)	85.1% (52.8)	100.0% (+/-6.6)	77.8% (8.9)
pBbE8a un-induced	0.2% (+/-0.4)	0.0% (+/-0.0)	0.0% (+/-0.0)	0.0% (+/-0.0)	1.7% (+/-0.5)	0.9% (+/-0.5)

*100mM potassium phosphate buffered, pH 7.5

CROSSTALK

RFP/OD600 in BLR(DE3) in LB, 18h post-induction, pBbE8a construct

	Arabinose(20mM)	Arabinose(20mM) +IPTG(100uM)	Arabinose(20mM) +aTc(400nM)	Arabinose(20mM) +Propionate(20mM)	Un-induced
pBad	100.0% (+/-2.5)	102.7% (+/-1.0)	100.1% (+/-1.5)	112.5% (+/-2.1)	0.0% (+/-0.0)

pBbS8

Arabinose inducible promoter system

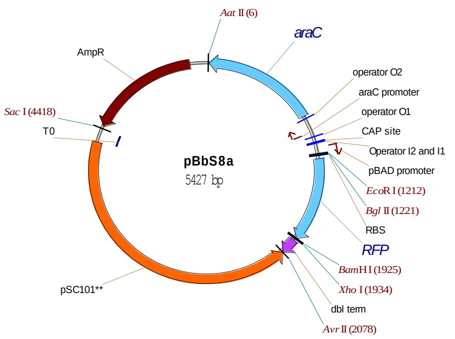

Constructs available	Freezer location (-80)
pBbS8a-RFP	2550
pBbS8k-RFP	2558
pBbS8c-RFP	2566

S = SC101 ori (4-6 copies per cell) 8 = pBad
experiments represented on this datasheet were performed using pBbS8a-RFP
pBbE5a-RFP in BLR(DE3) in LB induced (100mM IPTG) was used as control

INDUCER DOSE RESPONSE

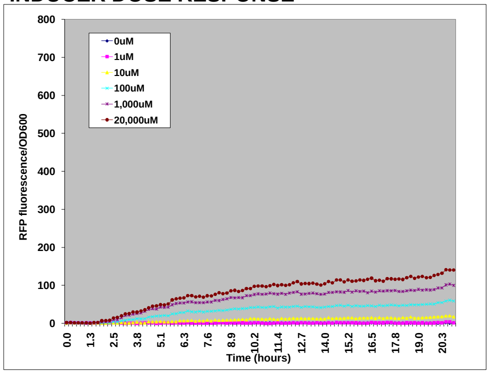

BLR(DE3) in LB, 30°C

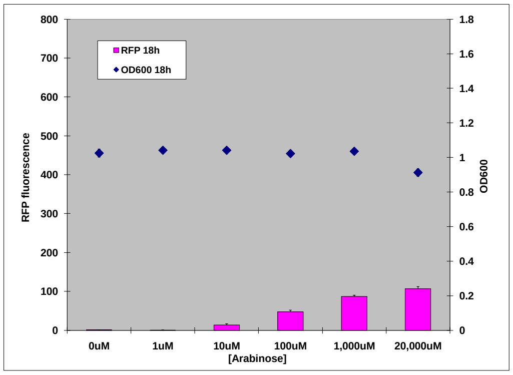

STRAIN and MEDIA DEPENDENCE

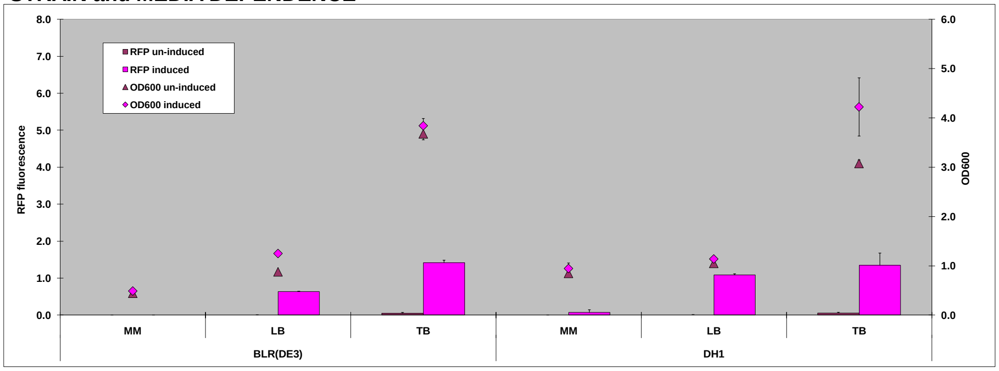

3ml cultures grown in test tubes, induced with 20mM arabinose, grown at 30°C post-induction, measurements taken in Tecan 18h post-induction
MM media is supplemented with 0.5% glucose, TB media is supplemented with 2% glycerol
RFP and OD normalized to pBbE5a-RFP in BLR(DE3) in LB induced (100uM IPTG)

CATABOLITE REPRESSION

RFP/OD600 in BLR(DE3) as a percentage of induced without glucose, 18h post-induction

	LB	LB*+1%glucose	MM	MM+1%glucose	TB	TB*+1%glucose
pBbS8a induced	100.0% (+/-15.4)	55.6% (+/-4.0)	N/A**	N/A**	100.0% (+/-1.4)	76.6% (+/-13.0)
pBbS8a un-induced	0.0% (+/-0.0)	0.0% (+/-0.0)	N/A**	N/A**	3.2% (+/-2.0)	4.3% (+/-1.4)

*100mM potassium phosphate buffered, pH 7.5
**no RFP expression detected

CROSSTALK

RFP/OD600 in BLR(DE3) in LB, 18h post-induction, pBbE8a construct

	Arabinose(20mM)	Arabinose(20mM) +IPTG(100uM)	Arabinose(20mM) +aTc(400nM)	Arabinose(20mM) +Propionate(20mM)	Un-induced
pBad	100.0% (+/-2.5)	102.7% (+/-1.0)	100.1% (+/-1.5)	112.5% (+/-2.1)	0.0% (+/-0.0)
