# Supplementary material for: BglBrick vectors and datasheets: A synthetic biology platform for gene expression
Source: J Biol Eng. 2011 Sep 20;5:12. doi: 10.1186/1754-1611-5-12 (PMC3189095; doi:10.1186/1754-1611-5-12)
Supplement: Additional file 2 — Method for the preparation of promoter-less BglBrick vectors. MS Word file with experimental details for the preparation of 12 promoter-less BglBrick vectors. [file 1754-1611-5-12-S2.DOCX]

Supplementary Material

**Method to create promoter-less BglBrick Vectors**

The 12 vectors containing *araC* and the P_BAD_ promoter (pBbX8y-RFP, “X” describes the type of replication origin used and “y” describes the antibiotic resistance marker used) were used as a starting point for creating the promoter-less BglBrick vectors. The portion of each vector containing the antibiotic resistance gene and the origin of replication was amplified by PCR with Phusion DNA polymerase (New England BioLabs, F-530) and the primers shown below.

| **Primer Name** | **Sequence** | **Templates Used With** |
| --- | --- | --- |
| pBbF | GGATCCTAACTCGAGTAAGGATCTCCAGG | pBbX8y-RFP |
| pBbaR | AGATCTCATGAATTCAGGTGGCACTTTTCGGGG | pBbX8a-RFP |
| pBbcR | AGATCTCATGAATTCGATATCTGGCGAAAATGAGAC | pBbX8c-RFP |
| pBbkR | AGATCTCATGAATTCGGAATTGCCAGCTGGGGC | pBbX8k-RFP |

After thermocycling, 1 FDU of DpnI (Fermentas, ER1703) was added to each PCR reaction and they were incubated at 37°C for 1 hour. The desired PCR products were then gel extracted using a QIAquick Gel Extraction Kit (Qiagen 28704). An overnight blunt-end ligation was then performed at 22°C using 1U T4 ligase (Fermentas EL0011). The ligation product was then transformed into chemically competent DH10B. Plasmids were then purified from these strains using QIAprep Spin Miniprep Kit (Qiagen 27104). The resulting plasmids, called pBbX0y, contain an antibiotic resistance gene, an origin of replication, and a BglBrick cloning site (EcoRI-BglII-BamHI-XhoI).

To facilitate easy cloning and screening for successful gene insertion, a constitutive RFP cassette was cloned into the BglBrick cloning site. The plasmid BBa_J61002-BBa_J23102 (see partsregistry.org for sequence information) was obtained from J.C. Anderson. The constitutive RFP cassette from this plasmid was amplified and BglBrick compatible restriction sites were added using PCR with Phusion DNA polymerase and the primers GTCAACTGGAATTCATGAGATCTTTGACAGCTAGCTCAGTCCTAG and CAGTTGACCTCGAGTTAGGATCCCGGCCGCTTCTAGTATATAAACGC. The PCR product was purified using QIAquick PCR Purification Kit (Qiagen 28104). The purified plasmid was then incubated for 1 hour at 37°C with 1 FDU of DpnI, 1 FDU of EcoRI (Fermentas, FD0274) and 1 FDU of BamHI (Fermentas, FD0054). A second clean up was performed with the QIAquick PCR Purification Kit. The vectors (pBbX0y, “X” describes the type of replication origin used and “y” describes the antibiotic resistance marker used) were also digested for 1 hour at 37°C with 1 FDU EcoRI and 1 FDU BamHI before purification using the QIAquick PCR Purification Kit. The RFP cassette was then ligated with each of the 12 vectors using 2.5U of T4 ligase at 22°C for 10 minutes before transformation into chemically competent DH10B cells. The resulting set of vectors are called pBbX0y-RFP (“X” describes the type of replication origin used and “y” describes the antibiotic resistance marker used).
